# Chronic binge alcohol administration dysregulates global regulatory gene networks associated with skeletal muscle wasting in simian immunodeficiency virus-infected macaques

**DOI:** 10.1186/s12864-015-2329-z

**Published:** 2015-12-23

**Authors:** Liz Simon, Andrew D. Hollenbach, Jovanny Zabaleta, Patricia E. Molina

**Affiliations:** Department of Physiology, Louisiana State University Health Sciences Center, New Orleans, LA 70112 USA; LSUHSC-NO Comprehensive Alcohol-HIV/AIDS Research Center (CARC), 1901 Perdido Street, New Orleans, LA 70112 USA; Department of Genetics, 533 Bolivar Street, New Orleans, LA 70112 USA; Department of Pediatrics, Stanley S. Scott Cancer Center, 1700 Tulane Ave., Suite 909, New Orleans, USA

**Keywords:** Chronic binge alcohol administration, SIV, Skeletal muscle wasting, High-output array analysis, Gene regulatory networks, Epigenetic regulation

## Abstract

**Background:**

There are more than 1 million persons living with HIV/AIDS (PLWHA) in the United States and approximately 40 % of them have a history of alcohol use disorders (AUD). Chronic heavy alcohol consumption and HIV/AIDS both result in reduced lean body mass and muscle dysfunction, increasing the incidence of comorbid conditions. Previous studies from our laboratory using rhesus macaques infected with Simian Immunodeficiency Virus (SIV) demonstrated that chronic binge alcohol (CBA) administration in the absence of antiretroviral therapy exacerbates skeletal muscle (SKM) wasting at end-stage SIV disease. The aim of this study was to characterize how CBA alters global gene regulatory networks that lead to SKM wasting at end-stage disease. Administration of intragastric alcohol or sucrose to male rhesus macaques began 3 months prior to SIV infection and continued throughout the duration of study. High-output array analysis was used to determine CBA-dependent changes in mRNA expression, miRNA expression, and promoter methylation status of SKM at end-stage disease (~10 months post-SIV) from healthy control (control), sucrose-administered, SIV-infected (SUC/SIV), and CBA-administered/SIV-infected (CBA/SIV) macaques.

**Results:**

In addition to previously reported effects on the extracellular matrix and the promotion of a pro-inflammatory environment, we found that CBA adversely affects gene regulatory networks that involve “universal” cellular functions, protein homeostasis, calcium and ion homeostasis, neuronal growth and signaling, and satellite cell growth and survival.

**Conclusions:**

The results from this study provide an overview of the impact of CBA on gene regulatory networks involved in biological functions, including transcriptional and epigenetic processes, illustrating the genetic and molecular mechanisms associated with CBA-dependent SKM wasting at end-stage SIV infection.

**Electronic supplementary material:**

The online version of this article (doi:10.1186/s12864-015-2329-z) contains supplementary material, which is available to authorized users.

## Background

An estimated 1.15 million persons are living with human immunodeficiency virus (HIV)/acquired immunodeficiency syndrome (AIDS) (PLWHA) in the United States [1, 2]. With the use of antiretroviral therapy (ART), HIV is now a chronic disease with higher incidence of associated non-AIDS defining illnesses. PLWHA have a higher prevalence of alcohol use disorders (AUDs) than the general population [3]. Chronic heavy alcohol consumption accelerates the progression of HIV/AIDS and contributes to comorbid pathologies seen in PLWHA [4]. Among the different pathophysiological comorbidities that enhance the progression of the disease, decreased muscle mass/function remains a strong and consistent predictor of mortality, with the frequency of low skeletal muscle (SKM) mass detected at a much younger age in PLWHA compared to that of the general population [5, 6].

Chronic heavy alcohol consumption and HIV both result in significant SKM derangements such as atrophy, weakness, and dysfunction [5, 7–10]. However, there are few reports that describe the comorbid effects of chronic alcohol consumption and HIV on SKM biology [11–13]. Our previous studies provided evidence that chronic binge alcohol (CBA) administration accentuates metabolic derangements [11, 12, 14, 15], leading to a marked decrease in SKM mass (SAIDS wasting) and dysfunctional skeletal muscle phenotype, thereby accelerating the time to end-stage disease in SIV-infected macaques [12]. Decreased mass and dysfunctional SKM phenotypes are associated with changes in gene expression levels — changes that were functionally correlated to the generation of an inflammatory milieu, dysregulation of components of the ubiquitin proteasome system, increased proteasomal activity, depletion of SKM anti-oxidant capacity, and an increased expression of pro-fibrotic genes [13, 14]. In subsequent studies we further demonstrated the relevance of these observed gene expression changes by showing that CBA impairs the differentiation potential of myoblasts to form myotubes, suggesting that SKM regeneration is adversely affected [16]. Taken together, our studies provide strong evidence that CBA accelerates the loss of SKM mass and impairs regeneration potential, which we predict would decrease quality of life and increase morbidity and mortality among PLWHA.

In order to understand the underlying genetic and molecular mechanisms that contribute to CBA-dependent SKM wasting at end-stage SIV, we previously examined the pattern of dysregulation of several key genes involved in biological processes required for SKM homeostasis [13, 14, 16]. Although these studies were informative, they provided only part of the larger picture, since CBA/SIV produces multi-systemic and dynamic changes that disrupt finely tuned and highly integrated global gene regulatory networks. These networks include multiple molecular processes, including the initiation of transcription and epigenetic mechanisms such as methylation of promoters and the post-transcriptional regulation by microRNAs. Therefore, the global analysis of the transcriptomic and epigenomic profiles from tissues derived from CBA/SIV whole animal studies was considered to be invaluable for understanding how CBA disrupts the interaction, integration, and function of these global gene regulatory networks to contribute to SKM wasting at end-stage SIV infection.

In this study we utilized high-output microarray gene analysis using RNA and/or DNA isolated from SKM tissue derived from CBA-administered rhesus macaques at end-stage SIV infection to determine CBA-dependent changes in mRNA expression, miRNA expression, and promoter methylation status. Our results provide a model that describes the impact of CBA on global transcriptional and epigenetic networks that disrupt the complex interplay between biological functions, thereby disrupting normal SKM function. In addition to the previously described changes in expression of genes that promote a pro-inflammatory environment and impair the integrity and composition of the extracellular matrix (ECM) [13], our results showed changes in gene regulatory networks that disrupt calcium and ion homeostasis, neuromuscular junction (NMJ), satellite cell growth and survival, “universal” functions of the cell (e.g., glycolysis) and protein homeostasis. This is the first report to describe the effects of CBA on SKM gene regulatory networks, providing a more comprehensive picture of the genetic and molecular mechanisms underlying CBA-dependent SKM wasting at end-stage SIV infection.

## Results

### Chronic binge alcohol-dependent mRNA expression changes

**Fig. 1:**
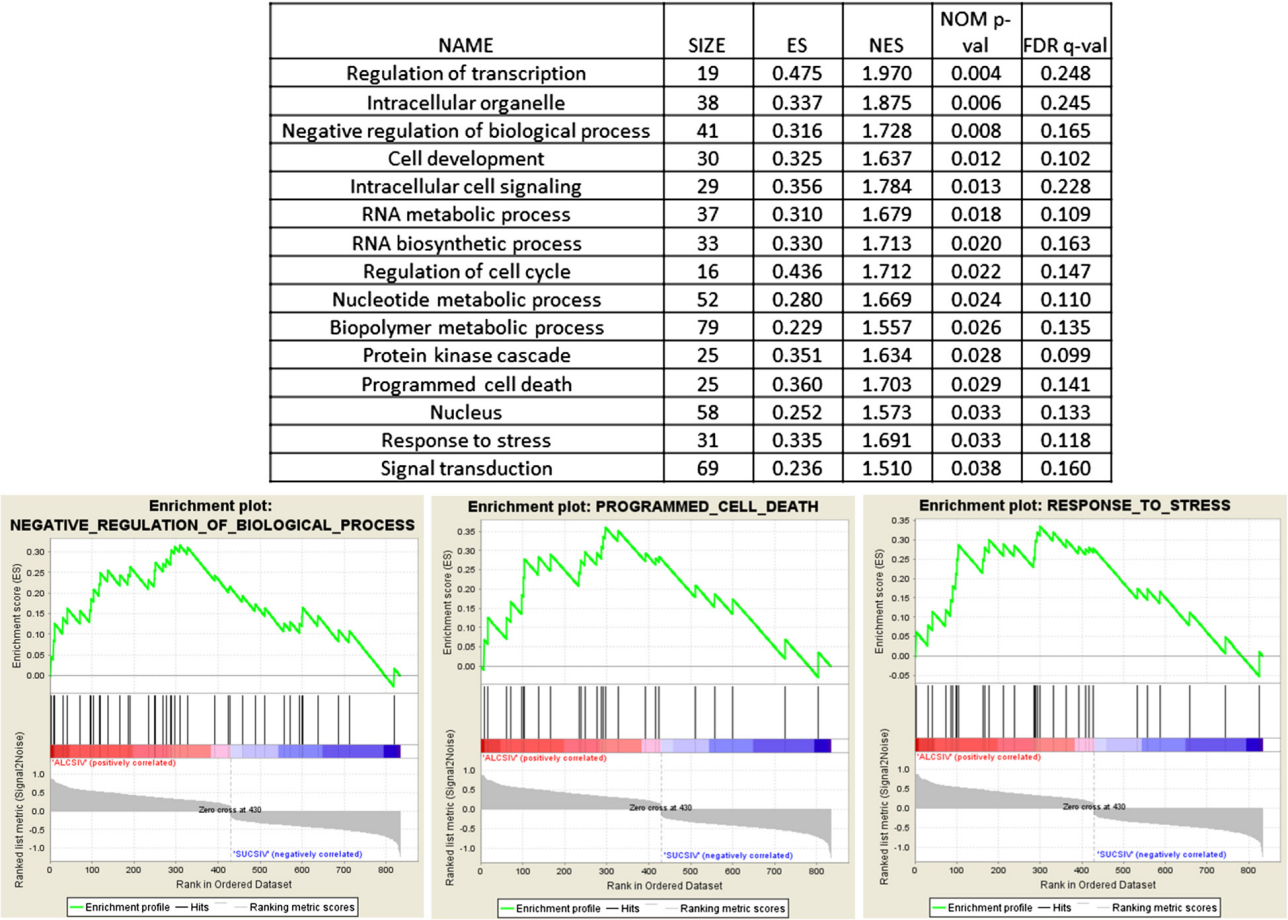
Functional enrichment of CBA-altered mRNAs at end-stage SIV disease. **a** The enriched pathways analyzed by gene set enrichment analysis revealed significant association of genes of CBA/SIV group in fifteen main gene sets. **b** Three representative plots of enriched gene sets in the CBA/SIV group. Enrichment score (ES) is the degree to which a gene set is overrepresented at the top or bottom of a ranked list of genes. NES is normalized enrichment score. Nominal p-value (NOM p-val) estimates the statistical significance of the enrichment score for a single gene set. False discovery rate (FDR) is the estimated probability that a gene set with a given NES represents a false positive finding

A total of 431 genes were significantly increased and 404 genes were significantly decreased in a CBA-dependent manner. Functional enrichment analysis was performed using GSEA as described in methods. In the CBA/SIV group, there were 74 gene sets that were upregulated among 111 gene sets. Of these 74 gene sets 36 were significant at a false discovery rate (FDR) < 25 % and of these, 27 gene sets are significantly enriched at a p-value < 0.05. (Fig. 1a, Additional file 1: Table S1). Pathways associated with transcription, programmed cell death, response to stress, protein kinase cascade and others were enriched in the CBA/SIV group (Fig. 1b). In the SUC/SIV group, there were 37 upregulated gene sets among 111 total gene sets (Additional file 1: Table S1). However, there were no gene sets that were significantly enriched according to the FDR or p-value.

In addition to previously described changes in genes affecting the inflammatory response, oxidative stress response, ECM reorganization [13], and enzymes involved in the ubiquitin mediated degradation of proteins and initiation of translation [14, 17], we found that CBA altered the expression of genes in five additional distinct biological categories: (1) “universal” cellular functions, (2) protein homeostasis, (3) calcium and ion homeostasis, (4) muscle and neuromuscular junction (NMJ) function, and (5) myogenesis, encompassing muscle satellite growth and survival.

#### “Universal” cellular functions category

One hundred twenty three genes encoding enzymes that can be viewed as having “universal” cellular functions were significantly different in the CBA/SIV group. Of these genes, 18 encode proteins important for the production of energy, including 5 of the upregulated genes required for the metabolism of alcohol and 10 of the downregulated genes involved in the glycolytic pathway and glycogen degradation, connecting glycolysis to the TCA cycle and the respiratory pathway. CBA also altered the expression of genes required for histone remodeling and modification (9 genes), multifunctional transcriptional regulators (27 genes), general kinases and phosphatases (22 genes), lipid biosynthesis and degradation (11 genes), mitochondrial function (12 genes), along with other individual genes important for the general functioning of the cell.

#### Protein homeostasis category

CBA/SIV altered the expression of 39 genes important for the synthesis of amino acids and nucleic acids, enzymes essential for the proper charging of tRNA and ribosome biogenesis, proteins utilized in the splicing and maturation of mRNA, and multifunctional transcription factors that regulate the initiation of transcription (Additional file 2: Table S2). Further, 11 genes required for the trafficking of proteins through the endoplasmic reticulum and Golgi apparatus and the glycosylation of proteins in these cellular compartments had altered expression, suggesting that the effects of CBA on protein homeostasis not only encompass the initiation of translation and ubiquitin-mediated protein degradation, but also involve all aspects from the generation of components required for synthesis to the proper post-translational modifications required for functional mature proteins.

#### Calcium and ion homeostasis category

CBA altered the expression of 25 genes important for calcium homeostasis and 23 genes that encode ion channels. We also found that CBA increased the expression of two calcium-dependent cadherins, which are important for muscle development, and one calcium-dependent gene necessary for neuronal development.

#### Muscle and NMJ function category

Among the 13 differentially expressed genes involved in muscle differentiation, one encoded a myogenic factor directly involved in promoting or inhibiting myogenic differentiation, myogenic enhancing factor 2C (MEF2C). A CBA-dependent decrease in the expression of genes required for proper contractile function, cell structure and integrity, and cellular expansion was also observed (Additional file 2: Table S2). We found an increased expression of pinin desmosome-associated protein (PNN), a gene that reverses the expression of E-cadherin, an adhesion molecule required for muscle differentiation. Consistent with increased collagen deposition and the pro-fibrotic environment in muscle wasting [13], CBA increased the expression of the gene encoding the transmembrane protein 119 (TMEM119), which drives the differentiation of myoblasts into osteoclasts, a cell type that promotes the deposition of collagen. CBA altered expression of genes important for neuronal survival and regeneration, CNS development, acetylcholine receptor expression, and neuronal organization and orientation, consistent with a CBA-mediated alteration of NMJ and normal muscle function.

#### Muscle satellite cell growth and survival category

Consistent with our previous findings that CBA impairs skeletal muscle regenerative capacity, the results from this study showed that CBA altered the expression of 38 genes that contribute to the growth, survival, and cell cycle regulation of cells. These include genes important for cell cycle progression, growth factor related signaling pathways, cellular survival and apoptosis, and additional genes (including kinases, transcription factors, and co-regulatory proteins) that mediate or contribute to cellular proliferation and/or survival. We previously published reports that demonstrate the functional relevance of many of these differentially expressed genes [13].

### Chronic binge alcohol-dependent mirna expression changes

**Fig. 2:**
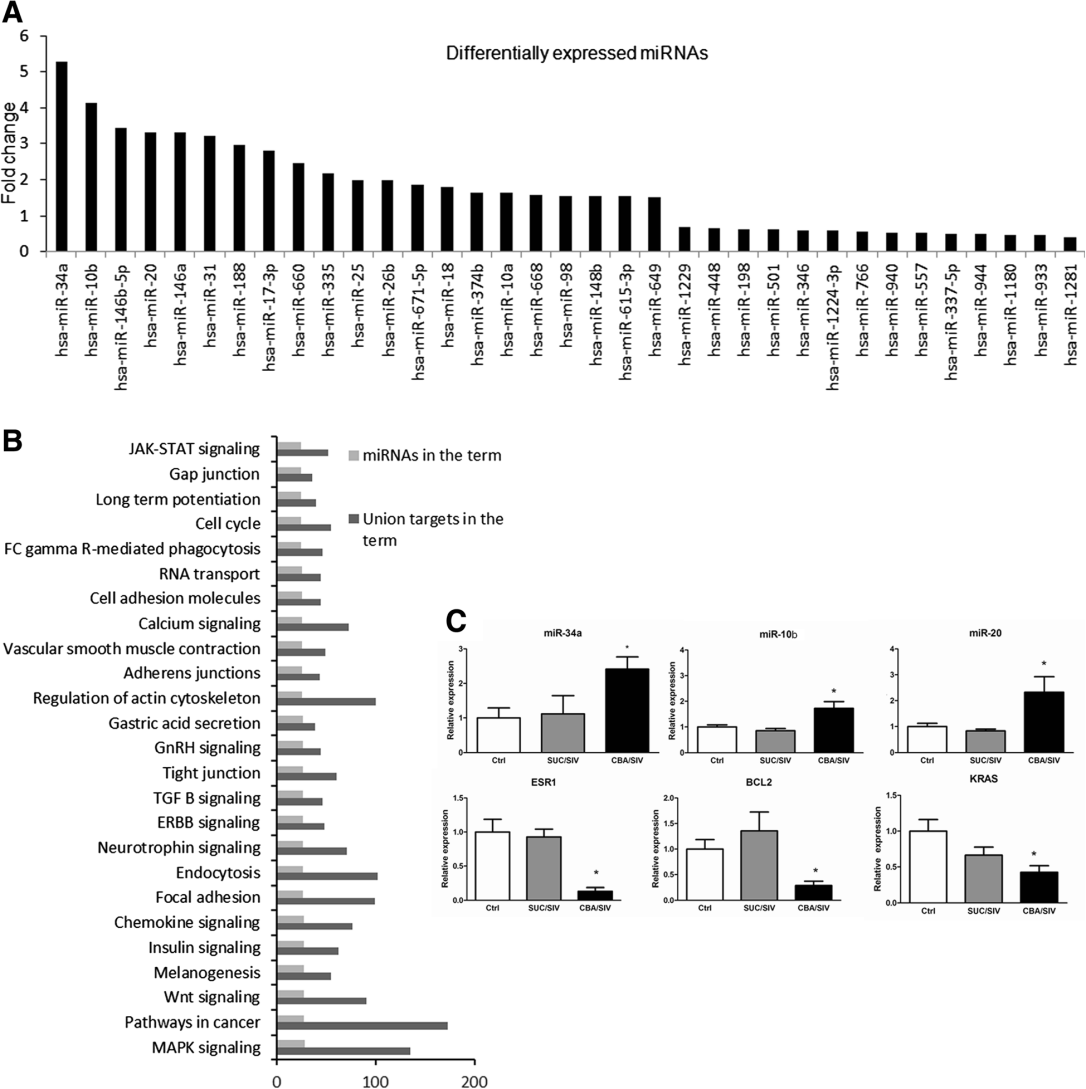
Functional enrichment of CBA-altered miRNAs at end-stage SIV disease. **a** CBA-dependent differentially expressed miRNAs. **b** Pathways enriched by predicted target genes of differentially expressed miRNAs as determined by miRSystem. **c** qPCR confirmation of miRNAs and target genes: Relative miRNA expression of miR-34a, miR-10b and miR-20 in the skeletal muscle of control (white bars), SUC/SIV (grey bars), and CBA/SIV (black bars) macaques determined by qPCR. Relative mRNA expression of ESR1, BCL2, and KRAS in the skeletal muscle of control (white bars), SUC/SIV (grey bars), and CBA/SIV (black bars) macaques determined by qPCR. Values are mean ± SEM. *p < 0.05 vs. Control and SUC/SIV

We identified 35 miRNAs whose expression changed ≥1.5-fold in a CBA-dependent manner. Of these, 14 were downregulated and 21 were upregulated (Fig. 2a). Functional enrichment of predicted target genes of differentially expressed miRNAs was analyzed using miRsystem as described in the methods section. Pathways associated with MAPK signaling, insulin signaling, neuronal signaling, and focal adhesions were enriched (Fig. 2b). The 100 most enriched pathways that included ≥20 differentially expressed miRNAs, ≥20 target genes, and had a score ≥1 are shown in Table 1. Target genes from additional pathways showed a higher representation of proteins important for essential enzymes or cofactors in ubiquitin-mediated degradation of proteins, genes involved in maintenance of the integrity or in the breakdown of the extracellular matrix and muscle contraction (Additional file 3: Table S3).

**Table 1:** CBA-dependent alterations in microRNA expression at end-stage SIV infection

Category	Term	Total genes in term	Union targets in term	miRNAs in term	Score
Reactome	Axon_guidance	266	154	28	3.983
Reactome	Developmental_biology	494	229	28	3.716
KEGG	Pathways_in_cancer	325	173	28	2.951
Reactome	L1cam_interactions	94	61	26	2.77
KEGG	Mapk_signaling_pathway	272	135	29	2.466
KEGG	Wnt_signaling_pathway	150	91	28	2.466
Go_mf	Protein_binding_transcription_factor_activity	369	164	28	2.334
Reactome	Signalling_by_ngf	221	118	26	2.243
PID	Direct_p53_effectors	137	65	27	2.062
KEGG	Focal_adhesion	199	99	27	1.992
Reactome	Hemostasis	467	191	29	1.989
KEGG	Endocytosis	201	102	27	1.944
KEGG	Prostate_cancer	89	49	26	1.941
KEGG	Neurotrophin_signaling_pathway	127	71	27	1.909
PID	Pdgfr-beta_signaling_pathway	126	69	27	1.9
PID	Regulation_of_nuclear_smad2_3_signaling	82	52	27	1.847
Reactome	Neuronal_system	289	120	28	1.837
KEGG	Glioma	65	35	27	1.836
PID	Signaling_events_mediated_by_hepatocyte_growth_factor_receptor_(c-met)	77	50	26	1.74
KEGG	Regulation_of_actin_cytoskeleton	213	100	26	1.73
PID	E2f_transcription_factor_network	73	44	25	1.717
Reactome	Transmission_across_chemical_synapses	190	85	28	1.701
PID	C-myb_transcription_factor_network	81	46	24	1.683
Reactome	Nuclear_receptor_transcription_pathway	51	27	26	1.66
PID	Ephb_forward_signaling	36	27	22	1.619
KEGG	Erbb_signaling_pathway	87	48	27	1.614
KEGG	Melanoma	71	34	27	1.594
KEGG	Melanogenesis	101	55	28	1.582
KEGG	Small_cell_lung_cancer	84	43	26	1.563
PID	Notch_signaling_pathway	59	37	25	1.55
KEGG	Tgf-beta_signaling_pathway	84	47	27	1.545
PID	Tcr_signaling_in_naive_cd4 + _t_cells	64	36	24	1.544
Reactome	Signaling_by_egfr	109	62	25	1.521
KEGG	Pancreatic_cancer	70	41	26	1.518
KEGG	Fc_gamma_r-mediated_phagocytosis	94	47	25	1.506
PID	Coregulation_of_androgen_receptor_activity	57	35	26	1.5
KEGG	Adherens_junction	73	44	26	1.499
KEGG	Tight_junction	132	61	27	1.498
PID	Shp2_signaling	54	34	25	1.485
PID	P73_transcription_factor_network	73	34	25	1.462
Reactome	Rho_gtpase_cycle	124	58	25	1.455
Reactome	Signaling_by_fgfr	114	57	26	1.447
KEGG	Chronic_myeloid_leukemia	73	39	25	1.432
KEGG	Renal_cell_carcinoma	70	40	25	1.428
PID	Ap-1_transcription_factor_network	69	41	25	1.41
Reactome	G1_phase	38	23	22	1.387
KEGG	Dilated_cardiomyopathy	90	42	24	1.384
Reactome	Signaling_by_interleukins	106	47	25	1.371
Reactome	Downstream_signal_transduction	93	53	25	1.354
KEGG	Bacterial_invasion_of_epithelial_cells	70	39	24	1.349
Reactome	Adaptive_immune_system	482	162	28	1.347
PID	Integrins_in_angiogenesis	74	46	25	1.342
KEGG	Ubiquitin_mediated_proteolysis	135	70	24	1.337
PID	Hif-1-alpha_transcription_factor_network	65	35	26	1.337
KEGG	Oocyte_meiosis	112	52	27	1.334
PID	Cxcr4-mediated_signaling_events	102	54	25	1.323
Reactome	Cd28_co-stimulation	31	20	21	1.316
PID	Role_of_calcineurin-dependent_nfat_signaling_in_lymphocytes	55	35	23	1.31
KEGG	Cell_cycle	124	55	25	1.29
PID	Cdc42_signaling_events	70	40	25	1.29
KEGG	P53_signaling_pathway	68	38	24	1.289
PID	Posttranslational_regulation_of_adherens_junction_stability_and_dissassembly	48	29	24	1.28
Reactome	Platelet_activation_signaling_and_aggregation	205	85	27	1.278
PID	Regulation_of_retinoblastoma_protein	64	35	27	1.275
PID	Bcr_signaling_pathway	68	36	25	1.274
PID	Signaling_events_regulated_by_ret_tyrosine_kinase	38	22	22	1.266
Reactome	Circadian_clock	33	20	24	1.254
KEGG	Long-term_potentiation	70	40	25	1.252
Reactome	Cytokine_signaling_in_immune_system	220	77	27	1.236
KEGG	Shigellosis	61	36	23	1.233
KEGG	Vascular_smooth_muscle_contraction	126	49	26	1.21
KEGG	Gnrh_signaling_pathway	101	45	27	1.21
Reactome	Mitotic_g1-g1_s_phases	135	46	25	1.195
PID	Signaling_events_mediated_by_hdac_class_i	67	31	22	1.183
PID	Foxo_family_signaling	49	31	25	1.179
PID	Signaling_events_mediated_by_VEGFR	68	41	24	1.163
KEGG	Insulin_signaling_pathway	137	63	28	1.153
PID	Igf1_pathway	29	21	23	1.147
Reactome	Cell_cycle_mitotic	330	95	27	1.146
PID	Validated_targets_of_c-myc_transcriptional_repression	63	31	25	1.137
Reactome	P75_ntr_receptor-mediated_signalling	86	40	23	1.135
KEGG	Colorectal_cancer	62	38	25	1.13
Reactome	Cell_death_signalling_via_nrage_nrif_and_nade	64	32	23	1.126
Reactome	Transmembrane_transport_of_small_molecules	427	145	28	1.125
PID	Atf-2_transcription_factor_network	58	34	25	1.124
PID	Ifn-gamma_pathway	42	30	24	1.12
Reactome	Metabolism_of_liPIDs_and_lipoproteins	292	89	27	1.119
KEGG	Calcium_signaling_pathway	177	73	26	1.113
Reactome	G_alpha_(12_13)_signalling_events	77	36	22	1.111
PID	Syndecan-1-mediated_signaling_events	46	27	22	1.11
PID	Lpa_receptor_mediated_events	66	37	23	1.109
Reactome	Fatty_acid_triacylglycerol_and_ketone_body_metabolism	112	40	21	1.096
PID	E-cadherin signaling in_the_nascent_adherens_junction	38	27	22	1.094
PID	Bmp_receptor_signaling	42	28	21	1.09
KEGG	Chemokine_signaling_pathway	189	77	28	1.088
Reactome	Nrage_signals_death_through_jnk	47	25	23	1.086
Reactome	Cell-cell_communication	129	60	25	1.083
KEGG	T_cell_receptor_signaling_pathway	108	50	25	1.083
Reactome	Antigen_processing_ubiquitination_proteasome_degradation	213	86	26	1.078
PID	Fgf_signaling_pathway	59	35	24	1.061

Expression values for 3 upregulated candidate miRNAs (miR-34a, miR-10b and miR-20) in the microarray were confirmed by qPCR (Fig. 2c). We observed significant CBA/SIV-dependent changes in miRNA gene expression consistent with our microarray results. We also determined expression levels of 3 mRNAs: (1) estrogen receptor-alpha (ESR1), which demonstrated CBA/SIV-dependent changes in the microarray results and serves as a validated target of miR-34a and −20, (2) B-cell lymphoma-2 (BCl-2), a validated target of miR-34a, and (3) Kirsten Rat Sarcoma (KRAS), a validated target of miR-18. We found significant changes in ESR1 that were consistent with our microarray, and all mRNA showed significant changes with the expected inverse correlation to miRNA changes (Fig. 2c).

As described in the methods, we also analyzed biological functions of target genes of differentially regulated miRNAs. Genes targeted by downregulated miRNAs are involved in inflammatory/immune response, general cellular functions, neuronal function, etc. Frequently, a single gene was targeted by a miRNA within each individual biological function. Nearly 50 % of the genes targeted by upregulated miRNAs are primarily associated with five biological functional groups including (1) regulation of cell cycle progression, (2) activation of the innate immune response upon viral infection, (3) control of neuronal survival and plasticity, CNS development, and neuronal differentiation, (4) multifunctional receptors that mediate several cellular functions (e.g., proliferation, differentiation, glucose homeostasis, etc.); and (5) the Wnt, mitogen activated protein kinase (MAPK) and transforming growth factor (TGF) β signaling (Additional file 4: Table S4).

### Chronic binge alcohol-dependent methylation changes

A total of 112 genes had alcohol-dependent changes in promoter methylation of ≥1.5-fold (12 had decreased and 100 had increased promoter methylation) (Additional file 5: Table S5). Eight of the genes with decreased methylation and 16 of the genes with increased methylation correspond to genes up- or downregulated, respectively, in the mRNA gene expression array. The major BPs that genes with differential methylation expression are involved in are transmission of nerve impulse, cell communication, ion homeostasis, glucose metabolism and cellular processes (Table 2).

**Table 2:** CBA-dependent alterations in promoter methylation at end-stage SIV infection

Biological process	Count	%	*P*-Value	Benjamini
Regulation of synaptic transmission	7	0.5	0.00027	0.3
Regulation of transmission of nerve impulse	7	0.5	0.00042	0.23
Regulation of neurological system process	7	0.5	0.00052	0.15
Regulation of system process	8	0.5	0.0043	0.6
Cellular process	81	5.6	0.0057	0.65
Regulation of biological quality	19	1.3	0.0068	0.67
Alcohol metabolic process	9	0.6	0.0073	0.65
Neurotransmitter metabolic process	3	0.2	0.0091	0.69
Vesicle-mediated transport	10	0.7	0.014	0.74
Cell communication	12	0.8	0.015	0.75
Transmembrane receptor protein tyrosine kinase signaling pathway	6	0.4	0.016	0.75
Cell-cell signaling	10	0.7	0.017	0.75
Glucose metabolic process	5	0.3	0.018	0.73
Regulation of neurogenesis	5	0.3	0.024	0.81
Establishment of localization in cell	12	0.8	0.024	0.79
Enzyme linked receptor protein signaling pathway	7	0.5	0.025	0.79
Negative regulation of biological process	20	1.4	0.026	0.78
Intracellular transport	10	0.7	0.029	0.8
Cellular component biogenesis	13	0.9	0.031	0.81
Negative regulation of programmed cell death	7	0.5	0.031	0.8
Regulation of cellular component organization	8	0.5	0.031	0.79
Regulation of phosphorylation	8	0.5	0.034	0.78
Cellular ion homeostasis	7	0.5	0.037	0.8
Regulation of phosphorus metabolic process	8	0.5	0.041	0.8
Cellular localization	12	0.8	0.041	0.79
Regulation of multicellular organismal process	12	0.8	0.044	0.8
RNA metabolic process	12	0.8	0.044	0.8

Count: number of genes involved in the term; %: percentage of involved genes/total genes; P-Value: modified fisher exact P-value, EASE Score; Benjamini: adjusted P-value using Benjamini-Hochberg procedure

Examination of the genes in which an increase in promoter methylation was observed; which would be predicted to result in a decrease in gene expression, demonstrated that nearly 70 % of the genes affected belonged to five biological functional groups and some showed overlap. The genes altered are involved in transcription, RNA processing, translation, and trafficking of proteins. Additional genes are involved in pathways including the Notch, TGFβ, Wnt, MAPK, nuclear factor kappa B (NFkB), Fibroblast growth factor (FGF) and insulin-like growth factor (IGF) signaling. Other functions of genes that were altered included cellular adhesion, cell growth and survival, energy production, myogenic function, oxidative stress response, extracellular matrix proteins, and ubiquitin-mediated degradation.

As seen with the miRNA results, most of the genes that could be affected by a decrease in methylation in their promoter are not involved in any one particular biological function (Additional file 5: Table S5). A small number (<3 per category) of genes affected by decreased methylation contribute to general cellular functioning, actin proteins, proteins of the extracellular matrix, energy metabolism, cellular adhesion, cell growth, and neural functioning. These results suggest that decreased gene promoter methylation minimally contributes to accentuated SKM loss in CBA/SIV macaques.

## Discussion

We examined the SKM transcriptional and epigenetic changes, including differential expression of miRNA and promoter methylation profile, resulting from CBA administration to SIV-infected rhesus macaques. The results obtained were used to develop a gene regulatory network illustrating the principal sites of CBA-mediated alterations associated with SKM wasting at end-stage SIV infection. Our results show that CBA disrupts complex gene regulatory networks that affect the interplay between transcriptional and epigenetic factors leading to altered expression of genes whose biological functions contribute to one or more of the physiological events important for normal muscle functioning, including muscle regeneration to repair injury, muscle contraction and tensile strength, protein homeostasis, and NMJ function and development.

We and others previously focused on alcohol-mediated alterations in a single gene or class of genes involved in such biological functions of protein homeostasis [11, 18, 19], responses to increased oxidative stress [17], or immunological function [12]. Using new, more affordable genomics techniques and bioinformatics analytical methods, our previous, more limited SKM microarray analysis showed differential expression of genes-expression changes that were shown to be functionally involved in pro-inflammatory and ECM remodeling processes in SKM of CBA/SIV macaques [13]. In the present study, we expanded the analysis of this microarray data set to obtain a more comprehensive understanding of how CBA disrupts complex gene regulatory networks, thereby elucidating some additional underlying molecular mechanisms that contribute to CBA-dependent accentuation of SKM wasting at end-stage SIV infection.

**Fig. 3:**
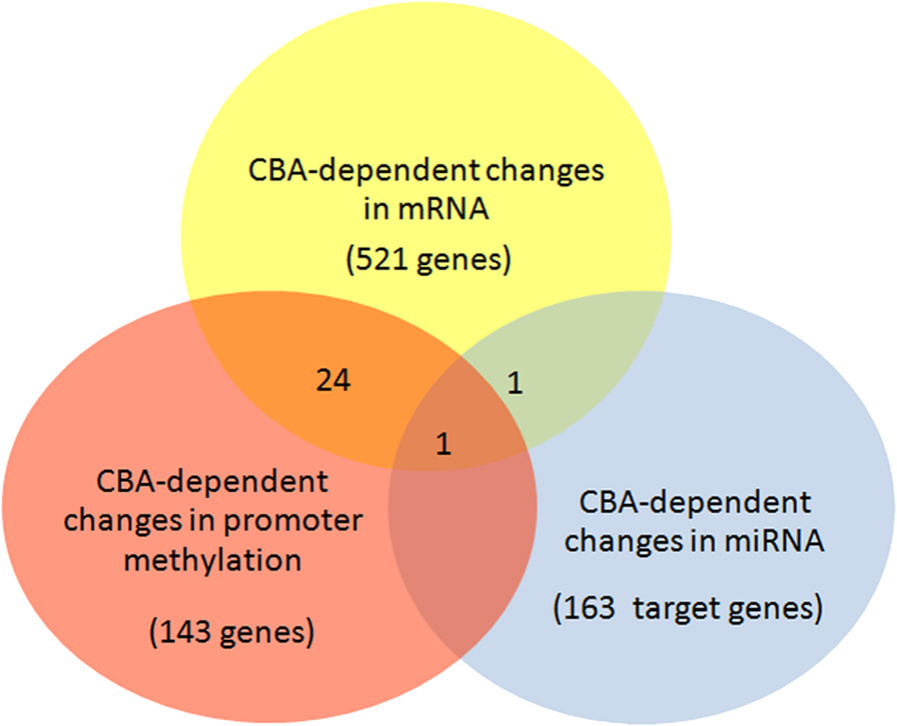
Venn diagram of CBA-dependent alterations in gene regulatory networks at end-stage SIV infection. The majority of differentially expressed genes (521) in the SKM of CBA/SIV macaques (≥1.5-fold different) compared to SKM of SUC/SIV macaques) did not correlate with alterations in miRNA or methylation. However, some of the differentially expressed genes were epigenetically regulated: changes in methylation status (24 genes), miRNA expression (1 gene), or both together (1 gene) were inversely proportional to the observed CBA-dependent changes in mRNA gene expression. Further, there were CBA-dependent miRNA (163 target genes) and methylation (143 genes) alterations that were not found in the mRNA data that potentially contribute to skeletal muscle wasting

Gene regulatory networks have both transcriptional and epigenetic components, including regulation of the initiation of gene expression through the action of transcription factors, the pre-transcriptional epigenetic regulation through promoter methylation, and the post-transcriptional regulation of mRNA stability or translation through the action of microRNAs (Fig. 3). Consistent with the interplay between these events, we identified several instances in which changes in methylation status (24 genes affected), miRNA expression (1 gene affected), or both (1 gene affected) are inversely proportional to our observed CBA-dependent changes in mRNA gene expression (Fig. 3). However, the majority of genes with expression changes ≥1.5-fold (as compared to expression in SUC/SIV macaques) did not correlate with alterations in miRNA or methylation. This suggests that the activation or repression of transcription initiation, that are independent of epigenetic modulation, are the most likely regulatory mechanisms responsible for these changes in gene expression. Further, a majority of genes that are affected by promoter methylation or that are validated/predicted targets of miRNA were not seen in our mRNA data, suggesting that these epigenetic changes have more subtle effects on the expression of individual genes (<1.5-fold change).

**Fig. 4:**
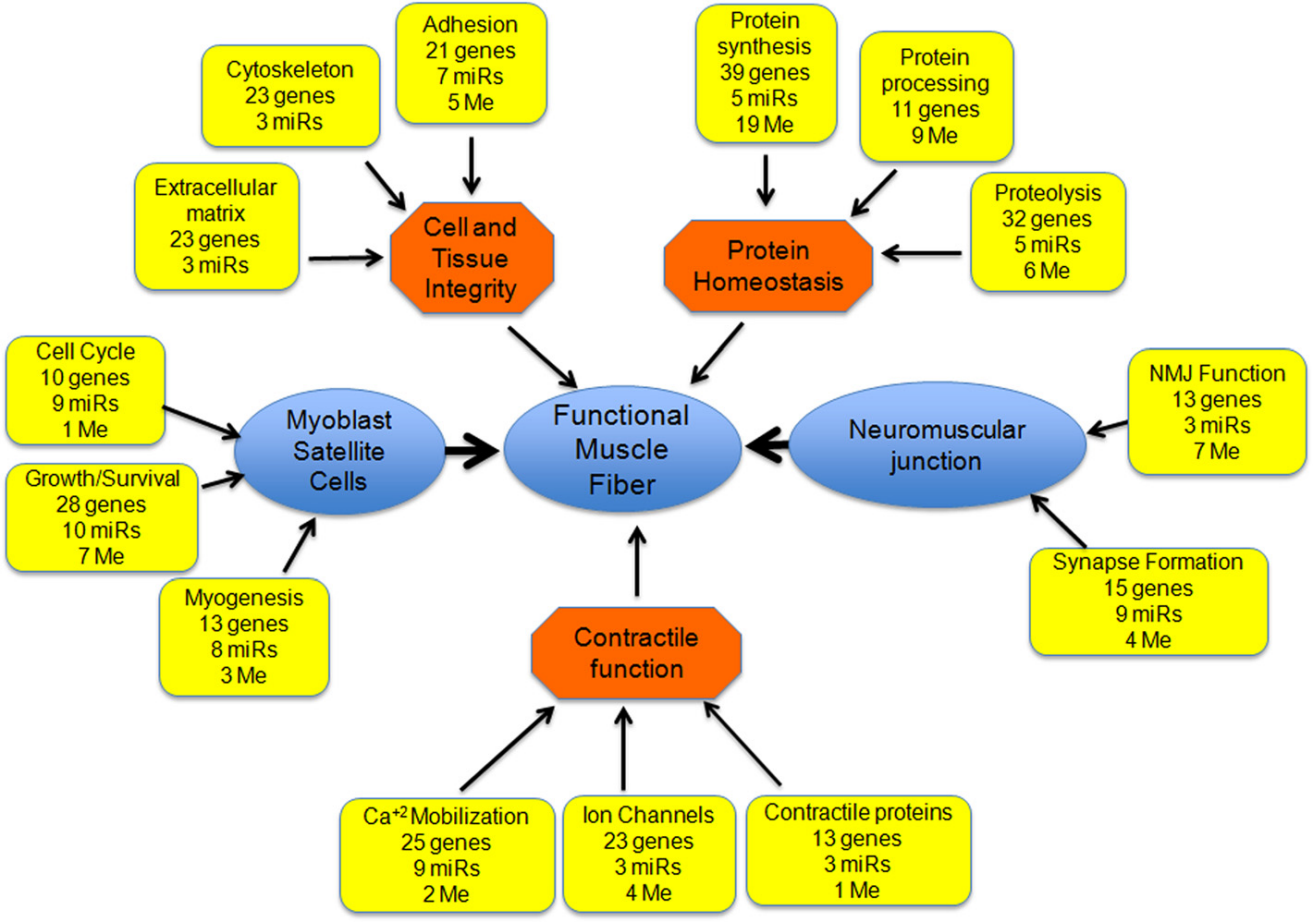
Schematic integrating the global effects of CBA on gene regulatory networks important for normal muscle function at end-stage SIV infection. Normal muscle function requires functional muscle fibers and the ability to activate satellite cells to proliferate and differentiate and pre-synaptic neurons to form functional neuromuscular junctions. The blue ovals indicate the cell type or physiological function necessary for normal muscle function and integrity. The orange octagons indicate the general biological functions required to maintain the function and/or integrity of the cell type or physiological function. The yellow boxes indicate the specific biological functions affected by CBA-dependent changes in gene regulatory networks. The number of mRNA (genes), microRNA (miRs), or methylation (Me) that are altered due to CBA are indicated for each specific biological function

The results from these studies identify four additional distinct biological categories in which gene regulatory networks are altered in a CBA-dependent manner: (1) “universal” cellular functions, (2) protein homeostasis, (3) calcium and ion homeostasis, which affect the functioning of muscle and neuromuscular junctions, and (4) satellite cell growth and survival (Fig. 4).

### “Universal” cellular functions

We found that 22 % of mRNAs, 18 % of target genes of altered miRNAs, and 21 % genes with promoters whose methylation status changed are involved with “universal” cellular functions. These universal functions include glycolysis and energy production, histone remodeling and modification, multifunctional transcriptional regulators, general kinases and phosphatases, lipid biosynthesis and degradation, multifunctional signaling pathways, maintaining DNA and chromosome integrity, transport of large molecules, and general mitochondrial function. Although seemingly diverse, and although each may not independently have a large biological impact, these changes combined as a whole would be expected to have a significant effect on the ability of any cell to function, regardless of the tissue of origin.

### Protein homeostasis

Previous findings from our laboratory have demonstrated that CBA may disrupt protein homeostasis through altered insulin signaling and increased ubiquitin-proteasome-dependent protein degradation [20], possibly inhibiting protein synthesis by altering mTOR signaling [21], and affecting translation elongation [22]. Consistent with these previous reports, we found changes in gene regulatory networks that affect all three of these biological functions. However, we also detected effects on gene regulatory networks whose functions contribute to additional aspects of protein homeostasis, including the biosynthesis of nucleotides and amino acids, the generation of charged tRNA, the formation of functional ribosomes, transcription, processing and editing of mRNA, oligosaccharide biosynthesis, and trafficking of proteins through the endoplasmic reticulum and trans-Golgi network. Thus, CBA impacts processes preceding transcription and culminating in the production of fully modified functional proteins or glycoproteins. Interestingly, although methylation changes influence genes with varied biological functions, nearly 25 % of genes are involved with protein homeostasis, suggesting that the largest impact of changes in methylation is on finer modulation of genes important for the production of functional proteins.

### Calcium and ion homeostasis

The increases in intracellular calcium stores, mediated through the mobilization and influx of calcium, are essential for many aspects of proper muscle functioning, including contraction, NMJ function, and differentiation. Further, sodium and potassium channels contribute to proper muscle and NMJ functioning by regulating muscle contractions and mobilizing intracellular calcium stores. The proper functioning of muscle fibers, along with the ability of satellite cells to fuse into functional muscle, requires the tight regulation of calcium influx, efflux, and intracellular calcium mobilization [23, 24]. Further, NMJ, the critical neuromuscular link mediating contraction, requires calcium for acetylcholine release [18]. Adding complexity to this process is the interplay between calcium and ion channels in which voltage-gated Na^+^/K^+^ channels are essential to mediate signals originating in the central nervous system to induce calcium release in muscle cells, thereby facilitating contraction. Our results show that CBA/SIV alters gene regulatory networks that affect genes important for calcium and ion homeostasis, either through direct expression, post-transcriptional regulation by miRNA, or altered methylation status. These results suggest that CBA may reduce the ability of NMJ to transmit signals to SKM fibers thereby affecting the mobilization of intracellular calcium stores, which would decrease the functionality of muscle contractions and the ability of muscle satellite cells to successfully fuse to form new tissue in response to muscle injury.

### Satellite cell growth and survival

Moderate everyday SKM use and injury trigger a cascade of events important for muscle repair [19]. As part of this response, the muscle stem cells (or myoblast satellite cells) proliferate, enter the myogenic program, and fuse with existing muscle fibers. Once expanded, satellite cells express and activate common myogenic factors, reorganize their cytoskeleton to mediate fusion, and interact with the ECM to not only repair the myofibers, but also to restore the interaction between myofibers and surrounding tissue environment. It was demonstrated that in the absence of satellite cells, there is excessive accumulation of ECM and this adversely affects the increase in muscle mass in response to muscle overload experiments [25]. Our results indicate that at end-stage SIV infection, CBA negatively alters gene regulatory networks that affect genes important for cell growth and survival, cytoskeleton reorganization or ECM involvement, and regulation of myogenesis. The expression of a number of genes is affected, which when combined over long periods of time, may significantly impact SKM regenerative capacity and contribute to SKM wasting.

The primary objective of the study was to identify a global network of the transcriptomic and epigenomic profiles from skeletal muscle derived from CBA/SIV whole animal studies. We were able to confirm the differential expression of 3 selected upregulated miRNAs and some of their target mRNAs by qPCR. ESR1, a target gene for 3 of the upregulated miRNAs (miR-34a, miR-18 and miR-20), was downregulated in the gene microarray, showed increased promoter methylation in the methylation array, and decreased mRNA expression as confirmed by qPCR in the SKM of CBA/SIV macaques. ESR1 is required for translocation of GLUT-4 receptors to the plasma membrane for glucose uptake. Its role in development of insulin resistance has been demonstrated in ESR1 knockout mice. ESR1 is also required for satellite cell proliferation [26–29]. Thus, decreased ESR1 expression in the SKM of CBA/SIV macaques potentially contributes to impaired glucose homeostasis and satellite cell growth. There was also a significant decrease in the expression of BCL-2, a target of miR-34a, in the SKM of CBA/SIV macaques. BCL-2 promotes cellular survival [30] and may be implicated in satellite cell growth and survival.

Taken together, our results allow us to propose a model describing the CBA-dependent global alterations in gene regulatory networks that contribute to muscle wasting in end-stage SIV infection. In this model, normal muscle function requires functional contractile muscle fibers, as well as the ability to activate fusion of myoblast satellite cells to regenerate injured muscle tissue and the ability of pre-synaptic neurons to form functional neuromuscular junctions (Fig. 4). The occurrence of each of these events depends on several subcategories of biological functions. For example, the proper contractile functioning of muscle cells requires cell and tissue integrity, proper protein homeostasis, and the presence of the mechanical components that drive contraction (Fig. 4, orange octagons). Each of these individual biological functions requires the finely balanced expression of specific genes, a balance regulated through transcriptional and epigenetic gene regulatory networks (Fig. 4, yellow boxes). Adding to this finely balanced regulatory network, many specific genes and miRNA contribute to multiple biological functions, such as calcium and ion homeostasis, which work in a finely coordinated manner to facilitate muscle contraction; NMJ function and myoblast satellite cell fusion; as well as cytoskeletal integrity and ECM maintenance, which contribute to muscle regeneration and muscle tissue integrity.

CBA administration promotes a persistent inflammatory SKM environment resulting from increased expression of a myriad of genes important for immunological function, inflammatory responses, and ability to combat oxidative stress (data not shown) [12, 13]. This persistent inflammatory state promotes changes in transcriptional and epigenetic regulation of hundreds of genes, either through direct effects on the initiation of transcription to alter the expression of genes and miRNA, the post-transcriptional miRNA regulation of mRNA stability and translation, or the altered methylation status of gene promoter regions (Fig. 3). These alterations produce subtle and significant effects on the expression of genes, which individually may not produce significant changes but when combined as a whole, work to disrupt the finely tuned balance required for normal muscle function and repair, thereby eliciting a gradual process of muscle wasting.

## Conclusion

Our results provide an integrated analysis of gene regulatory networks affecting SKM wasting in CBA/SIV macaques that extends our previous observations on ECM [13] and protein homeostasis [20, 31]. Alterations in calcium and ion homeostasis, NMJ functions, deficiencies in growth, survival, and regenerative capability of myoblasts, “universal” cellular functions, and gene regulatory networks expand the scope of CBA-mediated effects beyond those exclusive to maintaining protein homeostasis. Together, these alterations create a global, interconnected, and integrated network that leads to a general loss of SKM mass due to the inability of muscles to function, respond to, and repair damage and injury.

## Methods

SKM used for these studies was obtained from animals used in experiments approved by the Institutional Animal Care and Use Committee at both Tulane National Primate Research Center (TNPRC) in Covington, Louisiana and Louisiana State University Health Sciences Center (LSUHSC) in New Orleans, Louisiana, and adhered to National Institutes of Health guidelines for the care and use of experimental animals. The pathophysiological course of SIV infection has been previously described in published manuscripts [11–14]. A total of 28 4–6-year-old male macaques (*Macaca mulatta*) were studied in three experimental groups: SUC/SIV group (*n* = 9), CBA/SIV group (*n* = 11), and control group (*n* = 8). For the control group, skeletal muscle samples were obtained at necropsy from a group of SIV-negative, healthy control macaques and used as reference values for comparison of analyzed variables.

### Experimental protocol

The gastric catheter placement for alcohol delivery, the alcohol delivery protocol, and the route of SIV infection have been described in detail elsewhere [11, 14, 32]. Animals were briefly exposed to daily intragastric administration of a mean of 2.5 g per kg body weight ethanol (30 % w/v water), beginning 3 months prior to SIV infection and continuing throughout the duration of study. This protocol of CBA administration provided an average of 15 % of the animals’ total daily caloric intake and produced blood alcohol concentrations of 50–60 mM. Control animals were infused with sucrose. Animals were provided monkey chow (Lab Fiber Plus Primate diet DT; PMI Nutrition International, St. Louis, MO) *ad libitum* and supplemented with fruits, vitamins, and Noyes treats (Research Diets, New Brunswick, NJ).

Three months after initiating CBA administration, animals were inoculated intravenously with 10,000 times the 50 % infective dose (ID_50_) of SIV_mac251_. SIV disease progression was monitored throughout the study period through clinical, biochemical, and immunological parameters (CD4/CD8 lymphocyte ratios) in addition to plasma viral kinetics (SIV gag RNA levels) as described elsewhere [12, 32]. SKM (gastrocnemius) samples were obtained at necropsy when animals met any one of the criteria for euthanasia based on the following: (1) loss of 25 % of body weight from maximum body weight since assignment to protocol, (2) major organ failure or medical conditions unresponsive to treatment, (3) surgical complications unresponsive to immediate intervention, or (4) complete anorexia for 4 days. SKM tissue samples were dissected, snap frozen, and stored at −80 °C until analyses. SKM samples from all animals in the three treatment groups were used for the gene microarray, microRNA microarray, methylation array and qPCR. SKM samples used for the analysis in this study were used in two previously published studies [13, 14].

#### mRNA Microarray analysis

To determine how CBA affects the expression of mRNA at end-stage SIV infection, total RNA was isolated from SKM at necropsy from a total of 28 animals (SUC/SIV (*n* = 9), CBA/SIV (*n* = 11), and control (*n* = 8)) and microarray analysis was performed as previously described on all samples [13]. Briefly, the microarray hybridization was performed at the Stanley S. Scott Cancer Center’s Translational Genomics Core at LSUHSC in New Orleans, Louisiana. Total RNA was extracted using the RNeasy Mini Kit (Qiagen, Valencia, CA) according to the manufacturer's instructions. The RNA was hybridized to Illumina HumanWG6_v3 chips (San Diego, CA) following manufacturer’s instructions. For data analysis the samples were normalized using the cubic spline algorithm, assuming that the distribution of transcripts is similar [33]. Differential expression was determined by comparing treatment and control groups using the Illumina Custom algorithm that assumes that target signal intensity is normally distributed among replicates corresponding to some biological condition.

The fold change in gene expression of SUC/SIV and CBA/SIV was obtained by dividing the expression level of each gene by that of the control. The fold change of CBA/SIV/SUC/SIV was obtained by dividing the expression level of each gene between CBA/SIV and SUC/SIV. Comparisons between the gene expression levels of CBA/SIV with SUC/SIV animals reflect the impact of CBA on SIV-mediated changes in gene expression. Genes whose expression was altered in an alcohol-dependent manner by ≥1.5-fold were examined. The GEO accession number is GSE59111.

Gene Set Enrichment Analysis (GSEA) was run on the normalized, unfiltered microarray dataset as suggested in the tools implementation (http://software.broadinstitute.org/gsea/index.jsp) version 2.2.1) [34, 35] using the c5.all.v5.symbols. (Gene ontology), running 1000 permutations and excluding gene sets with fewer than 5 genes or more than 500. Using these parameters, 111 gene sets were selected for the analysis. The GSEA statistics are detailed in http://www.broadinstitute.org/gsea/doc/GSEAUserGuideFrame.html [36].

#### MicroRNA microarray analysis

To determine the impact of CBA on miRNA expression at end-stage SIV infection, small RNA (<30 bp) were purified. The microRNA microarray hybridization was performed at the microarray core at LSUHSC. Briefly, 500 μg of total RNA was biotin-labeled using the FlashTag Biotin HSR kit (Genisphere, Hatfield, PA) according to the manufacturer’s instructions. All samples showed expected labeling and the resulting targets were hybridized to miR 2.0 arrays (Affymetrix) containing 15,644 microRNA probe sets from the miRBASE v15 (http://microrna.sanger.ac.uk), which recognize microRNAs from a number of organisms including human and rhesus macaques. The arrays were washed and processed on a Fluidics Station 450 and scanned with a confocal laser scanner (GeneChip Scanner 3000, Affymetrix) according to the manufacturer's instructions. Data from the microarrays were analyzed with Affymetrix microRNA QC Tool according to the manufacturer’s instructions. Differential miR expression was performed using the One-Way ANOVA workflow with multiple test correction (FDR = 0.05) and selected from volcano plot using p-value <0.05 and exhibiting a ≥ 1.5-fold change cutoff.

All differentially regulated miRs were analyzed using miRSystem (http://mirsystem.cgm.ntu.edu.tw/) [37]. miRSystem is a database which integrates seven miRNA target gene prediction programs: DIANA, miRanda, miRBridge, PicTar, PITA, rna22, and TargetScan. The database also contains validated data from TarBase and miRecords. To balance the reliability of the predictions, three algorithm hits were used: 1) hypergeometric p-value determination; 2) empirical p-value is determined by ranking the enriched hypergeometric probability as compared with null baseline probabilities. The null baseline probability was established by randomly selecting a group of miRNAs, between 1 and 100, and using the default values in mirsystem to calculate the raw p-value for each pathway [37]; and 3) a weighted pathway-ranking method is also calculated from the expression ratios of the differentially regulated miRNAs to rank the enriched pathways. For each functional category, the ranking score was obtained by summation of the weight of its miRNA times its enrichment -log (p-value) from the predicted target genes $\left\{\mathrm{Score}={\displaystyle \sum \mathrm{AmiRNAwi}\left[\hbox{-} \log 10\left(\mathrm{pi}\right)\right]}\right\}$. The target genes, pathway ranking, and functional annotation summaries are included in the results. The parameters set for analysis were: a) validated genes equal to or more than 3, b) observed to expected ratio (O/E) greater or equal to 2 and c) total genes in the pathway ≥ 25 and ≤ 500.

We also analyzed individual differentially expressed miRNAs using miRTarBase and TargetScan. A search for target genes using miRTarBase [38] determined that 4 of the downregulated miRNA target 12 independent genes; targeting is validated in the literature by at least two independent experimental methods [38]. For the remaining miRNAs that have no experimentally validated gene targets, we used the TargetScan database, which identifies genes statistically predicted to be targets, using Homo sapiens as the reference. This search identified 8 individual downregulated miRNAs predicted to target 18 independent genes based on their predicted efficacy of targeting (context score) [39, 40] or probability of conserved targeting (P_CT_) [41] [context score ≥ 85 %; P_CT_ ≥ 0.8] [42].

### DNA methylation microarray analysis

To determine how CBA affects promoter methylation in SIV-infection, the Infinium HumanMethylation27 array was utilized. The Infinium HumanMethylation 27 examines more than 27,000 CpG islands in more than 14,000 genes’ promoters. SKM samples were bisulphite-converted with Zymo EZ DNA Methylation kit (Zymo Research, Irvine, CA, USA). GenomeStudio v2011.1 (Illumina, San Diego, CA, USA) with Methylation module 1.9.0 software was used in the methylation analysis. The Infinium platform assays covers 96 % of CpG islands with multiple sites in the island, the shores (within 2 kb from CpG islands), and the shelves (>2 kb from CpG islands). All the Illumina quality controls were acceptable, including sample-independent and dependent controls, staining controls, extension controls, target removal controls, hybridization controls, bisulphite conversion I and II controls, specificity controls, non-polymorphic controls and negative controls. Probes were considered to be differentially methylated if the resulting adjusted p-value was <0.05. The Benjamini-Hochberg method [43] was used to adjust the p-values and ensure that the false discovery rate was <0.05. The corresponding gene list was derived from the gene annotations associated with the probes. The GEO accession number is GSE75729. Functional enrichment analyses of genes with differential methylation of promoter regions were performed using DAVID Bioinformatics Resources. The gene ontology Biological Processes (BP_all) is represented for genes. The count: number of genes involved in the term; %: percentage of involved genes/total genes; p-value: modified fisher exact p-value, EASE Score (*p* ≤ 0.05); Benjamini: adjusted p-value using Benjamini-Hochberg procedure is presented in the results [44, 45].

The biological functions of individual differentially expressed genes in the mRNA microarray, validated target genes of differentially expressed miRNAs and genes altered in the DNA methylation array were also categorized by searching each individual gene in the GeneCards® Human Genome Database (http://www.genecards.org). This is an integrative database that provides descriptions of gene functions as extracted from multiple public databases, including Entrez Gene, UniProtKB, Tocris, Bioscience, and PharmGKB. Additional information was derived from searching each individual gene in the NCBI Gene Database (http://www.ncbi.nlm.nih.gov/gene/); the remaining genes with unclear biological function were analyzed using the DAVID Bioinformatics Database (https://david.ncifcrf.gov). This allowed for analysis of genes with specific functions relevant to skeletal muscle function. Those genes that did not fall within any obvious biological function after these analyses were classified as “Miscellaneous.” This analysis is included as supplementary tables.

#### q PCR (qPCR) for miR expression

To validate the deep sequencing data, the relative expression of 3 differentially expressed miRs (miR-34a, miR-10b, miR-20) was further determined by individual Taqman miR assays (Thermo Fisher Scientific). Approximately 200–250 ng of total RNA was reverse-transcribed using the stem loop primers provided in the predesigned kit and ~1.3 μl of cDNA was subjected to 40 cycles of PCR on the CFX96 Bio-Rad PCR cycler (Bio-Rad) using the following thermal cycling conditions: 50 °C for 2 min, 95 °C for 10 min followed by 40 repetitive cycles of 95 °C for 15 s and 60 °C for 1 min. As a normalization control for RNA loading, SNOU6 were amplified in duplicate wells on the same multi-well plate.

#### qPCR for target genes of differentially regulated miRNAs

Total RNA isolated for miR sequencing studies was used for gene expression analysis as well. cDNA was synthesized from 1000 ng of the resulting total RNA using the Quantitect Reverse Transcriptase Kit (Qiagen), in accordance with the manufacturer’s instructions. Primers were designed to span exon-exon junctions (IDT, Coralville, IA) and used at a concentration of 500 nmol. The final reactions were made to a total volume of 20 μl with Quantitect SyBr Green PCR kit (Qiagen). All reactions were carried out in duplicate on a CFX96 system (Bio-Rad Laboratories, Hercules, CA) for qPCR detection. qPCR data were analyzed using the comparative Ct (delta-delta-Ct, ΔΔCT) method. Target genes were compared with the endogenous control (ribosomal protein S13 (RPS13)) and CBA/SIV and SUC/SIV values were normalized to controls.

### Availability of supporting data

The data sets supporting the results of this article are available in Gene Expression Omnibus (GEO). The GEO accession numbers for the microarray data are GSE59111 and GSE75729. The links to the data are http://www.ncbi.nlm.nih.gov/geo/query/acc.cgi?acc=GSE75729 and http://www.ncbi.nlm.nih.gov/geo/query/acc.cgi?acc=GSE59111.

## Additional files

Additional file 1: Table S1. Functional enrichment of CBA-dependent alterations in mRNA expression at end-stage SIV infection. (DOCX 37 kb) Additional file 2: Table S2. CBA-dependent alterations in mRNA expression at end-stage SIV infection. (DOCX 48 kb) Additional file 3: Table S3. Functional annotation summary and pathway summary of predicted target genes of CBA-altered microRNAs at end stage SIV infection. (DOCX 73 kb) Additional file 4: Table S4. CBA-dependent alterations in microRNA expression at end-stage SIV infection. (DOCX 24 kb) Additional file 5: Table S5. CBA-dependent alterations in promoter methylation at end-stage SIV infection. (DOCX 20 kb)

## Abbreviations

PLWHA: people living with HIV/AIDS
AUD: alcohol use disorders
SIV: simian immunodeficiency virus
CBA: chronic binge alcohol
SKM: skeletal muscle
SUC: sucrose
HIV: human immunodeficiency virus
AIDS: acquired immunodeficiency syndrome
ART: antiretroviral therapy
FDR: false discovery rate
ECM: extracellular matrix
NMJ: neuromuscular junction
MEF2C: myogenic enhancing factor 2C
PNN: pinin desmosome associated protein
TMEM119: transmembrane protein 119
ESR1: estrogen receptor-alpha
BCl-2: b-cell Lymphoma-2
KRAS: kirsten rat sarcoma
MAPK: mitogen activated protein kinase
TGF β: transforming growth factor
ID50: 50 % infective dose ()
NFkB: nuclear factor kappa B
FGF: fibroblast growth factor
IGF: insulin-like growth factor
GSEA: gene set enrichment analysis
ANOVA: analysis of variance
O/E: observed to expected ratio
PCT: probability of conserved targeting
BP: biological processes
ΔΔCT: delta-delta-Ct
RPS13: Ribosomal protein S13
