# Supplementary material for: Chronic binge alcohol administration dysregulates global regulatory gene networks associated with skeletal muscle wasting in simian immunodeficiency virus-infected macaques
Source: BMC Genomics. 2015 Dec 23;16:1097. doi: 10.1186/s12864-015-2329-z (PMC4690320; doi:10.1186/s12864-015-2329-z)
Supplement: Additional file 1: Table S1. — Functional enrichment of CBA-dependent alterations in mRNA expression at end-stage SIV infection. (DOCX 37 kb) [file 12864_2015_2329_MOESM1_ESM.docx]

**Additional file 1: Table S1: Functional enrichment of CBA-dependent alterations in mRNA expression at end-stage SIV infection**

| Name | **Size** | **ES** | **NES** | **Nom p-val** | **Fdr q-val** | **Fwer p-val** | **Rank at max** |
| --- | --- | --- | --- | --- | --- | --- | --- |
| **UPREGULATED in CBA/SIV** |  |  |  |  |  |  |  |
| Regulation_of_transcription_from_rna_polymerase_ii_  promoter | 19 | 0.47 | 1.97 | 0.00 | 0.25 | 0.17 | 238 |
| Intracellular_organelle_part | 38 | 0.34 | 1.88 | 0.01 | 0.25 | 0.31 | 134 |
| Transcription_from_rna_polymerase_ii_promoter | 26 | 0.39 | 1.82 | 0.01 | 0.24 | 0.41 | 298 |
| Intracellular_signaling_cascade | 29 | 0.36 | 1.78 | 0.01 | 0.23 | 0.49 | 183 |
| Organelle_part | 39 | 0.32 | 1.78 | 0.01 | 0.19 | 0.51 | 134 |
| Transcription_factor_binding | 20 | 0.41 | 1.77 | 0.02 | 0.17 | 0.52 | 288 |
| Transcription | 36 | 0.32 | 1.74 | 0.01 | 0.18 | 0.60 | 298 |
| Negative_regulation_of_biological_process | 41 | 0.32 | 1.73 | 0.01 | 0.17 | 0.62 | 309 |
| Rna_biosynthetic_process | 33 | 0.33 | 1.71 | 0.02 | 0.16 | 0.66 | 279 |
| Regulation_of_cell_cycle | 16 | 0.44 | 1.71 | 0.02 | 0.15 | 0.66 | 117 |
| Programmed_cell_death | 25 | 0.36 | 1.70 | 0.03 | 0.14 | 0.68 | 297 |
| Transcription_dna_dependent | 33 | 0.33 | 1.70 | 0.02 | 0.13 | 0.68 | 279 |
| Cell_cycle_go_0007049 | 20 | 0.39 | 1.70 | 0.03 | 0.12 | 0.69 | 117 |
| Response_to_stress | 31 | 0.34 | 1.69 | 0.03 | 0.12 | 0.70 | 300 |
| Apoptosis_go | 25 | 0.36 | 1.69 | 0.04 | 0.11 | 0.71 | 297 |
| Rna_metabolic_process | 37 | 0.31 | 1.68 | 0.02 | 0.11 | 0.72 | 279 |
| Nucleotide_and_nucleic_acid_metabolic_process | 52 | 0.28 | 1.67 | 0.02 | 0.11 | 0.74 | 298 |
| Regulation_of_transcription | 30 | 0.33 | 1.67 | 0.03 | 0.11 | 0.75 | 288 |
| Regulation_of_transcriptiondna_dependent | 27 | 0.35 | 1.67 | 0.03 | 0.10 | 0.75 | 277 |
| Regulation_of_rna_metabolic_process | 27 | 0.35 | 1.66 | 0.02 | 0.10 | 0.76 | 277 |
| Negative_regulation_of_cellular_process | 38 | 0.30 | 1.64 | 0.02 | 0.11 | 0.82 | 309 |
| Cell_development | 30 | 0.32 | 1.64 | 0.01 | 0.10 | 0.82 | 297 |
| Protein_kinase_cascade | 25 | 0.35 | 1.63 | 0.03 | 0.10 | 0.82 | 183 |
| Nucleus | 58 | 0.25 | 1.57 | 0.03 | 0.13 | 0.91 | 288 |
| Regulation_of_nucleobasenucleosidenucleotide_and_  nucleic_acid_metabolic_process | 31 | 0.31 | 1.56 | 0.04 | 0.14 | 0.92 | 288 |
| Biopolymer_metabolic_process | 79 | 0.23 | 1.56 | 0.03 | 0.13 | 0.92 | 279 |
| Response_to_chemical_stimulus | 24 | 0.32 | 1.53 | 0.06 | 0.15 | 0.95 | 182 |
| Signal_transduction | 69 | 0.24 | 1.51 | 0.04 | 0.16 | 0.96 | 300 |
| Dna_binding | 29 | 0.31 | 1.50 | 0.08 | 0.17 | 0.97 | 279 |
| Regulation_of_gene_expression | 34 | 0.29 | 1.48 | 0.07 | 0.17 | 0.98 | 288 |
| Positive_regulation_of_biological_process | 34 | 0.28 | 1.45 | 0.07 | 0.19 | 0.99 | 309 |
| Defense_response | 17 | 0.36 | 1.45 | 0.09 | 0.19 | 0.99 | 260 |
| Regulation_of_developmental_process | 23 | 0.31 | 1.40 | 0.11 | 0.23 | 1.00 | 297 |
| Purine_nucleotide_binding | 16 | 0.34 | 1.40 | 0.13 | 0.22 | 1.00 | 148 |
| Regulation_of_metabolic_process | 41 | 0.25 | 1.36 | 0.10 | 0.25 | 1.00 | 288 |
| Regulation_of_cellular_metabolic_process | 41 | 0.25 | 1.36 | 0.12 | 0.25 | 1.00 | 288 |
| Purine_ribonucleotide_binding | 16 | 0.34 | 1.36 | 0.13 | 0.24 | 1.00 | 148 |
| Nucleotide_binding | 16 | 0.34 | 1.34 | 0.14 | 0.26 | 1.00 | 148 |
| Positive_regulation_of_cellular_process | 33 | 0.25 | 1.32 | 0.15 | 0.27 | 1.00 | 327 |
| Response_to_external_stimulus | 24 | 0.28 | 1.31 | 0.15 | 0.27 | 1.00 | 363 |
| Establishment_of_cellular_localization | 15 | 0.33 | 1.31 | 0.16 | 0.27 | 1.00 | 182 |
| Intracellular_non_membrane_bound_organelle | 24 | 0.27 | 1.29 | 0.18 | 0.29 | 1.00 | 134 |
| Non_membrane_bound_organelle | 24 | 0.27 | 1.28 | 0.17 | 0.30 | 1.00 | 134 |
| Cellular_localization | 16 | 0.31 | 1.24 | 0.22 | 0.33 | 1.00 | 182 |
| Regulation_of_apoptosis | 20 | 0.28 | 1.22 | 0.22 | 0.35 | 1.00 | 327 |
| Regulation_of_programmed_cell_death | 20 | 0.28 | 1.22 | 0.23 | 0.35 | 1.00 | 327 |
| Cytoplasm | 81 | 0.18 | 1.21 | 0.18 | 0.36 | 1.00 | 213 |
| Transcription_factor_activity | 17 | 0.29 | 1.20 | 0.26 | 0.36 | 1.00 | 367 |
| Immune_system_process | 23 | 0.25 | 1.14 | 0.30 | 0.44 | 1.00 | 313 |
| Receptor_binding | 22 | 0.24 | 1.09 | 0.35 | 0.50 | 1.00 | 251 |
| Nuclear_part | 21 | 0.25 | 1.09 | 0.36 | 0.50 | 1.00 | 134 |
| Receptor_activity | 25 | 0.22 | 1.05 | 0.38 | 0.55 | 1.00 | 151 |
| Regulation_of_transferase_activity | 16 | 0.25 | 1.03 | 0.43 | 0.57 | 1.00 | 147 |
| Regulation_of_catalytic_activity | 16 | 0.25 | 1.02 | 0.41 | 0.58 | 1.00 | 147 |
| Multicellular_organismal_development | 50 | 0.17 | 1.02 | 0.42 | 0.57 | 1.00 | 336 |
| Cell_proliferation_go_0008283 | 28 | 0.20 | 1.01 | 0.44 | 0.58 | 1.00 | 309 |
| Regulation_of_kinase_activity | 16 | 0.25 | 1.00 | 0.45 | 0.58 | 1.00 | 147 |
| Regulation_of_protein_kinase_activity | 16 | 0.25 | 1.00 | 0.47 | 0.57 | 1.00 | 147 |
| Transport | 41 | 0.18 | 0.97 | 0.48 | 0.61 | 1.00 | 182 |
| Regulation_of_biological_quality | 22 | 0.21 | 0.94 | 0.53 | 0.65 | 1.00 | 294 |
| Organ_development | 31 | 0.19 | 0.94 | 0.53 | 0.64 | 1.00 | 361 |
| Membrane_fraction | 16 | 0.23 | 0.91 | 0.56 | 0.68 | 1.00 | 46 |
| Extracellular_region_part | 19 | 0.21 | 0.91 | 0.55 | 0.68 | 1.00 | 297 |
| Cell_surface_receptor_linked_signal_transduction | 17 | 0.22 | 0.91 | 0.56 | 0.66 | 1.00 | 118 |
| Immune_response | 15 | 0.23 | 0.91 | 0.55 | 0.66 | 1.00 | 313 |
| Homeostatic_process | 16 | 0.21 | 0.88 | 0.61 | 0.70 | 1.00 | 294 |
| Anatomical_structure_morphogenesis | 22 | 0.19 | 0.86 | 0.61 | 0.71 | 1.00 | 248 |
| System_development | 42 | 0.15 | 0.84 | 0.67 | 0.73 | 1.00 | 248 |
| Regulation_of_cell_proliferation | 16 | 0.20 | 0.80 | 0.69 | 0.78 | 1.00 | 251 |
| Regulation_of_molecular_function | 19 | 0.18 | 0.79 | 0.72 | 0.79 | 1.00 | 147 |
| Anatomical_structure_development | 52 | 0.13 | 0.78 | 0.80 | 0.78 | 1.00 | 31 |
| Plasma_membrane | 62 | 0.11 | 0.68 | 0.89 | 0.90 | 1.00 | 205 |
| Nervous_system_development | 18 | 0.16 | 0.64 | 0.86 | 0.92 | 1.00 | 31 |
| Biopolymer_modification | 38 | 0.11 | 0.60 | 0.95 | 0.94 | 1.00 | 278 |
| **UPREGULATED in SUC/SIV** |  |  |  |  |  |  |  |
| Macromolecular_complex | 43 | -0.20 | -1.10 | 0.31 | 0.77 | 1.00 | 234 |
| Intrinsic_to_membrane | 52 | -0.19 | -1.10 | 0.33 | 0.82 | 1.00 | 247 |
| Protein_complex | 39 | -0.20 | -1.06 | 0.39 | 0.82 | 1.00 | 234 |
| Neurological_system_process | 16 | -0.28 | -1.11 | 0.32 | 0.84 | 1.00 | 86 |
| Organelle_organization_and_biogenesis | 20 | -0.22 | -0.96 | 0.54 | 0.85 | 1.00 | 119 |
| Intrinsic_to_plasma_membrane | 38 | -0.17 | -0.94 | 0.53 | 0.86 | 1.00 | 247 |
| Integral_to_plasma_membrane | 37 | -0.18 | -0.97 | 0.51 | 0.87 | 1.00 | 247 |
| Cellular_protein_metabolic_process | 57 | -0.15 | -0.92 | 0.57 | 0.87 | 1.00 | 241 |
| Plasma_membrane_part | 46 | -0.19 | -1.12 | 0.29 | 0.89 | 1.00 | 247 |
| Cellular_macromolecule_metabolic_process | 58 | -0.16 | -0.98 | 0.48 | 0.89 | 1.00 | 241 |
| Membrane | 78 | -0.13 | -0.89 | 0.64 | 0.89 | 1.00 | 234 |
| Establishment_of_localization | 44 | -0.18 | -0.98 | 0.49 | 0.93 | 1.00 | 234 |
| Transmembrane_transporter_activity | 25 | -0.24 | -1.12 | 0.32 | 0.95 | 1.00 | 301 |
| Macromolecule_biosynthetic_process | 16 | -0.15 | -0.57 | 0.95 | 0.95 | 1.00 | 225 |
| Transferase_activity_transferring_  phosphorus_containing_groups | 31 | -0.20 | -0.99 | 0.45 | 0.96 | 1.00 | 248 |
| Transmembrane_receptor_activity | 16 | -0.15 | -0.58 | 0.96 | 0.98 | 1.00 | 234 |
| Cell_cell_signaling | 21 | -0.15 | -0.67 | 0.88 | 0.99 | 1.00 | 711 |
| Phosphorylation | 26 | -0.15 | -0.72 | 0.84 | 0.99 | 1.00 | 210 |
| Protein_modification_process | 37 | -0.11 | -0.60 | 0.95 | 0.99 | 1.00 | 219 |
| Membrane_part | 61 | -0.22 | -1.37 | 0.09 | 1.00 | 1.00 | 234 |
| Cytoplasmic_part | 50 | -0.24 | -1.39 | 0.10 | 1.00 | 1.00 | 183 |
| Protein_serine_threonine_kinase_activity | 19 | -0.31 | -1.28 | 0.19 | 1.00 | 1.00 | 248 |
| Substrate_specific_transmembrane_  transporter_activity | 24 | -0.27 | -1.22 | 0.21 | 1.00 | 1.00 | 301 |
| Protein_kinase_activity | 22 | -0.28 | -1.24 | 0.21 | 1.00 | 1.00 | 248 |
| System_process | 25 | -0.26 | -1.20 | 0.22 | 1.00 | 1.00 | 301 |
| Ion_transmembrane_transporter_activity | 17 | -0.30 | -1.23 | 0.23 | 1.00 | 1.00 | 301 |
| Substrate_specific_transporter_activity | 25 | -0.25 | -1.18 | 0.26 | 1.00 | 1.00 | 301 |
| Kinase_activity | 27 | -0.24 | -1.15 | 0.28 | 1.00 | 1.00 | 248 |
| Phosphotransferase_activity_alcohol_group_as_acceptor | 24 | -0.25 | -1.13 | 0.30 | 1.00 | 1.00 | 248 |
| Integral_to_membrane | 51 | -0.19 | -1.14 | 0.32 | 1.00 | 1.00 | 247 |
| Cell_fraction | 20 | -0.19 | -0.80 | 0.72 | 1.00 | 1.00 | 247 |
| Extracellular_region | 28 | -0.16 | -0.78 | 0.72 | 1.00 | 1.00 | 704 |
| Protein_amino_acid_phosphorylation | 24 | -0.17 | -0.75 | 0.79 | 1.00 | 1.00 | 241 |
| Protein_metabolic_process | 61 | -0.12 | -0.75 | 0.82 | 1.00 | 1.00 | 241 |
| Post_translational_protein_modification | 32 | -0.15 | -0.72 | 0.83 | 1.00 | 1.00 | 182 |
| Biosynthetic_process | 28 | -0.14 | -0.67 | 0.88 | 1.00 | 1.00 | 278 |
| Cellular_biosynthetic_process | 21 | -0.14 | -0.62 | 0.90 | 1.00 | 1.00 | 278 |
| Regulation of_transcription_from_rna polymerase_ii_promoter | 19 | 0.47 | 1.97 | 0.00 | 0.25 | 0.17 | 238 |
| Intracellular_organelle_part | 38 | 0.34 | 1.88 | 0.01 | 0.25 | 0.31 | 134 |
| Transcription_from_rna_polymerase_ii_promoter | 26 | 0.39 | 1.82 | 0.01 | 0.24 | 0.41 | 298 |
| Intracellular_signaling_cascade | 29 | 0.36 | 1.78 | 0.01 | 0.23 | 0.49 | 183 |
| Organelle_part | 39 | 0.32 | 1.78 | 0.01 | 0.19 | 0.51 | 134 |
| Transcription_factor_binding | 20 | 0.41 | 1.77 | 0.02 | 0.17 | 0.52 | 288 |
| Transcription | 36 | 0.32 | 1.74 | 0.01 | 0.18 | 0.60 | 298 |
| Negative_regulation_of_biological_process | 41 | 0.32 | 1.73 | 0.01 | 0.17 | 0.62 | 309 |
| Rna_biosynthetic_process | 33 | 0.33 | 1.71 | 0.02 | 0.16 | 0.66 | 279 |
| Regulation_of_cell_cycle | 16 | 0.44 | 1.71 | 0.02 | 0.15 | 0.66 | 117 |
| Programmed_cell_death | 25 | 0.36 | 1.70 | 0.03 | 0.14 | 0.68 | 297 |
| Transcription_dna_dependent | 33 | 0.33 | 1.70 | 0.02 | 0.13 | 0.68 | 279 |
| Cell_cycle_go_0007049 | 20 | 0.39 | 1.70 | 0.03 | 0.12 | 0.69 | 117 |
| Response_to_stress | 31 | 0.34 | 1.69 | 0.03 | 0.12 | 0.70 | 300 |
| Apoptosis_go | 25 | 0.36 | 1.69 | 0.04 | 0.11 | 0.71 | 297 |
| Rna_metabolic_process | 37 | 0.31 | 1.68 | 0.02 | 0.11 | 0.72 | 279 |
| Nucleobasenucleosidenucleotide_and_nucleic_acid_  metabolic_process | 52 | 0.28 | 1.67 | 0.02 | 0.11 | 0.74 | 298 |
| Regulation_of_transcription | 30 | 0.33 | 1.67 | 0.03 | 0.11 | 0.75 | 288 |
| Regulation_of_transcriptiondna_dependent | 27 | 0.35 | 1.67 | 0.03 | 0.10 | 0.75 | 277 |
| Regulation_of_rna_metabolic_process | 27 | 0.35 | 1.66 | 0.02 | 0.10 | 0.76 | 277 |
| Negative_regulation_of_cellular_process | 38 | 0.30 | 1.64 | 0.02 | 0.11 | 0.82 | 309 |
| Cell_development | 30 | 0.32 | 1.64 | 0.01 | 0.10 | 0.82 | 297 |
| Protein_kinase_cascade | 25 | 0.35 | 1.63 | 0.03 | 0.10 | 0.82 | 183 |
| Nucleus | 58 | 0.25 | 1.57 | 0.03 | 0.13 | 0.91 | 288 |
| Regulation_of_nucleobasenucleosidenucleotide_and_  nucleic_acid_metabolic_process | 31 | 0.31 | 1.56 | 0.04 | 0.14 | 0.92 | 288 |
| Biopolymer_metabolic_process | 79 | 0.23 | 1.56 | 0.03 | 0.13 | 0.92 | 279 |
| Response_to_chemical_stimulus | 24 | 0.32 | 1.53 | 0.06 | 0.15 | 0.95 | 182 |
| Signal_transduction | 69 | 0.24 | 1.51 | 0.04 | 0.16 | 0.96 | 300 |
| Dna_binding | 29 | 0.31 | 1.50 | 0.08 | 0.17 | 0.97 | 279 |
| Regulation_of_gene_expression | 34 | 0.29 | 1.48 | 0.07 | 0.17 | 0.98 | 288 |
| Positive_regulation_of_biological_process | 34 | 0.28 | 1.45 | 0.07 | 0.19 | 0.99 | 309 |
| Defense_response | 17 | 0.36 | 1.45 | 0.09 | 0.19 | 0.99 | 260 |
| Regulation_of_developmental_process | 23 | 0.31 | 1.40 | 0.11 | 0.23 | 1.00 | 297 |
| Purine_nucleotide_binding | 16 | 0.34 | 1.40 | 0.13 | 0.22 | 1.00 | 148 |
| Regulation_of_metabolic_process | 41 | 0.25 | 1.36 | 0.10 | 0.25 | 1.00 | 288 |
| Regulation_of_cellular_metabolic_process | 41 | 0.25 | 1.36 | 0.12 | 0.25 | 1.00 | 288 |
| Purine_ribonucleotide_binding | 16 | 0.34 | 1.36 | 0.13 | 0.24 | 1.00 | 148 |
| Nucleotide_binding | 16 | 0.34 | 1.34 | 0.14 | 0.26 | 1.00 | 148 |
| Positive_regulation_of_cellular_process | 33 | 0.25 | 1.32 | 0.15 | 0.27 | 1.00 | 327 |
| Response_to_external_stimulus | 24 | 0.28 | 1.31 | 0.15 | 0.27 | 1.00 | 363 |
| Establishment_of_cellular_localization | 15 | 0.33 | 1.31 | 0.16 | 0.27 | 1.00 | 182 |
| Intracellular_non_membrane_bound_organelle | 24 | 0.27 | 1.29 | 0.18 | 0.29 | 1.00 | 134 |
| Non_membrane_bound_organelle | 24 | 0.27 | 1.28 | 0.17 | 0.30 | 1.00 | 134 |
| Cellular_localization | 16 | 0.31 | 1.24 | 0.22 | 0.33 | 1.00 | 182 |
| Regulation_of_apoptosis | 20 | 0.28 | 1.22 | 0.22 | 0.35 | 1.00 | 327 |
| Regulation_of_programmed_cell_death | 20 | 0.28 | 1.22 | 0.23 | 0.35 | 1.00 | 327 |
| Cytoplasm | 81 | 0.18 | 1.21 | 0.18 | 0.36 | 1.00 | 213 |
| Transcription_factor_activity | 17 | 0.29 | 1.20 | 0.26 | 0.36 | 1.00 | 367 |
| Immune_system_process | 23 | 0.25 | 1.14 | 0.30 | 0.44 | 1.00 | 313 |
| Receptor_binding | 22 | 0.24 | 1.09 | 0.35 | 0.50 | 1.00 | 251 |
| Nuclear_part | 21 | 0.25 | 1.09 | 0.36 | 0.50 | 1.00 | 134 |
| Receptor_activity | 25 | 0.22 | 1.05 | 0.38 | 0.55 | 1.00 | 151 |
| Regulation_of_transferase_activity | 16 | 0.25 | 1.03 | 0.43 | 0.57 | 1.00 | 147 |
| Regulation_of_catalytic_activity | 16 | 0.25 | 1.02 | 0.41 | 0.58 | 1.00 | 147 |
| Multicellular_organismal_development | 50 | 0.17 | 1.02 | 0.42 | 0.57 | 1.00 | 336 |
| Cell_proliferation_go_0008283 | 28 | 0.20 | 1.01 | 0.44 | 0.58 | 1.00 | 309 |
| Regulation_of_kinase_activity | 16 | 0.25 | 1.00 | 0.45 | 0.58 | 1.00 | 147 |
| Regulation_of_protein_kinase_activity | 16 | 0.25 | 1.00 | 0.47 | 0.57 | 1.00 | 147 |
| Transport | 41 | 0.18 | 0.97 | 0.48 | 0.61 | 1.00 | 182 |
| Regulation_of_biological_quality | 22 | 0.21 | 0.94 | 0.53 | 0.65 | 1.00 | 294 |
| Organ_development | 31 | 0.19 | 0.94 | 0.53 | 0.64 | 1.00 | 361 |
| Membrane_fraction | 16 | 0.23 | 0.91 | 0.56 | 0.68 | 1.00 | 46 |
| Extracellular_region_part | 19 | 0.21 | 0.91 | 0.55 | 0.68 | 1.00 | 297 |
| Cell_surface_receptor_linked_signal_transduction | 17 | 0.22 | 0.91 | 0.56 | 0.66 | 1.00 | 118 |
| Immune_response | 15 | 0.23 | 0.91 | 0.55 | 0.66 | 1.00 | 313 |
| Homeostatic_process | 16 | 0.21 | 0.88 | 0.61 | 0.70 | 1.00 | 294 |
| Anatomical_structure_morphogenesis | 22 | 0.19 | 0.86 | 0.61 | 0.71 | 1.00 | 248 |
| System_development | 42 | 0.15 | 0.84 | 0.67 | 0.73 | 1.00 | 248 |
| Regulation_of_cell_proliferation | 16 | 0.20 | 0.80 | 0.69 | 0.78 | 1.00 | 251 |
| Regulation_of_molecular_function | 19 | 0.18 | 0.79 | 0.72 | 0.79 | 1.00 | 147 |
| Anatomical_structure_development | 52 | 0.13 | 0.78 | 0.80 | 0.78 | 1.00 | 31 |
| Plasma_membrane | 62 | 0.11 | 0.68 | 0.89 | 0.90 | 1.00 | 205 |
| Nervous_system_development | 18 | 0.16 | 0.64 | 0.86 | 0.92 | 1.00 | 31 |
| Biopolymer_modification | 38 | 0.11 | 0.60 | 0.95 | 0.94 | 1.00 | 278 |
| Macromolecular_complex | 43 | -0.20 | -1.10 | 0.31 | 0.77 | 1.00 | 234 |
| Intrinsic_to_membrane | 52 | -0.19 | -1.10 | 0.33 | 0.82 | 1.00 | 247 |
| Protein_complex | 39 | -0.20 | -1.06 | 0.39 | 0.82 | 1.00 | 234 |
| Neurological_system_process | 16 | -0.28 | -1.11 | 0.32 | 0.84 | 1.00 | 86 |
| Organelle_organization_and_biogenesis | 20 | -0.22 | -0.96 | 0.54 | 0.85 | 1.00 | 119 |
| Intrinsic_to_plasma_membrane | 38 | -0.17 | -0.94 | 0.53 | 0.86 | 1.00 | 247 |
| Integral_to_plasma_membrane | 37 | -0.18 | -0.97 | 0.51 | 0.87 | 1.00 | 247 |
| Cellular_protein_metabolic_process | 57 | -0.15 | -0.92 | 0.57 | 0.87 | 1.00 | 241 |
| Plasma_membrane_part | 46 | -0.19 | -1.12 | 0.29 | 0.89 | 1.00 | 247 |
| Cellular_macromolecule_metabolic_process | 58 | -0.16 | -0.98 | 0.48 | 0.89 | 1.00 | 241 |
| Membrane | 78 | -0.13 | -0.89 | 0.64 | 0.89 | 1.00 | 234 |
| Establishment_of_localization | 44 | -0.18 | -0.98 | 0.49 | 0.93 | 1.00 | 234 |
| Transmembrane_transporter_activity | 25 | -0.24 | -1.12 | 0.32 | 0.95 | 1.00 | 301 |
| Macromolecule_biosynthetic_process | 16 | -0.15 | -0.57 | 0.95 | 0.95 | 1.00 | 225 |
| Transferase_activity_transferring_  phosphorus_containing_groups | 31 | -0.20 | -0.99 | 0.45 | 0.96 | 1.00 | 248 |
| Transmembrane_receptor_activity | 16 | -0.15 | -0.58 | 0.96 | 0.98 | 1.00 | 234 |
| Cell_cell_signaling | 21 | -0.15 | -0.67 | 0.88 | 0.99 | 1.00 | 711 |
| Phosphorylation | 26 | -0.15 | -0.72 | 0.84 | 0.99 | 1.00 | 210 |
| Protein_modification_process | 37 | -0.11 | -0.60 | 0.95 | 0.99 | 1.00 | 219 |
| Membrane_part | 61 | -0.22 | -1.37 | 0.09 | 1.00 | 1.00 | 234 |
| Cytoplasmic_part | 50 | -0.24 | -1.39 | 0.10 | 1.00 | 1.00 | 183 |
| Protein_serine_threonine_kinase_activity | 19 | -0.31 | -1.28 | 0.19 | 1.00 | 1.00 | 248 |
| Substrate_specific_transmembrane_  transporter_activity | 24 | -0.27 | -1.22 | 0.21 | 1.00 | 1.00 | 301 |
| Protein_kinase_activity | 22 | -0.28 | -1.24 | 0.21 | 1.00 | 1.00 | 248 |
| System_process | 25 | -0.26 | -1.20 | 0.22 | 1.00 | 1.00 | 301 |
| Ion_transmembrane_transporter_activity | 17 | -0.30 | -1.23 | 0.23 | 1.00 | 1.00 | 301 |
| Substrate_specific_transporter_activity | 25 | -0.25 | -1.18 | 0.26 | 1.00 | 1.00 | 301 |
| Kinase_activity | 27 | -0.24 | -1.15 | 0.28 | 1.00 | 1.00 | 248 |
| Phosphotransferase_activity_alcohol_  group_as_acceptor | 24 | -0.25 | -1.13 | 0.30 | 1.00 | 1.00 | 248 |
| Integral_to_membrane | 51 | -0.19 | -1.14 | 0.32 | 1.00 | 1.00 | 247 |
| Cell_fraction | 20 | -0.19 | -0.80 | 0.72 | 1.00 | 1.00 | 247 |
| Extracellular_region | 28 | -0.16 | -0.78 | 0.72 | 1.00 | 1.00 | 704 |
| Protein_amino_acid_phosphorylation | 24 | -0.17 | -0.75 | 0.79 | 1.00 | 1.00 | 241 |
| Protein_metabolic_process | 61 | -0.12 | -0.75 | 0.82 | 1.00 | 1.00 | 241 |
| Post_translational_protein_modification | 32 | -0.15 | -0.72 | 0.83 | 1.00 | 1.00 | 182 |
| Biosynthetic_process | 28 | -0.14 | -0.67 | 0.88 | 1.00 | 1.00 | 278 |
| Cellular_biosynthetic_process | 21 | -0.14 | -0.62 | 0.90 | 1.00 | 1.00 | 278 |
| **UPREGULATED in SUC/SIV** |  |  |  |  |  |  |  |

| Macromolecular_complex | 43 | -0.20 | -1.10 | 0.31 | 0.77 | 1 | 234 |
| --- | --- | --- | --- | --- | --- | --- | --- |
| Intrinsic_to_membrane | 52 | -0.19 | -1.10 | 0.33 | 0.82 | 1 | 247 |
| Protein_complex | 39 | -0.20 | -1.06 | 0.39 | 0.82 | 1 | 234 |
| Neurological_system_process | 16 | -0.28 | -1.11 | 0.32 | 0.84 | 1 | 86 |
| Organelle_organization_and_biogenesis | 20 | -0.22 | -0.96 | 0.54 | 0.85 | 1 | 119 |
| Intrinsic_to_plasma_membrane | 38 | -0.17 | -0.94 | 0.53 | 0.86 | 1 | 247 |
| Integral_to_plasma_membrane | 37 | -0.18 | -0.97 | 0.51 | 0.87 | 1 | 247 |
| Cellular_protein_metabolic_process | 57 | -0.15 | -0.92 | 0.57 | 0.87 | 1 | 241 |
| Plasma_membrane_part | 46 | -0.19 | -1.12 | 0.29 | 0.89 | 1 | 247 |
| Cellular_macromolecule_metabolic_process | 58 | -0.16 | -0.98 | 0.48 | 0.89 | 1 | 241 |
| Membrane | 78 | -0.13 | -0.89 | 0.64 | 0.89 | 1 | 234 |
| Establishment_of_localization | 44 | -0.18 | -0.98 | 0.49 | 0.93 | 1 | 234 |
| Transmembrane_transporter_activity | 25 | -0.24 | -1.12 | 0.32 | 0.95 | 1 | 301 |
| Macromolecule_biosynthetic_process | 16 | -0.15 | -0.57 | 0.95 | 0.95 | 1 | 225 |
| Transferase_activity_transferring_  Phosphorus_containing_groups | 31 | -0.20 | -0.99 | 0.45 | 0.96 | 1 | 248 |
| Transmembrane_receptor_activity | 16 | -0.15 | -0.58 | 0.96 | 0.98 | 1 | 234 |
| Cell_cell_signaling | 21 | -0.15 | -0.67 | 0.88 | 0.99 | 1 | 711 |
| Phosphorylation | 26 | -0.15 | -0.72 | 0.84 | 0.99 | 1 | 210 |
| Protein_modification_process | 37 | -0.11 | -0.60 | 0.95 | 0.99 | 1 | 219 |
| Membrane_part | 61 | -0.22 | -1.37 | 0.09 | 1.00 | 0.998 | 234 |
| Cytoplasmic_part | 50 | -0.24 | -1.39 | 0.10 | 1.00 | 0.998 | 183 |
| Protein_serine_threonine_kinase_activity | 19 | -0.31 | -1.28 | 0.19 | 1.00 | 1 | 248 |
| Substrate_specific_transmembrane_  Transporter_activity | 24 | -0.27 | -1.22 | 0.21 | 1.00 | 1 | 301 |
| Protein_kinase_activity | 22 | -0.28 | -1.24 | 0.21 | 1.00 | 1 | 248 |
| System_process | 25 | -0.26 | -1.20 | 0.22 | 1.00 | 1 | 301 |
| Ion_transmembrane_transporter_activity | 17 | -0.30 | -1.23 | 0.23 | 1.00 | 1 | 301 |
| Substrate_specific_transporter_activity | 25 | -0.25 | -1.18 | 0.26 | 1.00 | 1 | 301 |
| Kinase_activity | 27 | -0.24 | -1.15 | 0.28 | 1.00 | 1 | 248 |
| Phosphotransferase_activity_alcohol_as_acceptor | 24 | -0.25 | -1.13 | 0.30 | 1.00 | 1 | 248 |
| Integral_to_membrane | 51 | -0.19 | -1.14 | 0.32 | 1.00 | 1 | 247 |
| Cell_fraction | 20 | -0.19 | -0.80 | 0.72 | 1.00 | 1 | 247 |
| Extracellular_region | 28 | -0.16 | -0.78 | 0.72 | 1.00 | 1 | 704 |
| Protein_amino_acid_phosphorylation | 24 | -0.17 | -0.75 | 0.79 | 1.00 | 1 | 241 |
| Protein_metabolic_process | 61 | -0.12 | -0.75 | 0.82 | 1.00 | 1 | 241 |
| Post_translational_protein_modification | 32 | -0.15 | -0.72 | 0.83 | 1.00 | 1 | 182 |
| Biosynthetic_process | 28 | -0.14 | -0.67 | 0.88 | 1.00 | 1 | 278 |
| Cellular_biosynthetic_process | 21 | -0.14 | -0.62 | 0.90 | 1.00 | 1 | 278 |

ES - Enrichment score / NES - Normalized enrichment score / Nominal p-value / FDR - False discovery rate
