# Supplementary material for: Chronic binge alcohol administration dysregulates global regulatory gene networks associated with skeletal muscle wasting in simian immunodeficiency virus-infected macaques
Source: BMC Genomics. 2015 Dec 23;16:1097. doi: 10.1186/s12864-015-2329-z (PMC4690320; doi:10.1186/s12864-015-2329-z)
Supplement: Additional file 2: Table S2. — CBA-dependent alterations in mRNA expression at end-stage SIV infection. (DOCX 48 kb) [file 12864_2015_2329_MOESM2_ESM.docx]

**Additional file 2: Table S2: CBA-dependent alterations in mRNA expression at end-stage SIV infection**

| **Downregulated genes** | | | |
| --- | --- | --- | --- |
|  | | **Fold Change** | |
| *Enzymes/Enzymatic Activity* | |  | |
| Amino acid synthesis/degradation: | |  | |
| AASS | Promotes lysine degradation | -1.80 | |
| AMT | Catalyzes the degradation of glycine | -2.09 | |
| APIP | Functions in the methionine salvage pathway | -1.81 | |
| GLS | Involved in the catabolism of glutamine | -1.67 | |
| GNMT | Methylates glycine during the metabolism of Methionine | -1.66 | |
| GPT2 | Transamination to provide pyruvate and glutamate | -1.63 | |
| ILVBL | First step in branched chain amino acid synthesis | -1.62 | |
| Small molecule modifiers: | |  | |
| CA2 | Carbonic anhydrase - hydrates CO2 | -1.67 | |
| PDE4A | Cyclic nucleotide phosphodiesterase - hydrolyzes cAMP | -2.59 | |
| PLCD4 | Phospholipase - hydrolyzes PIP2 to DAG and IP3 | -2.43 | |
| PTGIS | Catalyzes the isomerization of prostaglandin | -2.14 | |
| SULT1A2 | Sulfate conjugation of hormones, neurotransmitters, and drugs | -1.67 | |
| TPMT | S-methylation of thiopurine drugs | -3.39 | |
| Lipid enzymology: | |  | |
| AGPAT5 | Involved in de novo phospholipid synthesis | -1.55 | |
| ST8SIA5 | Golgi protein - synthesis of gangliosides | -1.73 | |
| CHPT1 | Catalyzes phosphatidyl choline biosynthesis | -1.63 | |
| PIGZ | Mannosyltransferase – glycosylphosphatidylinositol-anchor biosynthesis | -1.60 | |
| THRSP | Regulates lipogenesis | -2.01 | |
| Protein modifications/proteases: | |  | |
| ADAMTS8 | A disintigrin and metalloproteinase | -2.38 | |
| B3GALTL | Glucosyltransferase enzyme | -2.06 | |
| ECE1 | Processing of endothelin precursors | -1.54 | |
| FICD | Adenylyltransferase - regulates proteins by transferring AMP | -1.59 | |
| PSMD10 | Chaperone for the assembly of the 26S proteasome | 1.57 | |
| SCPEP1 | Serine carboxypeptidase | 1.59 | |
| Ligand biosynthesis: | |  | |
| ALDH1A2 | Aldehyde dehydrogenase - synthesis of retinoic acid | -1.71 | |
| CYP26B1 | Cytochrome P450 family member - metabolism of retinoic acid | -1.58 | |
| HSD3B7 | Biosynthesis of hormonal steroids | -1.57 | |
| Miscellaneous: | |  | |
| ALAS2 | Mitochondrial - catalyzes first step in heme biosynthesis | -2.26 | |
| MSRB3 | Reduces methionine sulfoxide to methionine | -2.00 | |
| UROS | Porphyryn and heme biosynthesis | -1.62 | |
| Saccharide/sugar enzymology: | |  | |
| EXTL2 | Glycosyltransferase - biosynthesis of heparin sulfate | -2.06 | |
| HS6ST1 | Heparin sulfate biosynthetic enzyme - neuronal development | -1.57 | |
| XYLT2 | xylosyltransferase - first step in biosynthesis of glycosaminoglycans | -1.58 | |
| Fatty acid enzymology: | |  | |
| ACOT11 | Acyl-CoA thioesterase - converts fatty acids to non-fatty acid CoA form | -1.53 | |
|  | | | |
| *General Cellular Functioning* | | |  |
| Nucleotide biogenesis/regulation-related enzymes: | | |  |
| AARS2 | Alanyl-tRNA synthetase | | -1.91 |
| ADSSL1 | Regulates nucleotide levels (converts IMP to AMP) | | -1.51 |
| ALKBH8 | tRNA modification of Wobble position of anticodon loop | | -1.52 |
| APOBEC2 | C to U editing enzyme | | -1.66 |
| ENDOD1 | Possible DNase or RNase | | -1.58 |
| KRR1 | 40S ribosome biogenesis | | -1.55 |
| POLQ | Nicked double strand DNA polymerase - DNA repair | | -1.57 |
| Cellular organelles/cellular trafficking: | | |  |
| BICD1 | Involved in transport from Golgi to ER | | -1.64 |
| PLCL2 | May regulate Ins(1,4,5)P3 around endoplasmic reticulum | | -1.60 |
| REEP1 | ER network formation and remodeling | | -1.77 |
| SYPL2 | Communication between sarcoplasmic reticulum membranes | | -1.91 |
| TRAK1 | Involved in endosome to lysosome trafficking | | -1.61 |
| TTC8 | Coat complex to sort proteins to primary cilia | | -1.71 |
| Transcription/RNA splicing/translation: | | |  |
| CPEB3 | Poly-adenylation RNA binding protein | | -1.50 |
| EEF2K | Translational elongation factor | | -1.84 |
| SAMD4A | Acts as a translational repressor | | -1.50 |
| SETBP1 | Binds SET, which is involved in DNA replication | | -1.98 |
| SF3A1 | mRNA splicing factor | | -1.89 |
| YBX2 | Involved in regulating stability and translocation of mRNA | | -1.70 |
| Histone/histone function: | | |  |
| H2AFY2 | Histone H2A | | -1.86 |
| HIST1H4C | Histone cluster 1 H4c | | -1.61 |
| MLL5 | Histone methyltransferase - transcriptional activation | | -1.56 |
| SETD8 | Monomethylates histones and non-histone proteins | | -1.50 |
| Chromosome stability/function: | | |  |
| KLHDC3 | May be involved with meiotic recombination | | -1.51 |
| PTTG1 | Regulatory protein in chromosome stability | | -1.53 |
| Vesicle transport: | | |  |
| NAPA | Critical in docking and fusion or vesicles to target membranes | | -1.64 |
|  | | | |
| *Kinases and Phosphatases* | | |  |
| General kinases: | | |  |
| CSNK1G2 | Casein kinase 1 | | -1.88 |
| DCK | Phosphorylates deoxyribonucleic acids | | -1.51 |
| MAPK4 | Atypical MAP kinase - phosphorylates microtubule associate proteins | | -1.53 |
| MAST3 | Microtubule associated kinase | | -1.51 |
| PIK3C2B | Phosphoinositide 3-kinase family member | | -2.48 |
| PIK3CB | Catalytic subunit of PI3 kinase | | -1.79 |
| RPS6KB2 | Phosphorylates ribosomal protein S6 | | -1.51 |
| STK11 | Kinase involved in cell polarity | | -1.56 |
| WNK2 | Kinase involved with electrolyte homeostasis | | -1.63 |
| Kinases in signaling cascades: | | |  |
| LATS1 | Kinase in Hippo pathway - regulates proliferation, death and migration | | -1.51 |
| MAP3K10 | MAP Kinase kinase kinase - activate JNK pathway | | -1.90 |
| MAP3K12 | MAP kinase kinase kinase - JNK pathway | | -1.83 |
| TNIK | Kinase - essential activator of Wnt pathway | | -1.78 |
| Kinase/phosphatase regulators: | | |  |
| AKAP6 | Anchors PKA to endoplasmic reticulum membrane | | -1.55 |
| DUSP7 | Phosphatase - regulates MAP kinase | | -1.84 |
| PAQR3 | Regulates Raf1 kinase - sequesters it to Golgi | | -1.55 |
| PHLDA3 | Repressor of Akt function | | -1.63 |
| Phosphatases: | | |  |
| DUPD1 | Phosphatase and pro-isomerase | | -1.98 |
| PPP1R16A | Protein phosphatase 1 - regulatory subunit | | -1.95 |
| PPP2R3A | Protein phosphatase 2 - regulatory subunit | | -2.02 |
| PTPN3 | Protein tyrosine phosphatase | | -1.56 |
|  | | | |
| *Ion Channels* | | |  |
| Potassium (K^+^) channels: | | |  |
| ABCC9 | Subunit of ATP-sensitive K+ channel | | -1.91 |
| IRX5 | Negatively regulates K+ channel gene expression | | -1.99 |
| KCNA5 | K^+^ channel - may regulate the secretion of insulin | | -2.11 |
| KCNB1 | Voltage dependent K^+^ ion permeability of excited membranes | | -1.66 |
| KCNC4 | Voltage dependent K^+^ ion permeability of excited membranes | | -1.65 |
| KCNJ12 | Inwardly rectifying K^+^ channel - controls resting membrane potential | | -1.77 |
| KCNJ2 | Inward rectifying potassium channel | | -1.52 |
| KCNT1 | Na^+^ activated K^+^ channel | | -1.57 |
| LGI1 | Regulates voltage-gated potassium channels | | -3.78 |
| Sodium (Na^+^)/Chloride (Cl^-^) channels: | | |  |
| CLCNKA | Voltage gated chloride channel | | -1.52 |
| CLIC5 | Intracellular chloride channel | | -1.57 |
| SCN4B | Modulates sodium gating kinetics | | -3.16 |
| SLC12A2 | Na^+^ and Cl^-^ transport and readsorption | | -2.04 |
| SLC6A16 | Na^+^/Cl^-^ neurotransmitter transporter | | -1.81 |
| SLC26A10 | Chloride/bicarbonate exchanger | | -1.51 |
| SLC38A8 | Potential Na^+^ amino acid antiporter | | -2.06 |
| Miscellaneous channels/transporters: | | |  |
| CNGB1 | Cyclic nucleotide gated channel - nonselective cation channel | | -1.66 |
| PDZD2 | Bind cytoplasmic side of ion channels | | -1.77 |
| SLC36A2 | Proton-coupled amino acid transporter | | -1.81 |
| XK | Possible Na^+^-dependent transport of neutral amino acids | | -2.16 |
|  | | | |
| *Receptors* | | |  |
| Signal transduction receptors: | | |  |
| ESR1 | Estrogen receptor - nuclear hormone receptor | | -3.06 |
| FZD4 | Frizzled - receptor for Wnt signaling pathway | | -1.65 |
| GPR125 | G-protein coupled receptor | | -1.65 |
| LPHN2 | G-protein coupled receptor - cell adhesion | | -1.73 |
| RARB | Retinoic acid receptor - cell growth and differentiation | | -2.27 |
| Lipid receptors: | | |  |
| LRP12 | May be part of LDL receptor - internalization of lipophilic molecules | | -1.58 |
| OSBPL1A | Intracellular lipid receptor | | -1.52 |
| OSBPL6 | Oxysterol binding protein - intracellular lipid receptor | | -2.21 |
| Receptors for physiologic responses: | | |  |
| NPR3 | Natriuretic peptide receptor - regulate blood volume and pressure | | -3.42 |
| TMEM158 | Similar to receptor involved in neural survivals | | -1.73 |
| UNC5A | Receptor required for axon guidance | | -1.59 |
| Neuronal receptors: | | |  |
| DLG2 | Required for perception of chronic pain - interacts with NMDA receptor | | -1.83 |
| RIC3 | Promotes the expression of acetylcholine receptors | | -1.80 |
| Regulators of uptake/transport: | | |  |
| SHBG | Androgen transport protein - receptor mediated processes | | -2.36 |
| TFRC | Transferrin receptor - regulate uptake of iron | | -2.28 |
|  | | | |
| *Transcriptional regulation* | | |  |
| Transcription factors/activators: | | |  |
| ATP1B4 | Transcriptional regulator in muscle development - TGFbeta signaling | | -2.52 |
| CAMTA1 | Transcriptional activator - tumor suppressor | | -1.95 |
| IRX3 | Transcription factor - SHH-dependent neural patterning | | -2.29 |
| NFYB | Transcriptional activator of collagen and beta-actin (among others) | | -1.52 |
| PITX1 | Transcriptional regulator - hormone regulated activity of prolactin | | -1.59 |
| SOX18 | Transcription factor with unknown targets | | -1.56 |
| VGLL2 | May activate gene expression during muscle development | | -1.51 |
| ZNF3 | Zinc finger - involved in cell differentiation and/or cell proliferation | | -1.58 |
| ZNF165 | Possible transcriptional regulator | | -1.69 |
| ZNF784 | Zinc finger protein - possible transcriptional regulator | | -1.54 |
| Modified histones: | | |  |
| MTF2 | Binds trimethylated histone H3 - transcriptional regulator | | -1.58 |
| SMYD1 | Methylates histone H3 - transcriptional repressor | | -1.93 |
| Transcriptional cofactors/regulators: | | |  |
| LMCD1 | Transcriptional cofactor involved in cardiac hypertrophy | | -1.85 |
| MKL1 | Transcriptional coactivator - inhibits TNF induced cell death | | -1.58 |
| Transcriptional repressors: | | |  |
| HES5 | Transcriptional repressor involved in cell differentiation | | -1.86 |
|  | | | |
| *Ca^+2^-dependent functions* | | |  |
| Ca^+2^ channel/Ca^+2^ dependent channel: | | |  |
| ADRB2 | Beta adrenergic receptor - effector for Ca^+2^ channels | | -1.65 |
| ATP2B2 | Ca^+2^ export-dependent ATP hydrolase | | -5.10 |
| CACNG6 | Voltage dependent Ca^+2^ channel subunit | | -1.77 |
| KCNN3 | Intracellular Ca^+2^ activated potassium channel | | -1.68 |
| KCNIP3 | Ca^+2-^dependent regulator of K^+^ channels | | -1.50 |
| Ca^+2^ regulated enzymes/activities: | | |  |
| ADCY1 | Adenylate cyclase regulated by Ca^+2^ | | -1.54 |
| CADM1 | Ca^+2^-dependent cell adhesion molecule | | -2.17 |
| CALM2 | Ca^+2^-dependent regulator of kinases and phosphatases | | -1.57 |
| CIB2 | Calcium and integrin binding protein - Ca^+2^ binding regulatory protein | | -1.72 |
| TNNI1 | Confers Ca^+2^ sensitivity to muscle actomyosin | | -1.67 |
| Ca^+2^ mobilization: | | |  |
| CASQ1 | Muscle specific sarcoplasmic reticulum Ca^+2^ sensor | | -1.57 |
| JPH2 | Crosstalk between ECM and intracellular Ca^+2^ release | | -1.73 |
| Neuronal Ca^+2^ activities: | | |  |
| CAMK2A | Ca+2/Calmodulin dependent kinase - prominent in CNS | | -1.71 |
| KCNN2 | K^+^ channel activated by intracellular Ca^+2^ - regulates neuronal excitability | | -3.96 |
|  | | | |
| *Glycolysis/Energy metabolism* | | |  |
| Glycolysis/TCA cycle: | | |  |
| AACS | Acetoacetyl CoA synthetase - synthesizes Acetyl CoA | | -1.57 |
| PFKFB4 | Phosphofructo kinase – involved in glycolysis | | -1.72 |
| PGAM2 | Phosphoglycerate mutase – involved in the glycolytic pathway | | -1.85 |
| PPP1R3C | Regulatory subunit of phosphatase - limits glycogen breakdown | | -3.98 |
| PYGB | Mediates rate determining step in glycogen degradation | | -1.92 |
| SLC37A4 | Transports G-6-P from cytoplasm to lumen of the ER | | -2.17 |
| TPI1 | Triosephosphate isomerase – involved in glycolysis and gluconeogenesis | | -1.84 |
| Lipid/glycerol metabolism: | | |  |
| CROT | Fatty acid beta oxidation and lipid metabolism | | -2.06 |
| GK | Glycerol kinase - regulates glycerol uptake and metabolism | | -1.56 |
| GPD1 | Glycerol-3-phosphate dehydrogenase – carbohydrate/lipid metabolism | | -1.53 |
| Energy molecule biosynthesis: | | |  |
| COQ3 | Methylates coenzyme Q - CoQ biosynthesis | | -1.73 |
| GATM | Biosynthesis of guanidinoacetate – immediate precursor of creatine | | -5.06 |
| Insulin-related: | | |  |
| MAFB | Activates insulin and glucagon promoters | | -1.84 |
| SLC2A4 | Insulin regulated glucose transporter | | -1.90 |
| Miscellaneous: | | |  |
| COQ10A | Required for function of Coenzyme Q in respiratory pathway | | -1.81 |
|  | | | |
| *Ubiquitination/SUMOylation* | | |  |
| ASB10 | Substrate recognition for ECS E3 ubiquitin ligase | | -1.71 |
| ASB12 | Possible substrate recognition component of E3 ubiquitin ligase | | -1.58 |
| ASB15 | Possible substrate recognition component of E3 ubiquitin ligase | | -2.16 |
| CUL3 | Cullin - polyubiquitination and protein degradation | | -1.60 |
| FBXO3 | Substrate recognition for E3 ubiquitin ligase complex | | -1.74 |
| MAGEE1 | Enhance ubiquitin ligase activity | | -1.90 |
| MAGEF1 | May enhance ubiquitin ligase activity | | -2.00 |
| ZYG11B | Possible target recruitment of E3 ubiquitin ligase | | -2.37 |
| Ubiquitin enzymes: | | |  |
| HERC5 | Interferon-induced E3 ubiquitin ligase | | 1.53 |
| MARCH6 | E3 ubiquitin protein ligase | | -1.90 |
| RNF144B | E3 ubiquitin protein ligase | | -1.78 |
| USP15 | De-ubiquitinating enzyme | | -1.53 |
|  | | | |
| *Myogenic functions* | | |  |
| Muscle cell function: | | |  |
| ALKBH4 | Demethylates actin - regulates actin-myosin processes | | -1.51 |
| MYLK2 | Myosin light chain kinase - muscle contraction | | -2.09 |
| MYOZ3 | Helps tether calcineurin to sarcomeres in skeletal muscle | | -2.50 |
| PVALB | Parvalbumin - involved in relaxation after contraction | | -3.86 |
| TTN | Component in muscle assembly - balances forces between sarcomeres | | -1.51 |
| Muscle cell structure/integrity | | |  |
| CAV3 | Caveolin - involved in muscle membrane repair after mechanical stress | | -1.97 |
| CTF1 | Secreted cytokine that induces myocyte hypertrophy | | -1.53 |
| CTNNA3 | Involved in cell-cell adhesion in muscle cells | | -1.76 |
| SSPN | DGC network - link between cytoskeleton and ECM in muscle cells | | -1.77 |
| SYNC1 | Muscle intermediate filament protein - structural role in striated muscle | | -1.72 |
| Muscle cell biology: | | |  |
| MEF2C | Myogenic factor | | -1.59 |
| PITX2 | Controls cell proliferation and muscle expansion | | -1.61 |
| PTPLA | Muscle specific very long chain fatty acid synthesis | | -1.66 |
|  | | | |
| *Signaling Pathways* | | |  |
| Miscellaneous signaling: | | |  |
| IRS1 | Involved in insulin signaling | | -1.75 |
| PDE1A | Signal transduction - cyclic nucleotide phosphodiesterase | | -1.83 |
| Wnt signaling: | | |  |
| RSPO3 | Activator of canonical Wnt signaling | | -2.28 |
| WISP2 | Wnt signaling pathway - relevant for malignant transformation | | -1.58 |
| WNT4 | Ligand in the Wnt signaling pathway | | -1.58 |
| TGFbeta signaling: | | |  |
| SMAD6 | Negatively regulates TGF beta signaling | | -1.55 |
| TGFB2 | TGF beta - multiple effects | | -1.98 |
| TNF signaling: | | |  |
| IER3 | Protects cells from Fas or TNF induced apoptosis | | -1.72 |
| PEBP4 | Promotes cellular resistance to TNF-apoptosis | | -1.89 |
|  | | | |
| *Neuronal Function* | | |  |
| Neuronal survival/plasticity/regeneration: | | |  |
| ARMETL1 | Trophic factor for dopamine neurons | | -1.54 |
| MPDZ | Synaptic plasticity in excitatory synapses | | -1.86 |
| SEMA6C | Semaphorin possibly important for neural regeneration | | -1.67 |
| WDR62 | Neuronal proliferation and migration | | -1.81 |
| CNS development: | | |  |
| ARX | Transcription factor involved in CNS development | | -2.99 |
| CRIM1 | Interacts with growth factors during CNS development | | -1.74 |
| GPC4 | Cell surface heparin sulfate proteoglycan | | -2.05 |
| PHYHIPL | Possible role in development of CNS | | -2.36 |
| Neuronal orientation/polarization: | | |  |
| BTBD3 | Regulates dendritic field orientation | | -2.08 |
| EGFLAM | Necessary for proper bipolar dendritic tip apposition | | -1.69 |
| RILPL2 | Involved in cell shape and neuronal morphogenesis | | -1.52 |
| Neurotransmitters: | | |  |
| GLRB | Glycine receptor - neurotransmitter-gated ion channel | | -2.05 |
|  | | | |
| *Cell Growth/Cell Cycle Regulation* | | |  |
| Cell cycle regulators: | | |  |
| ARID4A | Binds to pRb to regulate cellular proliferation | | -2.00 |
| CABLES1 | Cell cycle dependent kinase binding protein | | -1.56 |
| CDKN3 | Cell cycle regulator - interacts with and dephosphorylates CDK2 | | -1.59 |
| NEDD1 | Required for mitosis progression | | -1.74 |
| SPIN2B | Regulates cell cycle progression | | -1.98 |
| Signal transduction cell growth regulation: | | |  |
| TOB1 | Anti-proliferative protein through ERBB signaling | | -2.31 |
| TRAF3IP3 | Mediates cell growth by modifying JNK pathway | | -1.66 |
|  | | | |
| *Transporters* | | |  |
| Cellular transporters: | | |  |
| NIPSNAP3B | Possible role in vesicular transport | | -3.06 |
| SLC29A1 | Mediates influx and efflux of nucleosides across membrane | | -1.69 |
| SLC35E4 | Putative transporter | | -2.41 |
| XPO4 | Regulates transport of proteins between cytoplasm and nucleus | | -1.60 |
| Small molecule/metal transport: | | |  |
| CNNM4 | Cycling M4 - possible metal transport | | -2.16 |
| HBA2 | Hemoglobin alpha - oxygen transport | | -2.21 |
| HBB | Hemoglobin beta chain | | -1.72 |
|  | | | |
| *Mitochondrial Function* | | |  |
| Mitochondrial maintenance: | | |  |
| KIF1B | Kinesin - anterograde transport of mitochondria | | -1.66 |
| OSGEPL1 | Involved in mitochondrial genome maintenance | | -1.98 |
| PINK1 | Mitochondrial kinase - protects against mitochondrial dysfunction | | -1.79 |
| Mitochondrial uptake/transport: | | |  |
| MRS2 | May mediate influx of Mg+2 into mitochondria | | -1.70 |
| SLC25A12 | Exchanges aspartate for glutamate across mitochondrial membrane | | -1.77 |
| SLC25A21 | Mitochondrial transporter of 2-oxoadipate - precursor of Acetyl CoA | | -1.66 |
|  | | | |
| *Miscellaneous* | | |  |
| CCDC28B | Modulates mTORC2 assembly and function | | -1.80 |
| FILIP1L | Regulator of antiangiogenic activity | | -2.19 |
| HIGD1B | Membrane associated - hypoxia inducible gene | | -1.76 |
| PER3 | Component of the circadian clock mechanism | | -1.60 |
| TMEM79 | Contributes to epidermal integrity | | -1.63 |
|  | | | |
| *Actin/Microtubule Proteins* | | |  |
| FSCN1 | Organizes actin filaments - migration and motility | | -1.72 |
| MAP7 | Microtubule associated protein - reorganization and polarization | | -3.00 |
| SGCB | Sarcoglycan - connects F-actin to ECM | | -2.35 |
| TMOD4 | Blocks elongation and depolymerization of actin ends | | -1.53 |
| XIRP2 | Protects actin filaments from depolymerization | | -1.61 |
|  | | | |
| *G-protein Coupled Activities* | | |  |
| ARHGAP18 | Rho GTPase activation protein | | -1.76 |
| ARHGEF10L | Guanine nucleotide exchange factor for Rho | | -1.51 |
| ARHGEF17 | Rho guanine nucleotide exchange factor | | -1.73 |
| RASGRP3 | Guanine nucleotide exchange factor for Ras and Raf | | -1.74 |
|  | | | |
| *Cellular Adhesion* | | |  |
| FRAS1 | ECM protein - involved in adhesion and organogenesis | | -1.72 |
| ITGA10 | Integrin alpha - receptor for collagen and important for adhesion | | -1.56 |
| LPP | Structural role at sites of adhesion, cell shape and motility | | -1.52 |
|  | | | |
| Differentiation and Development | | |  |
| SLFN5 | May be involved in hematopoietic cell differentiation | | -1.81 |
| TSPAN33 | Involved with erythropoiesis | | -1.83 |
| ZFPM2 | Involved in heart morphogenesis and coronary vessel development | | -2.01 |
|  | | | |
| *Extracellular Matrix* | | |  |
| ADAMTSL2 | Secreted glycoprotein - binds the cell surface and ECM | | -1.61 |
| CCDC88A | Enhances Akt signaling - cytoskeletal remodeling and cell migration | | -1.57 |
| IMPG2 | Proteoglycan - binds chondroitin sulfate and hyaluranon | | -1.72 |
|  | | | |
| *Cytoskeleton:* | | |  |
| PAK1 | Cell motility - link Rho GTPases to cytoskeletal reorganization | | -1.74 |
|  | | | |
| **Upregulated genes** | | | |
| *Enzymes/Enzymatic Activity* | | |  |
| Lipid enzymology: | | |  |
| DDHD1 | Phospholipase - hydrolyzes phosphatidic acid | | +2.21 |
| LIPG | Lipase - hydrolyzes HDL | | +2.71 |
| PLA1A | Hydrolysis of glycerophospholipids | | +1.53 |
| PLA2G2A | Regulates phospholipid metabolism | | +1.52 |
| PLCD3 | Phospholipase C - hydrolyzes PIP to diacylglycerol and inositol | | +1.86 |
| PON3 | Hydrolyzes lactones and inhibit oxidation of LDL | | +1.66 |
| Miscellaneous: | | |  |
| AKR1B10 | Aldo/keto reductase - reduce aliphatic and aromatic aldehydes | | +2.62 |
| LIPA | Lysosomal protein - catalyze hydrolysis of cholesterol and triglycerides | | +1.60 |
| METTL3 | Adenosine-methyltransferase - methylates internal A's on mRNA | | +1.73 |
| METTL6 | Probable methyltransferase | | +1.58 |
| SALL3 | Decreases DNMT-3 CpG methylation | | +2.05 |
| SARS | Serine tRNA aminoacyl transferase | | +1.58 |
| Saccharide/sugar enzymology: | | |  |
| B4GALT5 | Synthesis of complex n-linked oligosaccharides | | +2.23 |
| CHSY3 | Glycosyltransferase | | +1.58 |
| IDUA | Iduronidase alpha - hydrolyzes terminal alpha iduronic acid | | +2.06 |
| MAN1A2 | Mannosidase - maturation of Asn-linked oligosacharrides | | +1.64 |
| MPI | Enzyme involved in the synthesis of GDP-Mannose | | +1.84 |
| Acetyltranferases/deacetylaces: | | |  |
| CHD1 | Chromatin remodeler - member of histone acetyltransferase complex | | +1.66 |
| HDAC4 | Histone deacetylase 4 | | +2.36 |
| SIRT1 | Protein deacetylase - multiple functions | | +1.67 |
| YEATS4 | Member of histone acetyltransferase complex | | +1.67 |
| Fatty acid enzymology: | | |  |
| CPT1B | Long chain beta oxidation fatty acid pathway | | +1.66 |
| ELOVL5 | Synthesis of long chain fatty acids | | +2.11 |
| FADS1 | Promotes the desaturation of fatty acids | | +1.65 |
| ZDHHC7 | Broad specificity palmitoyltransferase | | +1.57 |
| Protein modification/proteases: | | |  |
| AEBP1 | Carboxypeptidase A family - reduce adipocyte differentiation | | +1.62 |
| CPA4 | Carboxypeptidase | | +3.13 |
| PAPPA | Metalloproteinase that cleave IGFBP 4 and 5 (increases free IGF) | | +3.13 |
| Amino acid synthesis/degradation: | | |  |
| BCAT1 | Catabolism of leucine, isoleucine, and valine | | +1.87 |
| PHGDH | Involved with L-serine synthesis | | +1.52 |
| PRPS2 | Involved with nucleotide synthesis | | +1.53 |
| Cell membrane protein anchoring: | | |  |
| HHATL | Negatively regulates palmitoylation of SHH | | +1.61 |
| PIGU | Part of GPI transamidase - attaches GP-anchors to proteins | | +2.08 |
| PIGW | Involved with the biosynthesis of the GPI anchor | | +2.00 |
| Protein folding/structure: | | |  |
| PPIL2 | Peptidylprolyl isomerase - facilitates folding of proteins | | +1.62 |
| SELM | Thiol-disulfide oxidoreductase - disulfide bond formation | | +1.56 |
|  | | | |
| *General Cellular Functioning* | | |  |
| Vesicle/organelle transport: | | |  |
| DYNC1I1 | Cytoplasmic dynein – motility of cytoplasmic vesicles and organelles | | +1.75 |
| EXOC5 | Assists docking of exocytic vesicles on plasma membrane | | +1.50 |
| EXPH5 | Possible involvement in vesicle transport | | +2.04 |
| GOLSYN | Part of kinesin motor adaptor complex - axonal transport | | +1.67 |
| GOPC | Intracellular protein trafficking and degradation | | +1.60 |
| SEC24D | Coat of ER-derived vesicles - transport from ER to Golgi | | +1.79 |
| VPS37B | Regulates vesicular trafficking | | +1.85 |
| Transcription/RNA splicing/translation: | | |  |
| CRKRS | CDK12 - regulates transcription elongation and mRNA splicing | | +1.53 |
| EAF1 | ELL transactivating factor - promote RNA Pol II transcription | | +2.09 |
| PRPF8 | Assists in the assembly of the snRNP complex | | +1.61 |
| RBM35B | Regulates mRNA splicing | | +1.51 |
| RPL29 | Ribosomal protein | | +1.53 |
| RPS21 | Ribosomal protein | | +1.78 |
| RNA processing: | | |  |
| EIF2C4 | Member of the RISC complex | | +2.01 |
| NOL11 | Nucleolar protein - rRNA processing | | +1.90 |
| RBM7 | Possible involvement in RNA processing | | +1.76 |
| TSEN2 | Involved in maturation of tRNA | | +1.52 |
| DNA repair: | | |  |
| MLH3 | DNA mismatch repair protein | | +1.53 |
| PMS1 | DNA mismatch repair protein | | +1.64 |
| REV3L | Involved in translesion DNA synthesis | | +1.58 |
| Histone/histone function: | | |  |
| BAZ1A | Part of a chromatin remodeling complex | | +1.52 |
| HIST1H1D | Histone H1 | | +1.52 |
| HIST1H2AC | Histone H1 | | +1.52 |
| Chromosome stability/function: | | |  |
| ESPL1 | Caspase-like protease - cleaves cohesin – chromosome segregation | | +1.72 |
| RCC2 | Regulator of chromosome condensation | | +1.51 |
| Nuclear pore/nuclear membrane integrity: | | |  |
| NUP188 | Nucleoporin - present in nuclear pore complex | | +1.82 |
| TMEM43 | Maintaining nuclear membrane structure | | +1.59 |
| Miscellaneous: | | |  |
| OFD1 | Centriole protein - involved in biogenesis of cilium | | +1.57 |
|  | | | |
| *Transcriptional Regulation* | | |  |
| Transcription factors – activators: | | |  |
| ARNTL | Transcriptional activator - circadian clock | | +1.62 |
| CCDC59 | Transcriptional activator | | +1.56 |
| ETS2 | Transcription factor - development/apoptosis - regulates telomeres | | +2.27 |
| FOS | Part of the FOS/Jun complex - transcriptional regulation | | +2.90 |
| JUNB | Part of the FOS/Jun complex - transcriptional regulation | | +2.61 |
| LITAF | LPS-induced - mediates TNF alpha expression | | +2.24 |
| PRRX2 | Role in fetal skin development | | +1.75 |
| Transcription co-factors/regulators: | | |  |
| ARID5B | Transcriptional coactivator involved in adipogenesis | | +1.83 |
| DDX21 | Helicase in Jun-activated transcription | | +1.60 |
| EGR1 | Transcriptional regulator of differentiation and mitogenesis | | +1.76 |
| NCOA7 | Enhances nuclear receptor transcriptional activity | | +1.99 |
| NFIL3 | Transcriptional regulator that binds to ATF sites | | +2.30 |
| Transcription inhibitors: | | |  |
| PHC1 | Polycomb group - maintain transcriptionally repressive state | | +1.91 |
| PTGES3 | Molecular chaperone - disrupts transcriptional activation complexes | | +1.57 |
|  | | | |
| *Cellular Adhesion* | | |  |
| Promotes cellular adhesion/cell-cell interactions: | | |  |
| CYR61 | Angiogenic inducer - promotes adhesion of endothelial cells | | +2.62 |
| EFNA1 | Ephrin ligand - promotes migration, repulsion and adhesion | | +1.60 |
| THBS1 | Mediates cell-cell and cell-matrix interactions | | +1.79 |
| THBS2 | Mediates cell-cell and cell-matrix interactions | | +1.87 |
| Ca^+2^-dependent adhesion molecules | | |  |
| CDH11 | Cadherin - Ca^+2^-dependent adhesion molecule | | +1.55 |
| CDH26 | Cadherin - Ca^+2^-dependent cell adhesion molecule | | +1.67 |
| PCDHA5 | Neuronal Ca^+2^-dependent cell adhesion protein | | +1.59 |
| Promotes loss of adhesion: | | |  |
| PNN | Reverses expression of E-cadherin expression | | +2.07 |
| PPFIA4 | Regulates the disassembly of focal adhesions | | +1.76 |
| RND3 | GTPase superfamily with no GTPase activity - promotes loss of adhesion | | +2.38 |
| Neuronal adhesion molecules: | | |  |
| NCAM1 | Neural cell adhesion molecule - cell-cell adhesion | | +1.66 |
| NINJ1 | Cell adhesion molecule that promotes axonal nerve growth | | +1.50 |
| Miscellaneous: | | |  |
| RHOB | Ras homolog - affects cell adhesion and growth factor signaling | | +2.38 |
| SEMA3F | Semaphorin - cell motility and adhesion | | +1.58 |
| Proteases/peptidases: | | |  |
| ADAM33 | Metallopeptidase involved in mediating cell-cell interactions | | +1.52 |
| RELN | ECM serine protease important for cell adhesion | | +1.78 |
|  | | | |
| *Extracellular Matrix* | | |  |
| Collagen and collagen deposition: | | |  |
| COL1A1 | Collagen type 1A | | +1.51 |
| COL1A2 | Collagen Type 1 | | +1.55 |
| CTHRC1 | Negative regulator of collagen matrix deposition | | +2.44 |
| LOX | Extracellular copper enzyme that initiates the crosslinking of collagens | | +2.37 |
| Proteases/peptidases: | | |  |
| HTRA3 | Serine protease - cleave ECM proteoglycans | | +2.17 |
| MMP11 | Matrixmetallopeptidase - ECM degradation | | +5.29 |
| PLAU | Serine protease involved in degradation of extracellular matrix | | +1.51 |
| Protease/peptidase inhibitors: | | |  |
| SERPINE1 | Ser proteinase inhibitor - inhibitor of fibrinolysis | | +2.48 |
| SERPINA1 | Inhibitor of serine proteases - elastin is primary target | | +1.52 |
| Cell surface proteoglycans: | | |  |
| SDC4 | Cell surface proteoglycan - binds heparin sulfate | | +1.54 |
| UGDH | Biosynthesis of glycosaminoglycans like chondroitin and heparin sulfate | | +2.04 |
| General ECM proteins | | |  |
| FREM2 | ECM protein - required for integrin of skin epithelium | | +1.65 |
| VCAN | Versican - member of the extracellular matrix | | +1.67 |
|  | | | |
| *Actin/Microtubule Proteins* | | |  |
| Actin polymerization/accumulation/reorganization: | | |  |
| ACTR3 | Regulator of actin polymerization | | +1.51 |
| SHRM | Controls cell shape through the accumulation of F-actin | | +1.50 |
| SPIRE1 | Actin nucleation factor - vesicular transport | | +1.64 |
| XIRP1 | Protect actin filaments from depolymerization | | +1.75 |
| Actin depolymerization/turnover: | | |  |
| MICAL1 | Promotes depolymerization of F-actin | | +2.47 |
| MICAL2 | Monooxygenase - depolymerization of F-actin | | +2.04 |
| RHPN2 | Binds GTP-Rho - increase turnover of F-actin structures | | +1.52 |
| Miscellaneous: | | |  |
| TUBA1B | Tubulin - major component of microtubules | | +1.53 |
| Structural: | | |  |
| NES | Member of the intermediate filament protein family | | +1.66 |
|  | | | |
| *Transporters* | | |  |
| Small molecule/metal transport: | | |  |
| SCARA5 | Mediates ferritin bound uptake of iron | | +1.73 |
| SLC2A3 | Glucose transporter | | +1.98 |
| SLC7A5 | Involved in cellular amino acid transport | | +1.77 |
| SLC16A6 | Solute carrier - rapid transport of monocarboxylates | | +4.06 |
| SLC16A10 | Mediates uptake of aromatic amino acids | | +1.72 |
| SLC39A6 | Zinc-influx transporter | | +1.73 |
| SLC39A14 | Zinc transporter | | +2.12 |
| SLC43A2 | Na^+^, Cl^-^, and pH-dependent transporter of large amino acids | | +1.58 |
| Molecular release: | | |  |
| GNRH1 | Stimulates release of gonadotropins | | +1.69 |
| SLCO2A1 | Mediate release of prostaglandin from cells | | +1.72 |
| Phagocytosis/endocytosis: | | |  |
| AAK1 | Regulates clathirin-mediated endocytosis | | +1.67 |
| SRPX | May be involved in phagocytosis | | +1.53 |
| Miscellaneous: | | |  |
| PLVAP | May function in microvascular permeability | | +2.55 |
|  | | | |
| *Cell Growth/Cell Cycle Regulation* | | |  |
| Growth inhibitory factors: | | |  |
| BTG1 | Anti-proliferative protein - high in G0 phase | | +1.63 |
| DDIT4L | Inhibits cell growth by regulating TOR signaling | | +1.73 |
| FBXO31 | Involved with G1 arrest following DNA damage | | +1.63 |
| LATS2 | Negative regulator - inhibits proliferation/promotes apoptosis | | +2.44 |
| Cell cycle regulators: | | |  |
| CCND2 | Cyclin D2 - G1 progression | | +1.60 |
| CDKN1B | Cell cycle regulator involved in G1 arrest | | +1.53 |
| CKS2 | Promotes catalytic activity of cyclin dependent kinases | | +2.17 |
| Growth promoting factors: | | |  |
| MYC | Multifunctional transcription factor - cell growth | | +2.02 |
| NEK6 | Kinase required for metaphase progression | | +1.53 |
| WDR82 | Chromatin modification - regulates transition from mitosis to interphase | | +1.53 |
| Miscellaneous: | | |  |
| ZFP36L1 | Probably transcription factor - response to growth factors | | +1.58 |
|  | | | |
| *Receptors* | | |  |
| Miscellaneous: | | |  |
| GPR179 | Orphan receptor involved in vision | | +1.49 |
| LTBR | TNF receptor super family member - promotes apoptosis | | +2.66 |
| NR2F2 | RAR activated nuclear receptor | | +1.60 |
| PROCR | Receptor for activated protein C - blood coagulation | | +2.15 |
| TNFRSF6B | Decoy receptor that protects against apoptosis | | +1.84 |
| Acetylcholine receptor – neurotransmitter: | | |  |
| CHRNA1 | Acetylcholine receptor - ion conduction | | +2.95 |
| CHRNB1 | Acetylcholine receptor - ion conduction | | +1.95 |
| CHRND | Acetylcholine receptor - ion conduction | | +8.02 |
| G-protein coupled receptors: | | |  |
| GDI2 | GDP dissociation factor - release of GDP from G binding proteins | | +1.62 |
| GPR64 | G-protein coupled receptor - possibly involvement in epididymal function | | +1.64 |
| OR4B1 | G-protein coupled receptor - olfactory receptor | | +1.64 |
|  | | | |
| *Ubiquitination/SUMOylation* | | |  |
| Ubiquitin enzymes: | | |  |
| FBXO10 | Member of SCF E3 ubiquitin ligase complex | | +1.62 |
| FBXO30 | Member of E3 ubiquitin ligase complex | | +1.61 |
| RNF167 | E3 ubiquitin protein ligase (ring finger protein) | | +1.49 |
| TRIM9 | Ubiquitin ligase - proteasomal degradation | | +1.75 |
| SUMOylation: | | |  |
| RANBP2 | Enhances SUMOylation | | +1.97 |
| SAE1 | E1 ligase for SUMO and SUMOylation | | +1.61 |
| Ubiquitin-dependent degradation: | | |  |
| SPSB1 | Mediator of ubiquitin-dependent degradation | | +2.23 |
| USP8 | Ubiquitin-specific peptidase | | +1.67 |
| Deubiquitinating: | | |  |
| VCPIP1 | Deubiquitinating enzyme post mitosis | | +1.62 |
|  | | | |
| *Myogenic Functions:* | | |  |
| Positive regulators: | | |  |
| CSRP3 | Positive regulator of myogenesis | | +2.68 |
| HES6 | Suppressor of HES1 (HES1 is a negative regulator of myogenesis) | | +1.90 |
| MYH3 | Myosin heavy chain 3 - muscle contraction | | +2.10 |
| MYH8 | Myosin heavy chain 8 - muscle contraction | | +3.27 |
| Muscle cell functions: | | |  |
| ABHD2 | Possible smooth muscle migration | | +1.68 |
| ANKRD1 | Transcription factor – myofibrillar stretch sensory | | +2.02 |
| GJA1 | Heart gap junction gene - important for synchronized contraction | | +3.44 |
| Negative regulator: | | |  |
| TMEM119 | May promote differentiation of myoblasts into osteoblasts | | +1.85 |
|  | | | |
| *Signaling Pathways* | | |  |
| Wnt/FGF signaling: | | |  |
| SFRP1 | Soluble frizzled like protein - modulates Wnt signaling | | +1.65 |
| SHISA2 | Attenuates FGF and Wnt signaling | | +2.42 |
| TLE3 | Transcriptional co-repressor in Wnt signaling | | +1.55 |
| Miscellaneous: | | |  |
| ABHD6 | May be a regulator of endocannabinoid signaling pathways | | +1.72 |
| HEY2 | Downstream Notch effector - cardiovascular development | | +1.53 |
|  | | | |
| *Kinases/Phosphatases* | | |  |
| General kinases: | | |  |
| PIM1 | Ser/Thr kinase - prevent apoptosis and promote survival | | +2.24 |
| PIM2 | Ser/Thr kinase - prevent apoptosis and promote survival | | +2.30 |
| PRKCI | Protein kinase C iota | | +1.56 |
| SNF1LK | Na^+^-inducible Ser/Thr kinase - multifunctional | | +1.71 |
| Miscellaneous: | | |  |
| CLP1 | Polynucleotide kinase - phosphorylate dsRNA | | +1.64 |
|  | | | |
| *Mitochondrial Function* | | |  |
| Miscellaneous: | | |  |
| MRPL38 | Mitochondrial ribosomal protein | | +1.76 |
| SOD2 | Mitochondrial superoxide dismutase | | +1.97 |
| Mitochondrial energy production: | | |  |
| CYP1B1 | Cytochrome P450 subunit B1 | | +2.09 |
| MTHFD1L | Tetrahydrofolate synthesis in the mitochondria | | +1.87 |
| Mitochondrial uptake/transport: | | |  |
| CCDC109B | Negatively modulates Ca^+2^ uptake into mitochondria | | +1.56 |
| DNAJA1 | Chaperon - transport of proteins into mitochondria | | +1.58 |
|  | | | |
| *Protein Folding/Turnover/Degradation* | | |  |
| Miscellaneous: | | |  |
| CTSL1 | Lysosomal cysteine proteinase - involved in protein turnover | | +1.51 |
| FKBP11 | Accelerate protein folding during protein synthesis | | +1.55 |
| PSMG3 | Chaperone promotes assembly of 20S proteasome | | +1.50 |
| UBLCP1 | Decreases proteolytic activity of 26S proteasome | | +1.97 |
| Receptor turnover/degradation: | | |  |
| CHMP1B | Involved in degradation of surface receptor proteins | | +1.52 |
| CHMP2B | Involved with the recycling or degradation of cell surface receptors | | +1.79 |
|  | | | |
| *Glycolysis/Energy Metabolism* | | |  |
| ADH1A | Alcohol dehydrogenase | | +1.51 |
| ADH1C | Alcohol dehydrogenase | | +1.52 |
| ENO1 | Enolase - glycolytic enzyme | | +1.89 |
| PDK4 | Ser/Thr kinase - glucose and fatty acid metabolism | | +2.26 |
| PFKFB3 | 6-phosphofructokinase – involved in glycolysis | | +1.60 |
| PFKP | Phosphofructokinase – involved in glycolysis | | +1.64 |
|  | | | |
| *Ca^+2^-Dependent Functions* | | |  |
| Ca^+2^ channel/Ca^+2^-dependent channel | | |  |
| BEST3 | Calcium sensitive chloride channels | | +1.78 |
| SRI | Ca^+2^ binding protein - modulates calcium channels | | +1.60 |
| STC1 | Stimulates phosphate and calcium reuptake | | +1.75 |
| Miscellaneous: | | |  |
| NCALD | Ca^+2^-dependent regulator of G-proteins (membrane associated) | | +1.67 |
| NUCB2 | Ca^+2^ binding - release of TNF from vascular endothelial cells | | +1.70 |
|  | | | |
| *Cytoskeleton* | | |  |
| LAMB1 | Laminin b - part of the basement membrane | | +1.86 |
| MARCKSL1 | Controls cell movement by regulating the actin cytoskeleton | | +1.80 |
| SEMA4D | Receptor for plexin - reorganization of actin cytoskeleton | | +2.01 |
| TCHH | Serves as scaffold protein to organize cell envelope | | +1.51 |
| VIL2 | Intermediate between plasma membrane and actin cytoskeleton | | +1.58 |
|  | | | |
| *Apoptotic* | | |  |
| ADAMTSL4 | Metalloproteinase - positive regulator of apoptosis | | +1.54 |
| GRAMD4 | Mediator of E2F1A apoptosis | | +1.51 |
| MEGF10 | Mediator of apoptotic cell phagocytosis | | +1.58 |
| TNFRSF10A | TNF family member - pro-apoptotic | | +1.71 |
|  | | | |
| *Neuronal Function* | | |  |
| CRYBB2 | Crystallin – possible neurite promoting factor | | +1.55 |
| DCLK1 | Involved with Ca^+2^ signaling - involved in neuronal migration | | +2.47 |
| FGF9 | Produced by neurons - growth stimulating effect on glial cells | | +1.91 |
| KIF5A | Kinesin protein - slow axonal transport of neurofilament proteins | | +1.54 |
|  | | | |
| *Differentiation and Development* | | |  |
| GDF15 | Bone morphogenic protein - member of TGFbeta family | | +1.52 |
| MGP | Associates with bone matrix - inhibits bone formation | | +1.80 |
| VEGFC | Promotes angiogenesis | | +1.54 |
|  | | | |
| *Metallothionein (Metal Binding/Anti-Oxidant)* | | |  |
| MT1A | Metallothionein protein | | +1.52 |
| MT1E | Metallothionein 1E - metal binding | | +1.91 |
| MT2A | Metallothionein protein | | +1.52 |
|  | | | |
| *Ion Transport* | | |  |
| ATP1A1 | ATP-dependent Na and K transport | | +1.73 |

*Gene name, function and fold-change vs. SUC/SIV macaques are shown for each category and sub-category according to their biological function.*
