# Supplementary material for: Chronic binge alcohol administration dysregulates global regulatory gene networks associated with skeletal muscle wasting in simian immunodeficiency virus-infected macaques
Source: BMC Genomics. 2015 Dec 23;16:1097. doi: 10.1186/s12864-015-2329-z (PMC4690320; doi:10.1186/s12864-015-2329-z)
Supplement: Additional file 3: Table S3. — Functional annotation summary and pathway summary of predicted target genes of CBA-altered microRNAs at end stage SIV infection. (DOCX 73 kb) [file 12864_2015_2329_MOESM3_ESM.docx]

**Additional file 3: Table S3: Functional annotation summary and pathway summary of predicted target genes of CBA-altered microRNAs at end stage SIV infection**

| **Category** | **Term** | **Total genes of term** | **Targets in term** | **Proportion of target in term** | **Proportion targets in targets** | **miRNAs in the term** | **Raw**  **p-value** | **Empirical p-value** |
| --- | --- | --- | --- | --- | --- | --- | --- | --- |
| Kegg | Pyrimidine_metabolism | 99 | 2 | 2.02 | 33.333 | 2 | 2.95E-04 | 1.80E-02 |
| Kegg | Purine_metabolism | 161 | 2 | 1.242 | 33.333 | 2 | 7.75E-04 | 2.47E-02 |
| Pid | E-cadherin_signaling_in_the_  nascent_adherens_junction | 38 | 1 | 2.632 | 16.667 | 2 | 1.03E-02 | 1.25E-01 |
| Reactome | Telomere_maintenance | 59 | 1 | 1.695 | 16.667 | 1 | 1.59E-02 | 1.45E-01 |
| Reactome | Metabolism_of_nucleotides | 70 | 1 | 1.429 | 16.667 | 2 | 1.88E-02 | 1.49E-01 |
| Reactome | Mitotic_m-m_g1_phases | 178 | 1 | 0.562 | 16.667 | 1 | 4.66E-02 | 1.50E-01 |
| Reactome | Dna_replication_pre-initiation | 82 | 1 | 1.22 | 16.667 | 1 | 2.19E-02 | 1.50E-01 |
| Reactome | M_g1_transition | 82 | 1 | 1.22 | 16.667 | 1 | 2.19E-02 | 1.50E-01 |
| Reactome | Dna_replication | 200 | 1 | 0.5 | 16.667 | 1 | 5.21E-02 | 1.64E-01 |
| Reactome | Synthesis_of_dna | 96 | 1 | 1.042 | 16.667 | 1 | 2.56E-02 | 1.64E-01 |
| Reactome | S_phase | 112 | 1 | 0.893 | 16.667 | 1 | 2.98E-02 | 1.64E-01 |
| Pid | Posttranslational_regulation_of_  adherens_junction_stability_and  _dissassembly | 48 | 1 | 2.083 | 16.667 | 2 | 1.29E-02 | 1.70E-01 |
| Reactome | Global_genomic_ner_(gg-ner) | 33 | 1 | 3.03 | 16.667 | 1 | 8.93E-03 | 1.73E-01 |
| Kegg | Base_excision_repair | 33 | 1 | 3.03 | 16.667 | 1 | 8.93E-03 | 1.73E-01 |
| Reactome | Dna_repair | 108 | 1 | 0.926 | 16.667 | 1 | 2.87E-02 | 1.76E-01 |
| Reactome | Cell_cycle_mitotic | 330 | 1 | 0.303 | 16.667 | 1 | 8.34E-02 | 1.88E-01 |
| Pid | Validated_targets_of_c-myc_  transcriptional_activation | 81 | 1 | 1.235 | 16.667 | 2 | 2.17E-02 | 1.91E-01 |
| Reactome | Chromosome_maintenance | 79 | 1 | 1.266 | 16.667 | 1 | 2.12E-02 | 1.93E-01 |
| Pid | Regulation_of_cdc42_activity | 30 | 1 | 3.333 | 16.667 | 2 | 8.13E-03 | 1.94E-01 |
| Reactome | Transcription-coupled_ner_(tc-ner) | 44 | 1 | 2.273 | 16.667 | 1 | 1.19E-02 | 2.12E-01 |
| Kegg | Nucleotide_excision_repair | 44 | 1 | 2.273 | 16.667 | 1 | 1.19E-02 | 2.12E-01 |
| Reactome | Nucleotide_excision_repair | 49 | 1 | 2.041 | 16.667 | 1 | 1.32E-02 | 2.12E-01 |
| Reactome | Extension_of_telomeres | 28 | 1 | 3.571 | 16.667 | 1 | 7.59E-03 | 2.13E-01 |
| Reactome | Activation_of_prereplicative_complex | 30 | 1 | 3.333 | 16.667 | 1 | 8.13E-03 | 2.16E-01 |
| Kegg | Dna_replication | 36 | 1 | 2.778 | 16.667 | 1 | 9.74E-03 | 2.20E-01 |
| Reactome | Mitotic_g1-g1_s_phases | 135 | 1 | 0.741 | 16.667 | 1 | 3.57E-02 | 2.36E-01 |
| Reactome | G1_s_transition | 109 | 1 | 0.917 | 16.667 | 1 | 2.90E-02 | 2.36E-01 |
| Pid | Arf6_trafficking_events | 49 | 1 | 2.041 | 16.667 | 2 | 1.32E-02 | 2.36E-01 |

| **Category** | **Term** | **Total genes in term** | **Union targets in term** | **miRNAs in the term** | **Score** |
| --- | --- | --- | --- | --- | --- |
| Reactome | Axon_guidance | 266 | 154 | 28 | 3.983 |
| Reactome | Developmental_biology | 494 | 229 | 28 | 3.716 |
| Kegg | Pathways_in_cancer | 325 | 173 | 28 | 2.951 |
| Reactome | L1cam_interactions | 94 | 61 | 26 | 2.77 |
| Kegg | Wnt_signaling_pathway | 150 | 91 | 28 | 2.466 |
| Kegg | Mapk_signaling_pathway | 272 | 135 | 29 | 2.466 |
| Kegg | Axon_guidance | 129 | 72 | 27 | 2.375 |
| GO MF | Protein_binding_transcription_factor_activity | 369 | 164 | 28 | 2.334 |
| Reactome | Signalling_by_ngf | 221 | 118 | 26 | 2.243 |
| Pid | Direct_p53_effectors | 137 | 65 | 27 | 2.062 |
| Kegg | Focal_adhesion | 199 | 99 | 27 | 1.992 |
| Reactome | Hemostasis | 467 | 191 | 29 | 1.989 |
| Kegg | Endocytosis | 201 | 102 | 27 | 1.944 |
| Kegg | Prostate_cancer | 89 | 49 | 26 | 1.941 |
| Kegg | Neurotrophin_signaling_pathway | 127 | 71 | 27 | 1.909 |
| Pid | Pdgfr-beta_signaling_pathway | 126 | 69 | 27 | 1.9 |
| Pid | Signaling_events_mediated_by_focal_adhesion_kinase | 58 | 43 | 25 | 1.875 |
| Pid | Regulation_of_nuclear_smad2_3_signaling | 82 | 52 | 27 | 1.847 |
| Kegg | Olfactory_transduction | 388 | 11 | 19 | 1.838 |
| Reactome | Neuronal_system | 289 | 120 | 28 | 1.837 |
| Kegg | Glioma | 65 | 35 | 27 | 1.836 |
| Reactome | Interaction_between_l1_and_ankyrins | 26 | 16 | 24 | 1.817 |
| Pid | Signaling_events_mediated_by_hepatocyte_  growth_factor_receptor_(c-met) | 77 | 50 | 26 | 1.74 |
| Kegg | Regulation_of_actin_cytoskeleton | 213 | 100 | 26 | 1.73 |
| Pid | E2f_transcription_factor_network | 73 | 44 | 25 | 1.717 |
| Reactome | Transmission_across_chemical_synapses | 190 | 85 | 28 | 1.701 |
| Pid | C-myb_transcription_factor_network | 81 | 46 | 24 | 1.683 |
| Reactome | Nuclear_receptor_transcription_pathway | 51 | 27 | 26 | 1.66 |
| Reactome | Ngf_signalling_via_trka_from_the_plasma_membrane | 136 | 78 | 26 | 1.637 |
| Pid | Ephb_forward_signaling | 36 | 27 | 22 | 1.619 |
| Kegg | Erbb_signaling_pathway | 87 | 48 | 27 | 1.614 |
| Kegg | Melanoma | 71 | 34 | 27 | 1.594 |
| Kegg | Melanogenesis | 101 | 55 | 28 | 1.582 |
| Pid | Erbb1_downstream_signaling | 106 | 62 | 23 | 1.58 |
| Kegg | Small_cell_lung_cancer | 84 | 43 | 26 | 1.563 |
| Pid | Notch_signaling_pathway | 59 | 37 | 25 | 1.55 |
| Kegg | Tgf-beta_signaling_pathway | 84 | 47 | 27 | 1.545 |
| Pid | Tcr_signaling_in_naive_cd4+_t_cells | 64 | 36 | 24 | 1.544 |
| Pid | Neurotrophic_factor-mediated_trk_receptor_signaling | 61 | 37 | 24 | 1.533 |
| Reactome | Signaling_by_egfr | 109 | 62 | 25 | 1.521 |
| Kegg | Pancreatic_cancer | 70 | 41 | 26 | 1.518 |
| Kegg | Fc_gamma_r-mediated_phagocytosis | 94 | 47 | 25 | 1.506 |
| Pid | Coregulation_of_androgen_receptor_activity | 57 | 35 | 26 | 1.5 |
| Kegg | Adherens_junction | 73 | 44 | 26 | 1.499 |
| Kegg | Tight_junction | 132 | 61 | 27 | 1.498 |
| Pid | Shp2_signaling | 54 | 34 | 25 | 1.485 |
| Pid | P73_transcription_factor_network | 73 | 34 | 25 | 1.462 |
| Reactome | Rho_gtpase_cycle | 124 | 58 | 25 | 1.455 |
| Reactome | Signaling_by_rho_gtpases | 124 | 58 | 25 | 1.455 |
| Reactome | Signaling_by_fgfr | 114 | 57 | 26 | 1.447 |
| Kegg | Chronic_myeloid_leukemia | 73 | 39 | 25 | 1.432 |
| Kegg | Renal_cell_carcinoma | 70 | 40 | 25 | 1.428 |
| Pid | Ap-1_transcription_factor_network | 69 | 41 | 25 | 1.41 |
| Reactome | G1_phase | 38 | 23 | 22 | 1.387 |
| Reactome | Cyclin_d_associated_events_in_g1 | 38 | 23 | 22 | 1.387 |
| Kegg | Dilated_cardiomyopathy | 90 | 42 | 24 | 1.384 |
| Reactome | Signaling_by_pdgf | 122 | 67 | 26 | 1.384 |
| Pid | Regulation_of_nuclear_beta_catenin_signaling_  and_target_gene_transcription | 79 | 44 | 24 | 1.376 |
| Reactome | Signaling_by_interleukins | 106 | 47 | 25 | 1.371 |
| Reactome | Downstream_signal_transduction | 93 | 53 | 25 | 1.354 |
| Kegg | Bacterial_invasion_of_epithelial_cells | 70 | 39 | 24 | 1.349 |
| Reactome | Adaptive_immune_system | 482 | 162 | 28 | 1.347 |
| Pid | Integrins_in_angiogenesis | 74 | 46 | 25 | 1.342 |
| Pid | Hif-1-alpha_transcription_factor_network | 65 | 35 | 26 | 1.337 |
| Kegg | Ubiquitin_mediated_proteolysis | 135 | 70 | 24 | 1.337 |
| Kegg | Oocyte_meiosis | 112 | 52 | 27 | 1.334 |
| Pid | Signaling_events_mediated_by_hdac_class_ii | 34 | 17 | 23 | 1.333 |
| Pid | Cxcr4-mediated_signaling_events | 102 | 54 | 25 | 1.323 |
| Reactome | Cd28_co-stimulation | 31 | 20 | 21 | 1.316 |
| Pid | Role_of_calcineurin-dependent_nfat_signaling_  in_lymphocytes | 55 | 35 | 23 | 1.31 |
| Reactome | Downstream_signaling_of_activated_fgfr | 100 | 50 | 25 | 1.309 |
| Pid | Cdc42_signaling_events | 70 | 40 | 25 | 1.29 |
| Kegg | Cell_cycle | 124 | 55 | 25 | 1.29 |
| Kegg | P53_signaling_pathway | 68 | 38 | 24 | 1.289 |
| Pid | Epha_forward_signaling | 34 | 18 | 20 | 1.282 |
| Pid | Posttranslational_regulation_of_adherens_junction_  stability_and_dissassembly | 48 | 29 | 24 | 1.28 |
| Reactome | Platelet_activation_signaling_and_aggregation | 205 | 85 | 27 | 1.278 |
| Pid | Regulation_of_retinoblastoma_protein | 64 | 35 | 27 | 1.275 |
| Pid | Hif-2-alpha_transcription_factor_network | 34 | 22 | 24 | 1.274 |
| Pid | Bcr_signaling_pathway | 68 | 36 | 25 | 1.274 |
| Pid | Signaling_events_regulated_by_ret_tyrosine_kinase | 38 | 22 | 22 | 1.266 |
| Reactome | Circadian_clock | 33 | 20 | 24 | 1.254 |
| Kegg | Long-term_potentiation | 70 | 40 | 25 | 1.252 |
| Pid | Tcr_signaling_in_naive_cd8+_t_cells | 51 | 29 | 20 | 1.241 |
| Reactome | Cytokine_signaling_in_immune_system | 220 | 77 | 27 | 1.236 |
| Kegg | Shigellosis | 61 | 36 | 23 | 1.233 |
| Kegg | Non-small_cell_lung_cancer | 54 | 25 | 23 | 1.217 |
| Kegg | Gnrh_signaling_pathway | 101 | 45 | 27 | 1.21 |
| Kegg | Vascular_smooth_muscle_contraction | 126 | 49 | 26 | 1.21 |
| Reactome | Mitotic_g1-g1_s_phases | 135 | 46 | 25 | 1.195 |
| Pid | Signaling_events_mediated_by_hdac_class_i | 67 | 31 | 22 | 1.183 |
| Pid | Foxo_family_signaling | 49 | 31 | 25 | 1.179 |
| Reactome | Netrin-1_signaling | 42 | 29 | 19 | 1.169 |
| Pid | Signaling_events_mediated_by_vegfr1_and_vegfr2 | 68 | 41 | 24 | 1.163 |
| Kegg | Insulin_signaling_pathway | 137 | 63 | 28 | 1.153 |
| Pid | Igf1_pathway | 29 | 21 | 23 | 1.147 |
| Reactome | Cell_cycle_mitotic | 330 | 95 | 27 | 1.146 |
| Pid | Vegfr1_specific_signals | 28 | 21 | 19 | 1.139 |
| Pid | Validated_targets_of_c-myc_transcriptional_repression | 63 | 31 | 25 | 1.137 |
| Reactome | P75_ntr_receptor-mediated_signalling | 86 | 40 | 23 | 1.135 |
| Kegg | Colorectal_cancer | 62 | 38 | 25 | 1.13 |
| Pid | P75(ntr)-mediated_signaling | 67 | 37 | 22 | 1.128 |
| Reactome | Cell_death_signalling_via_nrage_nrif_and_nade | 64 | 32 | 23 | 1.126 |
| Reactome | Transmembrane_transport_of_small_molecules | 427 | 145 | 28 | 1.125 |
| Pid | Atf-2_transcription_factor_network | 58 | 34 | 25 | 1.124 |
| Pid | Ifn-gamma_pathway | 42 | 30 | 24 | 1.12 |
| Reactome | Metabolism_of_lipids_and_lipoproteins | 292 | 89 | 27 | 1.119 |
| Pid | Internalization_of_erbb1 | 39 | 25 | 22 | 1.117 |
| Pid | Tgf-beta_receptor_signaling | 55 | 33 | 27 | 1.115 |
| Kegg | Calcium_signaling_pathway | 177 | 73 | 26 | 1.113 |
| Kegg | Hypertrophic_cardiomyopathy_(hcm) | 87 | 36 | 22 | 1.111 |
| Reactome | G_alpha_(12_13)_signalling_events | 77 | 36 | 22 | 1.111 |
| Pid | Syndecan-1-mediated_signaling_events | 46 | 27 | 22 | 1.11 |
| Pid | Lpa_receptor_mediated_events | 66 | 37 | 23 | 1.109 |
| Pid | Netrin-mediated_signaling_events | 30 | 23 | 19 | 1.098 |
| Reactome | Fatty_acid_triacylglycerol_and_ketone_body_metabolism | 112 | 40 | 21 | 1.096 |
| Pid | E-cadherin_signaling_in_the_nascent_adherens_junction | 38 | 27 | 22 | 1.094 |
| Pid | Bmp_receptor_signaling | 42 | 28 | 21 | 1.09 |
| Kegg | Chemokine_signaling_pathway | 189 | 77 | 28 | 1.088 |
| Reactome | Nrage_signals_death_through_jnk | 47 | 25 | 23 | 1.086 |
| Reactome | Cell-cell_communication | 129 | 60 | 25 | 1.083 |
| Kegg | T_cell_receptor_signaling_pathway | 108 | 50 | 25 | 1.083 |
| Reactome | Antigen_processing_ubiquitination_  proteasome_degradation | 213 | 86 | 26 | 1.078 |
| Pid | Fgf_signaling_pathway | 59 | 35 | 24 | 1.061 |
| Pid | Trk_receptor_signaling_mediated_by_pi3k_and_plc-gamma | 34 | 25 | 21 | 1.057 |
| Pid | Notch-mediated_hes_hey_network | 48 | 30 | 24 | 1.053 |
| Pid | Plasma_membrane_estrogen_receptor_signaling | 39 | 22 | 21 | 1.05 |
| Pid | Endothelins | 62 | 34 | 26 | 1.046 |
| Pid | Egf_receptor_(erbb1)_signaling_pathway | 32 | 19 | 20 | 1.046 |
| Kegg | Arrhythmogenic_right_ventricular_cardiomyopathy_(arvc) | 74 | 38 | 21 | 1.044 |
| Reactome | Class_i_mhc_mediated_antigen_processing_presentation | 251 | 95 | 26 | 1.044 |
| Pid | Noncanonical_wnt_signaling_pathway | 32 | 20 | 20 | 1.041 |
| Kegg | Gastric_acid_secretion | 74 | 39 | 27 | 1.038 |
| Reactome | Ncam_signaling_for_neurite_out-growth | 70 | 40 | 20 | 1.03 |
| Reactome | Neurotransmitter_receptor_binding_and_downstream_  transmission_in_the_postsynaptic_cell | 136 | 59 | 27 | 1.03 |
| Reactome | Regulation_of_lipid_metabolism_by_peroxisome_  proliferator-activated_receptor_alpha | 55 | 24 | 17 | 1.03 |
| Kegg | Gap_junction | 90 | 36 | 25 | 1.011 |
| Reactome | Signaling_by_scf-kit | 78 | 38 | 22 | 1.01 |
| Pid | Validated_nuclear_estrogen_receptor_alpha_network | 63 | 29 | 24 | 1.008 |
| Reactome | Signaling_by_robo_receptor | 32 | 18 | 19 | 1.007 |
| Kegg | Cell_adhesion_molecules_(cams) | 133 | 45 | 26 | 0.999 |
| Pid | Signaling_events_mediated_by_c-kit | 52 | 26 | 23 | 0.999 |
| Pid | Ephrin_b_reverse_signaling | 30 | 19 | 22 | 0.998 |
| Reactome | Factors_involved_in_megakaryocyte_development_  and_platelet_production | 125 | 54 | 26 | 0.996 |
| Pid | Validated_targets_of_c-myc_transcriptional_activation | 81 | 38 | 26 | 0.993 |
| Reactome | Insulin_receptor_signalling_cascade | 86 | 38 | 25 | 0.99 |
| Reactome | Transcriptional_regulation_of_  white_adipocyte_differentiation | 69 | 35 | 26 | 0.985 |
| Kegg | Notch_signaling_pathway | 47 | 25 | 23 | 0.983 |
| Pid | Tnf_receptor_signaling_pathway | 46 | 28 | 20 | 0.982 |
| GO MF | Structural_molecule_activity | 463 | 85 | 27 | 0.977 |
| Reactome | Clathrin_derived_vesicle_budding | 61 | 33 | 24 | 0.976 |
| Reactome | Trans-golgi_network_vesicle_budding | 61 | 33 | 24 | 0.976 |
| Kegg | Vegf_signaling_pathway | 76 | 31 | 24 | 0.974 |
| Reactome | G_alpha_(z)_signalling_events | 45 | 23 | 23 | 0.974 |
| Kegg | Chagas_disease | 104 | 47 | 26 | 0.973 |
| Reactome | Costimulation_by_the_cd28_family | 77 | 30 | 23 | 0.968 |
| Pid | Rhoa_signaling_pathway | 44 | 29 | 21 | 0.967 |
| Pid | Lkb1_signaling_events | 46 | 26 | 22 | 0.963 |
| Kegg | Bladder_cancer | 42 | 21 | 21 | 0.963 |
| Kegg | Progesterone-mediated_oocyte_maturation | 86 | 38 | 26 | 0.955 |
| Pid | N-cadherin_signaling_events | 36 | 24 | 20 | 0.954 |
| Reactome | Toll_like_receptor_9_(tlr9)_cascade | 85 | 42 | 23 | 0.951 |
| Reactome | Recycling_pathway_of_l1 | 28 | 20 | 17 | 0.951 |
| Pid | Il2-mediated_signaling_events | 54 | 31 | 25 | 0.949 |
| Pid | Syndecan-2-mediated_signaling_events | 33 | 21 | 18 | 0.947 |
| Pid | Presenilin_action_in_notch_and_wnt_signaling | 46 | 29 | 24 | 0.943 |
| Reactome | Toll_like_receptor_10_(tlr10)_cascade | 82 | 41 | 23 | 0.937 |
| Pid | Mtor_signaling_pathway | 68 | 40 | 24 | 0.937 |
| Reactome | Myd88_cascade_initiated_on_plasma_membrane | 82 | 41 | 23 | 0.937 |
| Reactome | Toll_like_receptor_5_(tlr5)_cascade | 82 | 41 | 23 | 0.937 |
| Pid | Class_i_pi3k_signaling_events | 47 | 22 | 23 | 0.937 |
| Kegg | Mtor_signaling_pathway | 52 | 26 | 22 | 0.932 |
| Reactome | Neurotransmitter_release_cycle | 36 | 18 | 18 | 0.928 |
| Kegg | Thyroid_cancer | 29 | 16 | 23 | 0.928 |
| Reactome | Interleukin-1_signaling | 40 | 21 | 19 | 0.928 |
| Reactome | Membrane_trafficking | 133 | 56 | 26 | 0.924 |
| Pid | Ceramide_signaling_pathway | 48 | 25 | 21 | 0.923 |
| Kegg | Toll-like_receptor_signaling_pathway | 102 | 40 | 18 | 0.923 |
| Reactome | Traf6_mediated_induction_of_nfkb_and_  map_kinases_upon_tlr7_8_or_9_activation | 80 | 40 | 23 | 0.922 |
| Reactome | Golgi_associated_vesicle_biogenesis | 54 | 29 | 24 | 0.916 |
| Reactome | Toll_like_receptor_7_8_(tlr7_8)_cascade | 81 | 40 | 23 | 0.913 |
| Pid | Regulation_of_androgen_receptor_activity | 52 | 25 | 24 | 0.913 |
| Reactome | Myd88_dependent_cascade_initiated_on_endosome | 81 | 40 | 23 | 0.913 |
| Reactome | Generic_transcription_pathway | 244 | 64 | 26 | 0.91 |
| Pid | Trk_receptor_signaling_mediated_by_the_mapk_pathway | 33 | 21 | 23 | 0.909 |
| Reactome | Map_kinase_activation_in_tlr_cascade | 49 | 30 | 22 | 0.907 |
| Reactome | Myd88_mal_cascade_initiated_on_plasma_membrane | 87 | 41 | 23 | 0.896 |
| Reactome | Toll_like_receptor_tlr6_tlr2_cascade | 87 | 41 | 23 | 0.896 |
| Reactome | Toll_like_receptor_tlr1_tlr2_cascade | 87 | 41 | 23 | 0.896 |
| Reactome | Toll_like_receptor_2_(tlr2)_cascade | 87 | 41 | 23 | 0.896 |
| Reactome | G0_and_early_g1 | 25 | 16 | 17 | 0.892 |
| Pid | Foxm1_transcription_factor_network | 41 | 20 | 18 | 0.892 |
| Pid | Il2_signaling_events_mediated_by_stat5 | 30 | 17 | 21 | 0.89 |
| Reactome | Signaling_by_insulin_receptor | 109 | 45 | 25 | 0.887 |
| Reactome | Regulation_of_insulin_secretion | 98 | 46 | 25 | 0.886 |
| Reactome | Pi-3k_cascade | 57 | 23 | 21 | 0.885 |
| Pid | Regulation_of_rhoa_activity | 46 | 25 | 19 | 0.877 |
| Kegg | B_cell_receptor_signaling_pathway | 75 | 30 | 24 | 0.876 |
| Reactome | Traf6_mediated_induction_of_proinflammatory_cytokines | 68 | 36 | 22 | 0.876 |
| Reactome | Nfkb_and_map_kinases_activation_mediated_by_tlr4_signaling_repertoire | 71 | 37 | 22 | 0.875 |
| Reactome | Activated_tlr4_signalling | 92 | 42 | 23 | 0.867 |
| Pid | Glucocorticoid_receptor_regulatory_network | 82 | 39 | 26 | 0.867 |
| Kegg | Endometrial_cancer | 52 | 25 | 24 | 0.866 |
| Reactome | Ion_channel_transport | 61 | 29 | 23 | 0.866 |
| Kegg | Salivary_secretion | 89 | 37 | 25 | 0.866 |
| Pid | Lissencephaly_gene_(lis1)_in_neuronal_migration_and_development | 31 | 16 | 20 | 0.865 |
| Pid | Il4-mediated_signaling_events | 64 | 31 | 26 | 0.864 |
| Pid | Regulation_of_p38-alpha_and_p38-beta | 31 | 21 | 22 | 0.863 |
| Pid | Regulation_of_cdc42_activity | 30 | 20 | 19 | 0.863 |
| Reactome | Semaphorin_interactions | 66 | 31 | 18 | 0.862 |
| Pid | Stabilization_and_expansion_of_the_e-cadherin_adherens_junction | 41 | 24 | 23 | 0.859 |
| Reactome | Integration_of_energy_metabolism | 125 | 56 | 25 | 0.855 |
| Pid | Validated_transcriptional_targets_of_tap63_isoforms | 51 | 25 | 23 | 0.854 |
| Pid | Reelin_signaling_pathway | 29 | 18 | 19 | 0.854 |
| Kegg | Phosphatidylinositol_signaling_system | 78 | 32 | 24 | 0.854 |
| Reactome | Diabetes_pathways | 229 | 56 | 27 | 0.852 |
| Reactome | Slc-mediated_transmembrane_transport | 250 | 79 | 25 | 0.852 |
| Reactome | Myd88-independent_cascade_initiated_on_plasma_membrane | 75 | 38 | 22 | 0.848 |
| Reactome | Integrin_cell_surface_interactions | 85 | 38 | 21 | 0.847 |
| Pid | Class_i_pi3k_signaling_events_mediated_by_akt | 34 | 23 | 18 | 0.847 |
| Kegg | Long-term_depression | 70 | 29 | 24 | 0.844 |
| Kegg | Ecm-receptor_interaction | 84 | 36 | 19 | 0.844 |
| Reactome | Apoptosis | 148 | 57 | 24 | 0.842 |
| Reactome | Irs-related_events | 81 | 35 | 25 | 0.842 |
| Reactome | Irs-mediated_signalling | 81 | 35 | 25 | 0.842 |
| Reactome | Plc-gamma1_signalling | 34 | 23 | 21 | 0.842 |
| Reactome | Toll_like_receptor_4_(tlr4)_cascade | 96 | 42 | 23 | 0.84 |
| Pid | Nectin_adhesion_pathway | 28 | 18 | 16 | 0.837 |
| Reactome | Toll_like_receptor_3_(tlr3)_cascade | 74 | 37 | 22 | 0.832 |
| Reactome | Trif_mediated_tlr3_signaling | 74 | 37 | 22 | 0.832 |
| Reactome | Toll_receptor_cascades | 108 | 44 | 23 | 0.827 |
| Reactome | Gpcr_ligand_binding | 410 | 92 | 28 | 0.825 |
| Pid | Signaling_events_mediated_by_tcptp | 41 | 22 | 20 | 0.824 |
| Pid | Signaling_mediated_by_p38-alpha_and_p38-beta | 38 | 23 | 20 | 0.823 |
| Reactome | Activation_of_nmda_receptor_upon_  glutamate_binding_and_postsynaptic_events | 37 | 25 | 22 | 0.823 |
| Pid | S1p3_pathway | 29 | 19 | 17 | 0.822 |
| Pid | P53_pathway | 58 | 26 | 20 | 0.821 |
| Kegg | Vasopressin-regulated_water_reabsorption | 44 | 22 | 20 | 0.818 |
| Reactome | Transport_of_inorganic_cations_anions_and_amino_acids_oligopeptides | 94 | 35 | 22 | 0.815 |
| Pid | Regulation_of_telomerase | 67 | 30 | 24 | 0.809 |
| Kegg | Pancreatic_secretion | 103 | 38 | 27 | 0.808 |
| Pid | Erbb2_erbb3_signaling_events | 42 | 25 | 21 | 0.807 |
| Reactome | Cell_junction_organization | 84 | 37 | 23 | 0.806 |
| Reactome | Dna_replication | 200 | 51 | 25 | 0.804 |
| Kegg | Adipocytokine_signaling_pathway | 68 | 31 | 21 | 0.803 |
| Kegg | Viral_myocarditis | 70 | 24 | 22 | 0.803 |
| Pid | Alpha4_beta1_integrin_signaling_events | 32 | 14 | 17 | 0.801 |
| Kegg | Toxoplasmosis | 132 | 47 | 22 | 0.801 |
| Pid | Angiopoietin_receptor_tie2-mediated_signaling | 49 | 22 | 23 | 0.799 |
| Reactome | Opioid_signalling | 80 | 40 | 23 | 0.797 |
| Pid | Osteopontin-mediated_events | 31 | 20 | 15 | 0.795 |
| Reactome | Mitotic_m-m_g1_phases | 178 | 45 | 25 | 0.795 |
| Kegg | Fc_epsilon_ri_signaling_pathway | 79 | 31 | 24 | 0.793 |
| Pid | Alk1_signaling_events | 25 | 14 | 18 | 0.793 |
| Reactome | Metabolism_of_mrna | 218 | 50 | 26 | 0.791 |
| Reactome | Gab1_signalosome | 39 | 19 | 20 | 0.789 |
| Pid | Il6-mediated_signaling_events | 45 | 26 | 20 | 0.788 |
| Pid | Nongenotropic_androgen_signaling | 30 | 19 | 21 | 0.787 |
| Pid | Insulin_pathway | 44 | 25 | 22 | 0.786 |
| Pid | Beta1_integrin_cell_surface_interactions | 65 | 34 | 20 | 0.784 |
| Reactome | Transport_to_the_golgi_and_subsequent_modification | 36 | 22 | 17 | 0.781 |
| Kegg | Acute_myeloid_leukemia | 57 | 28 | 21 | 0.779 |
| Reactome | Metabolism_of_rna | 264 | 68 | 26 | 0.777 |
| Reactome | Phospholipase_c-mediated_cascade | 54 | 27 | 23 | 0.776 |
| Pid | Calcineurin-regulated_nfat-dependent_transcription_  in_lymphocytes | 50 | 27 | 21 | 0.775 |
| Kegg | Cytokine-cytokine_receptor_interaction | 275 | 77 | 27 | 0.774 |
| Reactome | Innate_immune_system | 262 | 71 | 24 | 0.774 |
| Kegg | Jak-stat_signaling_pathway | 155 | 52 | 25 | 0.772 |
| Reactome | G_alpha_(s)_signalling_events | 125 | 43 | 23 | 0.771 |
| Reactome | Post_nmda_receptor_activation_events | 33 | 22 | 22 | 0.77 |
| Pid | Thromboxane_a2_receptor_signaling | 56 | 23 | 21 | 0.768 |
| Reactome | Dag_and_ip3_signaling | 31 | 21 | 19 | 0.766 |
| Reactome | Adherens_junctions_interactions | 29 | 17 | 22 | 0.763 |
| Pid | Rac1_signaling_pathway | 54 | 27 | 20 | 0.761 |
| Kegg | Protein_processing_in_endoplasmic_reticulum | 166 | 66 | 22 | 0.759 |
| Reactome | Tcr_signaling | 69 | 28 | 21 | 0.757 |
| Reactome | Signal_transduction_by_l1 | 35 | 22 | 16 | 0.756 |
| Reactome | Metabolism_of_proteins | 296 | 65 | 25 | 0.754 |
| Category | Term | Total genes in term | Union targets in term | miRNAs in the term | Score |
| Reactome | Effects_of_pip2_hydrolysis | 25 | 15 | 17 | 0.754 |
| Reactome | Interleukin-3_5_and_gm-csf_signaling | 45 | 16 | 22 | 0.752 |
| Reactome | Ncam1_interactions | 44 | 25 | 16 | 0.745 |
| Reactome | Pi3k_cascade | 70 | 29 | 25 | 0.744 |
| Kegg | Aldosterone-regulated_sodium_reabsorption | 42 | 22 | 21 | 0.744 |
| Pid | P38_mapk_signaling_pathway | 29 | 19 | 20 | 0.743 |
| Reactome | Signalling_to_erks | 35 | 19 | 20 | 0.742 |
| Kegg | Apoptosis | 88 | 38 | 21 | 0.74 |
| Kegg | Hepatitis_c | 134 | 47 | 20 | 0.74 |
| Reactome | Class_a_1_(rhodopsin-like_receptors) | 305 | 57 | 23 | 0.74 |
| Reactome | Signaling_by_notch | 25 | 14 | 16 | 0.739 |
| Reactome | Egfr_interacts_with_phospholipase_c-gamma | 33 | 21 | 19 | 0.739 |
| Pid | Signaling_events_mediated_by_ptp1b | 52 | 25 | 19 | 0.739 |
| Pid | Erbb4_signaling_events | 35 | 15 | 16 | 0.738 |
| Reactome | Cell-cell_junction_organization | 59 | 27 | 22 | 0.731 |
| Kegg | Hedgehog_signaling_pathway | 56 | 24 | 23 | 0.73 |
| Reactome | Trafficking_of_ampa_receptors | 30 | 16 | 19 | 0.73 |
| Reactome | Glutamate_binding_activation_of_ampa_  receptors_and_synaptic_plasticity | 30 | 16 | 19 | 0.73 |
| Kegg | Vibrio_cholerae_infection | 54 | 24 | 22 | 0.729 |
| Pid | Cxcr3-mediated_signaling_events | 42 | 22 | 23 | 0.729 |
| Reactome | Intrinsic_pathway_for_apoptosis | 30 | 22 | 16 | 0.729 |
| Pid | Regulation_of_rac1_activity | 38 | 20 | 20 | 0.729 |
| Reactome | Mitotic_prometaphase | 92 | 28 | 25 | 0.728 |
| Kegg | Leukocyte_transendothelial_migration | 116 | 45 | 24 | 0.727 |
| Reactome | Ion_transport_by_p-type_atpases | 36 | 20 | 21 | 0.725 |
| Kegg | Pathogenic_escherichia_coli_infection | 56 | 22 | 20 | 0.724 |
| Reactome | Cell_surface_interactions_at_the_vascular_wall | 94 | 36 | 22 | 0.723 |
| Pid | Beta3_integrin_cell_surface_interactions | 43 | 23 | 18 | 0.721 |
| Pid | Foxa1_transcription_factor_network | 44 | 19 | 24 | 0.719 |
| Reactome | M_phase | 96 | 29 | 25 | 0.719 |
| Pid | Gmcsf-mediated_signaling_events | 36 | 19 | 18 | 0.718 |
| Reactome | Response_to_elevated_platelet_cytosolic_ca2+ | 83 | 28 | 20 | 0.717 |
| Pid | Caspase_cascade_in_apoptosis | 56 | 28 | 20 | 0.714 |
| Pid | Regulation_of_ras_family_activation | 33 | 16 | 21 | 0.709 |
| Kegg | Snare_interactions_in_vesicular_transport | 36 | 16 | 18 | 0.708 |
| Reactome | Post-translational_protein_modification | 123 | 41 | 23 | 0.702 |
| Kegg | Basal_cell_carcinoma | 55 | 29 | 23 | 0.702 |
| Reactome | Potassium_channels | 99 | 34 | 24 | 0.701 |
| Pid | Hedgehog_signaling_events_mediated_by_gli_proteins | 48 | 27 | 21 | 0.701 |
| Reactome | Deadenylation-dependent_mrna_decay | 46 | 18 | 19 | 0.695 |
| Kegg | Amoebiasis | 105 | 41 | 22 | 0.688 |
| Kegg | Inositol_phosphate_metabolism | 57 | 21 | 18 | 0.687 |
| Pid | Il1-mediated_signaling_events | 35 | 16 | 14 | 0.686 |
| Reactome | Platelet_homeostasis | 81 | 33 | 26 | 0.685 |
| Kegg | Neuroactive_ligand-receptor_interaction | 318 | 76 | 25 | 0.683 |
| Reactome | G_alpha_(q)_signalling_events | 186 | 46 | 26 | 0.681 |
| Kegg | Rna_transport | 144 | 45 | 26 | 0.681 |
| Pid | Fc-epsilon_receptor_i_signaling_in_mast_cells | 61 | 27 | 18 | 0.671 |
| Reactome | Class_b_2_(secretin_family_receptors) | 90 | 27 | 24 | 0.671 |
| Kegg | Amyotrophic_lateral_sclerosis_(als) | 54 | 27 | 24 | 0.668 |
| Kegg | Lysosome | 121 | 42 | 21 | 0.667 |
| Reactome | Hiv_infection | 200 | 54 | 26 | 0.666 |
| Kegg | Epithelial_cell_signaling_in_helicobacter_pylori_infection | 68 | 30 | 21 | 0.663 |
| Pid | Par1-mediated_thrombin_signaling_events | 42 | 22 | 18 | 0.663 |
| Pid | Nephrin_neph1_signaling_in_the_kidney_podocyte | 31 | 19 | 18 | 0.663 |
| Reactome | Pi3k_akt_activation | 37 | 19 | 18 | 0.663 |
| Pid | Aurora_a_signaling | 31 | 17 | 17 | 0.659 |
| Pid | Glypican_1_network | 27 | 10 | 19 | 0.659 |
| Kegg | Huntington's_disease | 183 | 45 | 26 | 0.658 |
| Reactome | Cyclin_a_cdk2-associated_events_at_s_phase_entry | 66 | 22 | 17 | 0.658 |
| Reactome | Cyclin_e_associated_events_during_g1_s_transition | 65 | 21 | 17 | 0.657 |
| Reactome | Plc_beta_mediated_events | 43 | 26 | 20 | 0.655 |
| Reactome | Asparagine_n-linked_glycosylation | 85 | 31 | 19 | 0.655 |
| Reactome | Creb_phosphorylation_through_the_activation_of_ras | 27 | 17 | 17 | 0.653 |
| Reactome | Formation_and_maturation_of_mrna_transcript | 185 | 55 | 23 | 0.652 |
| Reactome | Mapk_targets_nuclear_events_mediated_by_map_kinases | 30 | 20 | 17 | 0.65 |
| Reactome | G-protein_mediated_events | 44 | 27 | 20 | 0.649 |
| Pid | Insulin-mediated_glucose_transport | 29 | 18 | 17 | 0.649 |
| Reactome | Sema4d_in_semaphorin_signaling | 29 | 13 | 14 | 0.644 |
| Reactome | Mrna_processing | 157 | 48 | 24 | 0.644 |
| Reactome | Platelet_degranulation | 78 | 26 | 20 | 0.643 |
| Reactome | Transcription | 177 | 40 | 26 | 0.641 |
| Reactome | Gaba_receptor_activation | 53 | 23 | 19 | 0.638 |
| Pid | Arf6_trafficking_events | 49 | 25 | 17 | 0.638 |
| Reactome | G1_s_transition | 109 | 31 | 18 | 0.636 |
| Reactome | Rna_polymerase_iii_abortive_and_retractive_initiation | 34 | 10 | 22 | 0.632 |
| Reactome | Rna_polymerase_iii_transcription | 34 | 10 | 22 | 0.632 |
| Reactome | Regulation_of_mrna_stability_by_proteins_that_bind_au-rich_elements | 86 | 27 | 22 | 0.632 |
| Kegg | Type_ii_diabetes_mellitus | 47 | 22 | 23 | 0.63 |
| Kegg | Phagosome | 154 | 39 | 21 | 0.628 |
| Reactome | Nod1_2_signaling_pathway | 31 | 15 | 18 | 0.628 |
| Pid | A6b1_and_a6b4_integrin_signaling | 44 | 19 | 20 | 0.628 |
| Pid | Il12_signaling_mediated_by_stat4 | 33 | 15 | 12 | 0.627 |
| Reactome | Metabolism_of_amino_acids_and_derivatives | 174 | 34 | 23 | 0.626 |
| Reactome | Lipid_digestion_mobilization_and_transport | 48 | 13 | 17 | 0.623 |
| Pid | Il2_signaling_events_mediated_by_pi3k | 37 | 23 | 17 | 0.618 |
| Pid | Syndecan-4-mediated_signaling_events | 31 | 18 | 16 | 0.615 |
| Reactome | Nucleotide-binding_domain_leucine_rich_repeat_  containing_receptor_(nlr)_signaling_pathways | 51 | 19 | 20 | 0.613 |
| Kegg | Nod-like_receptor_signaling_pathway | 62 | 25 | 18 | 0.61 |
| Pid | Validated_transcriptional_targets_of_ap1_family_members_fra1_and_fra2 | 35 | 18 | 21 | 0.609 |
| Reactome | Interferon_signaling | 110 | 28 | 22 | 0.608 |
| Reactome | Mitotic_g2-g2_m_phases | 87 | 24 | 22 | 0.608 |
| Kegg | Purine_metabolism | 161 | 41 | 21 | 0.605 |
| Reactome | Rig-i_mda5_mediated_induction_of_ifn-alpha_beta_pathways | 76 | 28 | 18 | 0.605 |
| Reactome | The_role_of_nef_in_hiv-1_replication_and_disease_pathogenesis | 29 | 13 | 17 | 0.605 |
| Reactome | Cdo_in_myogenesis | 29 | 16 | 20 | 0.603 |
| Reactome | Myogenesis | 29 | 16 | 20 | 0.603 |
| Reactome | S_phase | 112 | 29 | 18 | 0.6 |
| Pid | Downstream_signaling_in_naive_cd8+_t_cells | 68 | 24 | 21 | 0.596 |
| Reactome | Host_interactions_of_hiv_factors | 135 | 38 | 24 | 0.585 |
| Reactome | Rna_polymerase_i_rna_polymerase_iii_and_mitochondrial_transcription | 91 | 16 | 23 | 0.583 |
| Kegg | Natural_killer_cell_mediated_cytotoxicity | 140 | 35 | 23 | 0.58 |
| Pid | Il8-_and_cxcr1-mediated_signaling_events | 27 | 12 | 19 | 0.58 |
| Reactome | Degradation_of_beta-catenin_by_the_destruction_complex | 67 | 22 | 15 | 0.579 |
| Reactome | Signaling_by_wnt | 67 | 22 | 15 | 0.579 |
| Pid | Arf6_signaling_events | 35 | 17 | 18 | 0.577 |
| Reactome | Processing_of_capped_intron-containing_pre-mrna | 138 | 41 | 23 | 0.575 |
| Kegg | Protein_digestion_and_absorption | 80 | 31 | 22 | 0.572 |
| Reactome | Frs2-mediated_cascade | 38 | 13 | 19 | 0.565 |
| Reactome | Activation_of_chaperones_by_ire1alpha | 48 | 18 | 18 | 0.562 |
| Pid | Fas_(cd95)_signaling_pathway | 38 | 20 | 17 | 0.559 |
| Reactome | Signalling_to_ras | 27 | 15 | 17 | 0.558 |
| Reactome | Regulation_of_dna_replication | 75 | 19 | 19 | 0.555 |
| Pid | Wnt_signaling_network | 28 | 14 | 18 | 0.554 |
| Reactome | G_alpha_(i)_signalling_events | 200 | 43 | 20 | 0.549 |
| Kegg | Lysine_degradation | 44 | 17 | 20 | 0.548 |
| Pid | Aurora_b_signaling | 41 | 14 | 20 | 0.548 |
| Reactome | Voltage_gated_potassium_channels | 43 | 16 | 20 | 0.548 |
| Reactome | Downstream_tcr_signaling | 52 | 16 | 18 | 0.544 |
| Reactome | Influenza_infection | 149 | 20 | 17 | 0.541 |
| Pid | Urokinase-type_plasminogen_activator mediated_signaling | 42 | 13 | 14 | 0.54 |
| Reactome | Gpvi-mediated_activation_cascade | 33 | 17 | 14 | 0.54 |
| Reactome | Negative_regulation_of_fgfr_signaling | 40 | 13 | 17 | 0.539 |
| Reactome | Metabolism_of_carbohydrates | 126 | 34 | 20 | 0.538 |
| Reactome | Pip3_activates_akt_signaling | 28 | 13 | 16 | 0.537 |
| Pid | Integrin-linked_kinase_signaling | 45 | 18 | 19 | 0.537 |
| Reactome | Unfolded_protein_response | 66 | 25 | 19 | 0.537 |
| Kegg | Rna_degradation | 57 | 16 | 19 | 0.536 |
| GO MF | Channel_regulator_activity | 55 | 21 | 19 | 0.532 |
| Reactome | Apoptotic_execution_phase | 52 | 21 | 19 | 0.531 |
| Pid | Il3-mediated_signaling_events | 27 | 12 | 18 | 0.531 |
| Pid | Retinoic_acid_receptors-mediated_signaling | 27 | 14 | 20 | 0.53 |
| Kegg | Prion_diseases | 36 | 14 | 16 | 0.529 |
| Kegg | Glycosaminoglycan_biosynthesis_heparan_sulfate | 26 | 10 | 11 | 0.528 |
| Pid | Epo_signaling_pathway | 34 | 14 | 22 | 0.524 |
| Reactome | Triglyceride_biosynthesis | 34 | 12 | 15 | 0.523 |
| Reactome | Apoptotic_cleavage_of_cellular_proteins | 38 | 18 | 18 | 0.522 |
| Reactome | Influenza_life_cycle | 144 | 18 | 16 | 0.521 |
| Reactome | Sphingolipid_metabolism | 32 | 13 | 13 | 0.521 |
| Pid | Integrin_family_cell_surface_interactions | 26 | 11 | 14 | 0.519 |
| Pid | Validated_transcriptional_targets_of_deltanp63_isoforms | 45 | 20 | 18 | 0.516 |
| Reactome | Interferon_gamma_signaling | 73 | 21 | 20 | 0.513 |
| Reactome | Glucose_metabolism | 62 | 19 | 16 | 0.507 |
| Reactome | Integrin_alphaiib_beta3_signaling | 27 | 14 | 16 | 0.506 |
| Reactome | Regulation_of_water_balance_by_renal_aquaporins | 40 | 15 | 17 | 0.504 |
| Reactome | Mrna_splicing_major_pathway | 107 | 28 | 22 | 0.504 |
| Reactome | Mrna_splicing | 107 | 28 | 22 | 0.504 |
| Kegg | Intestinal_immune_network_for_iga_production | 48 | 14 | 11 | 0.502 |
| Kegg | Alzheimer's_disease | 168 | 42 | 20 | 0.498 |
| Kegg | Spliceosome | 127 | 33 | 21 | 0.497 |
| Reactome | M_g1_transition | 82 | 16 | 19 | 0.497 |
| Reactome | Interleukin-2_signaling | 42 | 16 | 18 | 0.497 |
| Reactome | Dna_replication_pre-initiation | 82 | 16 | 19 | 0.497 |
| Pid | Trail_signaling_pathway | 28 | 12 | 14 | 0.496 |
| Pid | Cd40_cd40l_signaling | 30 | 15 | 17 | 0.495 |
| Reactome | Egfr_downregulation | 27 | 13 | 18 | 0.494 |
| Reactome | Peptide_ligand-binding_receptors | 186 | 35 | 18 | 0.493 |
| Pid | Signaling_events_mediated_by_hdac_class_iii | 39 | 14 | 18 | 0.487 |
| Reactome | Cell_cycle_checkpoints | 117 | 28 | 18 | 0.485 |
| Reactome | Aquaporin-mediated_transport | 47 | 17 | 19 | 0.481 |
| Kegg | Rig-i-like_receptor_signaling_pathway | 71 | 27 | 19 | 0.48 |
| Reactome | Loss_of_nlp_from_mitotic_centrosomes | 62 | 14 | 19 | 0.478 |
| Reactome | Loss_of_proteins_required_for_interphase_microtubule_  Organization From_the_centrosome | 62 | 14 | 19 | 0.478 |
| Kegg | Glycerophospholipid_metabolism | 79 | 19 | 19 | 0.476 |
| Reactome | Regulation_of_mitotic_cell_cycle | 82 | 20 | 19 | 0.475 |
| Reactome | G1_s_dna_damage_checkpoints | 60 | 17 | 15 | 0.475 |
| Reactome | Apc_c-mediated_degradation_of_cell_cycle_proteins | 82 | 20 | 19 | 0.475 |
| Kegg | Hematopoietic_cell_lineage | 88 | 22 | 16 | 0.475 |
| Kegg | Leishmaniasis | 72 | 21 | 19 | 0.471 |
| Reactome | G2_m_transition | 84 | 21 | 19 | 0.471 |
| Reactome | Calmodulin_induced_events | 26 | 17 | 17 | 0.471 |
| Reactome | Cam_pathway | 26 | 17 | 17 | 0.471 |
| Reactome | Centrosome_maturation | 72 | 16 | 19 | 0.47 |
| Reactome | Recruitment_of_mitotic_centrosome_proteins_and_complexes | 72 | 16 | 19 | 0.47 |
| Reactome | Pkb-mediated_events | 28 | 15 | 20 | 0.469 |
| Reactome | Hiv_life_cycle | 113 | 27 | 18 | 0.468 |
| Reactome | Regulation_of_apc_c_activators_between_g1_s_and_early_anaphase | 77 | 19 | 18 | 0.467 |
| Reactome | Biological_oxidations | 139 | 22 | 16 | 0.466 |
| Reactome | Glucagon_signaling_in_metabolic_regulation | 33 | 13 | 17 | 0.464 |
| Reactome | Regulation_of_beta-cell_development | 114 | 17 | 16 | 0.464 |
| Reactome | Regulation_of_insulin_secretion_by_glucagon-like_peptide-1 | 43 | 18 | 18 | 0.463 |
| Reactome | Shc-mediated_cascade | 29 | 9 | 16 | 0.463 |
| Reactome | Ca-dependent_events | 28 | 18 | 17 | 0.461 |
| Reactome | Activation_of_gabab_receptors | 38 | 17 | 17 | 0.459 |
| Reactome | Scf(skp2)-mediated_degradation_of_p27_p21 | 56 | 15 | 16 | 0.459 |
| Reactome | Gaba_b_receptor_activation | 38 | 17 | 17 | 0.459 |
| Reactome | Gluconeogenesis | 31 | 9 | 11 | 0.452 |
| Reactome | Platelet_aggregation_(plug_formation) | 37 | 15 | 17 | 0.452 |
| Reactome | Meiosis | 85 | 15 | 19 | 0.45 |
| Reactome | P53-dependent_g1_dna_damage_response | 57 | 16 | 15 | 0.445 |
| Reactome | P53-dependent_g1_s_dna_damage_checkpoint | 57 | 16 | 15 | 0.445 |
| Pid | Hiv-1_nef_negative_effector_of_fas_and_tnf-alpha | 35 | 17 | 12 | 0.444 |
| Pid | Foxa2_and_foxa3_transcription_factor_networks | 45 | 17 | 17 | 0.44 |
| Reactome | Transport_of_glucose_and_other_sugars_bile_salts_  and_organic_acids_metal_ions_and_amine_compounds | 96 | 29 | 17 | 0.44 |
| Kegg | Oxidative_phosphorylation | 132 | 17 | 14 | 0.436 |
| Reactome | Assembly_of_the_pre-replicative_complex | 67 | 13 | 17 | 0.436 |
| Reactome | Late_phase_of_hiv_life_cycle | 94 | 24 | 18 | 0.436 |
| Reactome | Lipoprotein_metabolism | 29 | 7 | 9 | 0.432 |
| Reactome | Rna_polymerase_ii_transcription | 101 | 27 | 19 | 0.431 |
| Reactome | Insulin_synthesis_and_processing | 135 | 20 | 18 | 0.43 |
| Kegg | Abc_transporters | 44 | 13 | 18 | 0.429 |
| Kegg | Cardiac_muscle_contraction | 77 | 22 | 17 | 0.429 |
| Reactome | Removal_of_licensing_factors_from_origins | 72 | 16 | 15 | 0.426 |
| Reactome | The_citric_acid_(tca)_cycle_and_  respiratory_electron_transport | 130 | 24 | 16 | 0.423 |
| Reactome | Ligand-gated_ion_channel_transport | 25 | 9 | 15 | 0.422 |
| Pid | Calcium_signaling_in_the_cd4+_tcr_pathway | 32 | 17 | 17 | 0.42 |
| Reactome | Regulation_of_apoptosis | 60 | 15 | 15 | 0.417 |
| Kegg | Carbohydrate_digestion_and_absorption | 43 | 13 | 18 | 0.417 |
| Reactome | Synthesis_of_dna | 96 | 18 | 15 | 0.416 |
| Pid | Il8-_and_cxcr2-mediated_signaling_events | 33 | 15 | 14 | 0.415 |
| Kegg | Parkinson's_disease | 130 | 18 | 15 | 0.414 |
| Kegg | Basal_transcription_factors | 35 | 13 | 14 | 0.413 |
| Kegg | Maturity_onset_diabetes_of_the_young | 25 | 10 | 13 | 0.41 |
| Reactome | Chaperonin-mediated_protein_folding | 49 | 11 | 14 | 0.41 |
| Reactome | Mtor_signalling | 27 | 14 | 18 | 0.408 |
| Kegg | Type_i_diabetes_mellitus | 43 | 9 | 10 | 0.407 |
| Reactome | Interferon_alpha_beta_signaling | 64 | 15 | 11 | 0.406 |
| Pid | Amb2_integrin_signaling | 41 | 17 | 17 | 0.4 |
| Reactome | Orc1_removal_from_chromatin | 70 | 15 | 15 | 0.399 |
| Reactome | Switching_of_origins_to_a_post-replicative_state | 70 | 15 | 15 | 0.399 |
| Reactome | Protein_folding | 54 | 11 | 14 | 0.399 |
| Kegg | Malaria | 51 | 12 | 15 | 0.392 |
| Reactome | Amino_acid_transport_across_the_plasma_membrane | 31 | 13 | 13 | 0.388 |
| Pid | Atm_pathway | 34 | 12 | 13 | 0.387 |
| Reactome | Chemokine_receptors_bind_chemokines | 54 | 14 | 12 | 0.387 |
| Reactome | Activation_of_apc_c_and_apc_c_cdc20_  mediated_degradation_of_mitotic_proteins | 70 | 14 | 16 | 0.383 |
| Reactome | Apc_c_cdc20_mediated_degradation_of_mitotic_proteins | 69 | 14 | 16 | 0.383 |
| Reactome | Amine_ligand-binding_receptors | 42 | 14 | 15 | 0.378 |
| Reactome | Muscle_contraction | 49 | 13 | 20 | 0.374 |
| Kegg | Systemic_lupus_erythematosus | 136 | 14 | 14 | 0.373 |
| Kegg | Pyrimidine_metabolism | 99 | 19 | 14 | 0.371 |
| Kegg | Cytosolic_dna-sensing_pathway | 56 | 11 | 13 | 0.369 |
| Reactome | Association_of_tric_cct_with_target_proteins_during_biosynthesis | 29 | 8 | 13 | 0.369 |
| Kegg | O-glycan_biosynthesis | 30 | 9 | 12 | 0.368 |
| Kegg | Glycosphingolipid_biosynthesis_lacto_and_neolacto_series | 26 | 9 | 11 | 0.367 |
| Reactome | Autodegradation_of_cdh1_by_cdh1_apc_c | 60 | 11 | 15 | 0.366 |
| Reactome | Regulation_of_ornithine_decarboxylase_(odc) | 50 | 11 | 14 | 0.366 |
| Kegg | Taste_transduction | 52 | 9 | 12 | 0.366 |
| Reactome | Apc_c_cdc20_mediated_degradation_of_securin | 64 | 12 | 15 | 0.365 |
| Kegg | N-glycan_biosynthesis | 49 | 14 | 14 | 0.364 |
| Reactome | Rna_polymerase_iii_transcription_initiation | 29 | 7 | 13 | 0.361 |
| Reactome | Apc_c_cdh1_mediated_degradation_of_ cdc20_and_other_apc_c_cdh1_  targeted_proteins_in_late_mitosis_early_g1 | 69 | 12 | 15 | 0.36 |
| Reactome | Immunoregulatory_interactions_between_a_lymphoid  _non-lymphoid_cell | 125 | 9 | 6 | 0.354 |
| Reactome | Inhibition_of_insulin_secretion_by_adrenaline_noradrenaline | 29 | 13 | 13 | 0.354 |
| Kegg | Selenoamino_acid_metabolism | 26 | 12 | 15 | 0.354 |
| Reactome | Transcription_of_the_hiv_genome | 61 | 14 | 16 | 0.353 |
| Reactome | Destabilization_of_mrna_by_auf1_(hnrnp_d0) | 54 | 11 | 10 | 0.348 |
| Pid | Atr_signaling_pathway | 39 | 12 | 15 | 0.346 |
| Reactome | Generation_of_second_messenger_molecules | 42 | 14 | 13 | 0.346 |
| Reactome | Meiotic_synapsis | 57 | 10 | 17 | 0.346 |
| Reactome | Signal_amplification | 31 | 10 | 12 | 0.345 |
| Reactome | G-protein_beta_gamma_signalling | 28 | 9 | 12 | 0.345 |
| Reactome | Negative_regulators_of_rig-i_mda5_signaling | 33 | 14 | 13 | 0.344 |
| Kegg | Ether_lipid_metabolism | 35 | 8 | 13 | 0.343 |
| Kegg | Peroxisome | 79 | 17 | 16 | 0.343 |
| Reactome | Translation | 119 | 13 | 15 | 0.34 |
| Kegg | Allograft_rejection | 37 | 7 | 9 | 0.336 |
| Reactome | Rna_polymerase_ii_pre-transcription_events | 58 | 12 | 15 | 0.335 |
| Kegg | Complement_and_coagulation_cascades | 69 | 12 | 13 | 0.332 |
| Reactome | Vpu_mediated_degradation_of_cd4 | 52 | 10 | 11 | 0.329 |
| Reactome | Scf-beta-trcp_mediated_degradation_of_emi1 | 54 | 11 | 11 | 0.329 |
| Reactome | Autodegradation_of_the_e3_ubiquitin_ligase_cop1 | 51 | 11 | 13 | 0.327 |
| Reactome | Transport_of_mature_mrnas_derived_from_intronless_transcripts | 36 | 14 | 13 | 0.327 |
| Kegg | Graft-versus-host_disease | 41 | 7 | 9 | 0.326 |
| Reactome | Abc-family_proteins_mediated_transport | 38 | 10 | 16 | 0.326 |
| Reactome | Stabilization_of_p53 | 52 | 11 | 13 | 0.326 |
| Reactome | Kinesins | 27 | 11 | 12 | 0.325 |
| Reactome | Transport_of_mature_transcript_to_cytoplasm | 55 | 18 | 14 | 0.324 |
| Reactome | Pyruvate_metabolism_and_citric_acid_(tca)_cycle | 40 | 16 | 12 | 0.317 |
| Reactome | Glucose_transport | 40 | 13 | 14 | 0.315 |
| Reactome | Cdk-mediated_phosphorylation_and_removal_of_cdc6 | 50 | 10 | 12 | 0.315 |
| Reactome | P53-independent_dna_damage_response | 52 | 10 | 11 | 0.313 |
| Reactome | P53-independent_g1_s_dna_damage_checkpoint | 52 | 10 | 11 | 0.313 |
| Reactome | Ubiquitin_mediated_degradation_of_phosphorylated_cdc25a | 52 | 10 | 11 | 0.313 |
| Reactome | Antigen_processing-cross_presentation | 75 | 11 | 13 | 0.312 |
| Pid | Il12-mediated_signaling_events | 65 | 19 | 17 | 0.311 |
| Reactome | Interactions_of_vpr_with_host_cellular_proteins | 40 | 14 | 13 | 0.31 |
| Reactome | Hexose_transport | 42 | 13 | 14 | 0.309 |
| Kegg | Cysteine_and_methionine_metabolism | 36 | 13 | 12 | 0.308 |
| Reactome | Eukaryotic_translation_initiation | 112 | 12 | 14 | 0.306 |
| Reactome | Ubiquitin-dependent_degradation_of_cyclin_d | 50 | 9 | 10 | 0.306 |
| Reactome | Cap-dependent_translation_initiation | 112 | 12 | 14 | 0.306 |
| Reactome | Nitric_oxide_stimulates_guanylate_cyclase | 28 | 10 | 12 | 0.306 |
| Reactome | Transport_of_ribonucleoproteins_into_the_host_nucleus | 33 | 12 | 13 | 0.306 |
| Reactome | Ubiquitin-dependent_degradation_of_cyclin_d1 | 50 | 9 | 10 | 0.306 |
| Reactome | Transport_of_mature_mrna_derived_from_an_intronless_transcript | 35 | 13 | 13 | 0.305 |
| Kegg | Fructose_and_mannose_metabolism | 34 | 10 | 12 | 0.304 |
| Reactome | Vpr-mediated_nuclear_import_of_pics | 37 | 12 | 13 | 0.304 |
| Reactome | Dna_repair | 108 | 18 | 13 | 0.303 |
| Reactome | Interleukin_receptor_shc_signaling | 28 | 9 | 12 | 0.303 |
| Reactome | Metabolism_of_non-coding_rna | 49 | 18 | 14 | 0.301 |
| Kegg | Autoimmune_thyroid_disease | 52 | 7 | 9 | 0.301 |
| Reactome | Snrnp_assembly | 49 | 18 | 14 | 0.301 |
| Reactome | Nonsense-mediated_decay | 106 | 10 | 13 | 0.3 |
| Reactome | Nonsense_mediated_decay_enhanced_by_the_exon junction_complex | 106 | 10 | 13 | 0.3 |
| Reactome | Tight_junction_interactions | 30 | 10 | 11 | 0.3 |
| Reactome | Cdt1_association_with_the_cdc6_orc_origin_complex | 58 | 10 | 11 | 0.299 |
| Reactome | Basigin_interactions | 25 | 10 | 13 | 0.295 |
| Kegg | Ppar_signaling_pathway | 70 | 17 | 11 | 0.295 |
| Reactome | Gtp_hydrolysis_and_joining_of_the_60s_ribosomal_subunit | 105 | 12 | 14 | 0.295 |
| Kegg | Rna_polymerase | 29 | 5 | 12 | 0.294 |
| Reactome | Regulation_of_activated_pak-2p34_by_proteasome_mediated_degradation | 49 | 9 | 11 | 0.293 |
| Reactome | Inwardly_rectifying_k+_channels | 31 | 10 | 15 | 0.292 |
| Reactome | Metal_ion_slc_transporters | 25 | 12 | 9 | 0.29 |
| Reactome | G-protein_activation | 28 | 10 | 12 | 0.289 |
| Reactome | Regulation_of_gene_expression_in_beta_cells | 102 | 10 | 11 | 0.288 |
| Pid | Alpha-synuclein_signaling | 33 | 8 | 15 | 0.283 |
| Reactome | Activation_of_kainate_receptors_upon_glutamate_binding | 30 | 9 | 15 | 0.283 |
| Reactome | Transport_of_the_slbp_dependant_mature_mrna | 33 | 13 | 12 | 0.281 |
| Kegg | Sphingolipid_metabolism | 40 | 11 | 11 | 0.281 |
| Reactome | Vif-mediated_degradation_of_apobec3g | 54 | 9 | 11 | 0.28 |
| Kegg | Antigen_processing_and_presentation | 76 | 13 | 14 | 0.278 |
| Pid | Il23-mediated_signaling_events | 37 | 9 | 11 | 0.277 |
| Reactome | Cross-presentation_of_soluble_exogenous_antigens_(endosomes) | 49 | 7 | 9 | 0.276 |
| Pid | Alpha9_beta1_integrin_signaling_events | 25 | 9 | 13 | 0.276 |
| Reactome | E2f_mediated_regulation_of_dna_replication | 33 | 11 | 8 | 0.274 |
| Kegg | Glycerolipid_metabolism | 49 | 11 | 14 | 0.271 |
| Reactome | Transport_of_mature_mrna_derived_from_an_intron-containing_transcript | 51 | 16 | 13 | 0.27 |
| Reactome | Glycolysis | 27 | 7 | 8 | 0.269 |
| Reactome | Er-phagosome_pathway | 64 | 9 | 10 | 0.267 |
| Reactome | Iron_uptake_and_transport | 37 | 12 | 13 | 0.265 |
| Reactome | Activation_of_g_protein_gated_potassium_channels | 25 | 8 | 13 | 0.265 |
| Reactome | G_protein_gated_potassium_channels | 25 | 8 | 13 | 0.265 |
| Reactome | Inhibition_of_voltage_gated_ca2+_channels_via_gbeta_gamma_subunits | 25 | 8 | 13 | 0.265 |
| Pid | Bard1_signaling_events | 29 | 11 | 11 | 0.265 |
| Reactome | Transport_of_the_slbp_independent_mature_mrna | 32 | 12 | 12 | 0.261 |
| Reactome | L13a-mediated_translational_silencing_of_ceruloplasmin_expression | 104 | 11 | 13 | 0.26 |
| Reactome | Traf6_mediated_irf7_activation | 30 | 7 | 13 | 0.257 |
| Reactome | Endosomal_sorting_complex_required_for_transport_(escrt) | 28 | 10 | 12 | 0.255 |
| Reactome | Ribosomal_scanning_and_start_codon_recognition | 55 | 11 | 13 | 0.249 |
| Reactome | Nep_ns2_interacts_with_the_cellular_export_machinery | 35 | 11 | 11 | 0.249 |
| Reactome | Phase_ii_conjugation | 71 | 11 | 13 | 0.248 |
| Reactome | Thrombin_signalling_through_proteinase_activated_receptors_(pars) | 32 | 10 | 8 | 0.248 |
| Reactome | Export_of_viral_ribonucleoproteins_from_nucleus | 36 | 11 | 11 | 0.247 |
| Reactome | Influenza_viral_rna_transcription_and_replication | 108 | 5 | 8 | 0.247 |
| Kegg | Citrate_cycle_(tca_cycle) | 31 | 10 | 10 | 0.245 |
| Reactome | Regulation_of_glucokinase_by_glucokinase_regulatory_protein | 29 | 10 | 11 | 0.244 |
| Reactome | G_beta_gamma_signalling_through_pi3kgamma | 25 | 8 | 9 | 0.243 |
| Pid | Il27-mediated_signaling_events | 26 | 6 | 11 | 0.239 |
| Reactome | Nuclear_import_of_rev_protein | 31 | 10 | 11 | 0.239 |
| Kegg | Proteasome | 44 | 6 | 7 | 0.237 |
| Kegg | Amino_sugar_and_nucleotide_sugar_metabolism | 47 | 12 | 11 | 0.237 |
| Reactome | Rna_polymerase_ii_hiv-1_promoter_escape | 39 | 9 | 10 | 0.236 |
| Reactome | Rna_polymerase_ii_transcription_pre-initiation_and_promoter_opening | 39 | 9 | 10 | 0.236 |
| Reactome | Rna_polymerase_ii_promoter_escape | 39 | 9 | 10 | 0.236 |
| Reactome | Hiv-1_transcription_initiation | 39 | 9 | 10 | 0.236 |
| Reactome | Rna_polymerase_ii_transcription_initiation | 39 | 9 | 10 | 0.236 |
| Reactome | Rna_polymerase_ii_transcription_initiation_and_promoter_clearance | 39 | 9 | 10 | 0.236 |
| Reactome | Rev-mediated_nuclear_export_of_hiv-1_rna | 33 | 10 | 11 | 0.235 |
| Reactome | Striated_muscle_contraction | 31 | 7 | 15 | 0.233 |
| Reactome | Interactions_of_rev_with_host_cellular_proteins | 35 | 10 | 11 | 0.232 |
| Reactome | Glucagon-type_ligand_receptors | 33 | 7 | 10 | 0.231 |
| Reactome | Nonsense_mediated_decay_independent_  of_the_exon_junction_complex | 89 | 4 | 8 | 0.227 |
| Kegg | Nucleotide_excision_repair | 44 | 8 | 11 | 0.226 |
| Reactome | Metabolism_of_steroid_hormones_  and_vitamins_a_and_d | 36 | 5 | 9 | 0.224 |
| Reactome | Metabolism_of_nucleotides | 70 | 14 | 11 | 0.222 |
| Reactome | Regulation_of_ifna_signaling | 25 | 6 | 7 | 0.221 |
| Reactome | Metabolism_of_water-soluble_  vitamins_and_cofactors | 51 | 16 | 14 | 0.221 |
| Reactome | Metabolism_of_vitamins_and_cofactors | 51 | 16 | 14 | 0.221 |
| Kegg | Glycine_serine_and_threonine_metabolism | 32 | 6 | 8 | 0.219 |
| Kegg | Arginine_and_proline_metabolism | 54 | 9 | 11 | 0.219 |
| Reactome | Respiratory_atp_synthesis_ _and_heat_production_by_uncoupling_proteins | 94 | 9 | 7 | 0.217 |
| Reactome | G2_m_checkpoints | 43 | 8 | 9 | 0.217 |
| Kegg | Tryptophan_metabolism | 42 | 7 | 6 | 0.216 |
| Kegg | Glycolysis_gluconeogenesis | 65 | 10 | 6 | 0.215 |
| Reactome | Gap_junction_trafficking_and_regulation | 30 | 9 | 11 | 0.213 |
| Reactome | Translation_initiation_complex_formation | 55 | 10 | 12 | 0.21 |
| Reactome | Activation_of_the_mrna_upon_binding_of_the_cap-binding_complex_and_eifs_and_subsequent_binding_to_43s | 56 | 10 | 12 | 0.21 |
| Kegg | Regulation_of_autophagy | 34 | 9 | 8 | 0.21 |
| Pid | Plk1_signaling_events | 44 | 12 | 10 | 0.208 |
| Kegg | Fatty_acid_metabolism | 43 | 8 | 8 | 0.207 |
| Reactome | Formation_of_hiv-1_elongation _complex_in_the_absence_of_hiv-1_tat | 42 | 6 | 11 | 0.203 |
| Reactome | Rna_polymerase_ii_transcription_elongation | 42 | 6 | 11 | 0.203 |
| Reactome | Formation_of_rna_pol_ii_elongation_complex | 42 | 6 | 11 | 0.203 |
| Reactome | Adp_signalling_through_p2y_purinoceptor_1 | 25 | 6 | 9 | 0.202 |
| Reactome | Eukaryotic_translation_termination | 84 | 3 | 7 | 0.201 |
| Kegg | Valine_leucine_and_isoleucine_degradation | 44 | 10 | 11 | 0.201 |
| Reactome | Viral_mrna_translation | 92 | 4 | 7 | 0.201 |
| Kegg | Glutathione_metabolism | 50 | 11 | 12 | 0.199 |
| Kegg | Butanoate_metabolism | 30 | 8 | 9 | 0.192 |
| Reactome | Post-translational_modification_synthesis_of_gpi-anchored_proteins | 26 | 7 | 12 | 0.192 |
| Kegg | Pyruvate_metabolism | 41 | 8 | 7 | 0.187 |
| Reactome | Transport_of_vitamins_nucleosides_and_related_molecules | 31 | 8 | 7 | 0.187 |
| Reactome | Prefoldin_mediated_transfer_of_substrate_to_cct_tric | 27 | 5 | 7 | 0.184 |
| Kegg | Starch_and_sucrose_metabolism | 53 | 6 | 8 | 0.184 |
| Kegg | Tyrosine_metabolism | 41 | 5 | 6 | 0.182 |
| Reactome | Cooperation_of_prefoldin_and_tric_cct_in_actin_and_tubulin_folding | 28 | 5 | 7 | 0.182 |
| Kegg | Alanine_aspartate_and_glutamate_metabolism | 32 | 6 | 7 | 0.181 |
| Reactome | Phase_1_functionalization_of_compounds | 69 | 11 | 8 | 0.18 |
| Pid | Fanconi_anemia_pathway | 48 | 7 | 9 | 0.179 |
| Reactome | Rna_polymerase_ii_transcription_termination | 43 | 13 | 10 | 0.176 |
| Reactome | Cleavage_of_growing_transcript_in_the_termination_region | 43 | 13 | 10 | 0.176 |
| Reactome | Nucleotide_excision_repair | 49 | 9 | 10 | 0.173 |
| Reactome | Transferrin_endocytosis_and_recycling | 27 | 9 | 10 | 0.167 |
| Kegg | Arachidonic_acid_metabolism | 57 | 8 | 8 | 0.166 |
| Kegg | Galactose_metabolism | 26 | 7 | 8 | 0.165 |
| Kegg | Drug_metabolism_cytochrome_p450 | 73 | 5 | 5 | 0.164 |
| Reactome | Formation_of_the_ternary_complex_and_subsequently_the_43s_complex | 48 | 5 | 10 | 0.163 |
| Pid | Endogenous_tlr_signaling | 25 | 6 | 6 | 0.162 |
| Reactome | Phosphorylation_of_cd3_and_tcr_zeta_chains | 31 | 6 | 6 | 0.16 |
| Reactome | Respiratory_electron_transport | 76 | 7 | 4 | 0.157 |
| Reactome | Activation_of_atr_in_response_to_replication_stress | 37 | 6 | 7 | 0.156 |
| Reactome | Amyloids | 51 | 3 | 6 | 0.155 |
| Kegg | Retinol_metabolism | 65 | 7 | 6 | 0.155 |
| GO MF | Antioxidant_activity | 40 | 9 | 8 | 0.155 |
| Reactome | Global_genomic_ner_(gg-ner) | 33 | 6 | 8 | 0.153 |
| Kegg | Staphylococcus_aureus_infection | 55 | 7 | 9 | 0.15 |
| Reactome | Meiotic_recombination | 54 | 6 | 6 | 0.146 |
| Kegg | Pentose_phosphate_pathway | 26 | 4 | 2 | 0.146 |
| Reactome | Pausing_and_recovery_of_hiv-1_elongation | 31 | 4 | 9 | 0.145 |
| Reactome | Hiv-1_elongation_arrest_and_recovery | 31 | 4 | 9 | 0.145 |
| Reactome | Rna_polymerase_i_transcription | 55 | 5 | 8 | 0.145 |
| Reactome | Mrna_3'-end_processing | 34 | 9 | 9 | 0.143 |
| Reactome | Rna_polymerase_i_promoter_clearance | 53 | 5 | 8 | 0.143 |
| Kegg | Glycosylphosphatidylinositol(gpi)-anchor_biosynthesis | 25 | 7 | 9 | 0.142 |
| Reactome | Activation_of_the_pre-replicative_complex | 30 | 5 | 5 | 0.141 |
| Kegg | Steroid_hormone_biosynthesis | 56 | 4 | 7 | 0.141 |
| Reactome | Rna_pol_ii_ctd_phosphorylation_and_interaction_with_ce | 26 | 5 | 8 | 0.138 |
| Reactome | Rna_pol_ii_ctd_phosphorylation_and_interaction_with_ce | 26 | 5 | 8 | 0.138 |
| Reactome | Transcription-coupled_ner_(tc-ner) | 44 | 8 | 9 | 0.137 |
| Reactome | Mrna_capping | 28 | 5 | 8 | 0.134 |
| Kegg | Collecting_duct_acid_secretion | 27 | 6 | 8 | 0.133 |
| Reactome | Insulin_receptor_recycling | 25 | 8 | 8 | 0.133 |
| Reactome | Formation_of_a_pool_of_free_40s_subunits | 94 | 4 | 8 | 0.13 |
| Reactome | Gap_junction_trafficking | 28 | 7 | 7 | 0.128 |
| Reactome | Chromosome_maintenance | 79 | 6 | 6 | 0.126 |
| Kegg | Linoleic_acid_metabolism | 29 | 4 | 5 | 0.121 |
| Reactome | Olfactory_signaling_pathway | 377 | 1 | 1 | 0.121 |
| Reactome | Cytochrome_p450_arranged_by_substrate_type | 50 | 6 | 5 | 0.12 |
| Reactome | Formation_of_fibrin_clot_(clotting_cascade) | 32 | 3 | 6 | 0.119 |
| Reactome | Formation_of_hiv-1_elongation_complex_containing_hiv-1_tat | 42 | 5 | 8 | 0.114 |
| Reactome | Hiv-1_transcription_elongation | 42 | 5 | 8 | 0.114 |
| Kegg | Propanoate_metabolism | 32 | 4 | 5 | 0.114 |
| Reactome | Tat-mediated_elongation_of_the_hiv-1_transcript | 42 | 5 | 8 | 0.114 |
| Reactome | Formation_of_transcription-coupled_ner_(tc-ner)_repair_complex | 28 | 5 | 7 | 0.113 |
| Kegg | Primary_immunodeficiency | 35 | 7 | 6 | 0.113 |
| Reactome | Dual_incision_reaction_in_tc-ner | 28 | 5 | 7 | 0.113 |
| Reactome | Purine_metabolism | 32 | 4 | 5 | 0.103 |
| Kegg | Histidine_metabolism | 29 | 4 | 4 | 0.1 |
| Reactome | Mrna_splicing_minor_pathway | 42 | 6 | 6 | 0.098 |
| Reactome | Formation_of_the_hiv-1_early_elongation_complex | 32 | 3 | 6 | 0.093 |
| Reactome | Formation_of_the_early_elongation_complex | 32 | 3 | 6 | 0.093 |
| Reactome | Glutathione_conjugation | 25 | 3 | 4 | 0.086 |
| Kegg | Base_excision_repair | 33 | 5 | 5 | 0.081 |
