# Supplementary material for: Chronic binge alcohol administration dysregulates global regulatory gene networks associated with skeletal muscle wasting in simian immunodeficiency virus-infected macaques
Source: BMC Genomics. 2015 Dec 23;16:1097. doi: 10.1186/s12864-015-2329-z (PMC4690320; doi:10.1186/s12864-015-2329-z)
Supplement: Additional file 4: Table S4. — CBA-dependent alterations in microRNA expression at end-stage SIV infection. (DOCX 24 kb) [file 12864_2015_2329_MOESM4_ESM.docx]

**Additional file 4: Table S4. CBA-dependent alterations in microRNA expression at end-stage SIV infection**

| **Downregulated miRNAs** | | | |
| --- | --- | --- | --- |
| **miR** | **Target gene** | **Gene Function** | **Fold Change** |
| 1281 | *GCLM* | *Rate limiting enzyme of glutathione synthesis* | -2.46 |
| 933 | *BDNF*  *COL12A1*  *MEF2A* | *Neurotrophic factor – promotes satellite cell regeneration*  *Collagen 12 alpha 1*  *Myogenic factor – promotes myogenesis* | -2.21 |
| 1180 | *MYO10*  *VEGFB* | *Actin-based molecular motor*  *Regulates the formation of blood vessels* | -2.14 |
| 944 | *KCNH2* | *Voltage gated potassium channel* | -2.02 |
| 557-5p | *ADAM17*  *CAMK4* | *Metallopeptidase –activates receptor ligands*  *Multifunctional Ca+2-dependent kinase* | -1.98 |
| 940 | *CADM2*  *NEDD4*  *SCN4A*  *STMN2* | *Cell adhesion molecule – synapse formation*  *E3 ubiquitin ligase –degradation of growth receptors*  *Voltage-gated sodium channel*  *Microtubule stability and controls neurite growth* | -1.86 |
| 766 | *ACCN2*  *CADM2*  *DES*  *DAG1* | *Cation channel – influences intracellular Ca+2 concentrations*  *Cell adhesion molecule – synapse formation*  *Desmin – connect myofibrils; important for muscle structure*  *Dystroglycan –important for muscle structure* | -1.80 |
| 346 | EFEMP2  LIF  ZFP36 | Ca+2 ECM protein connective tissue formation  Induction of neuronal cell differentiation  RNA binding – post-transcriptional degradation of TNF | -1.64 |
| 501 | LAMTOR5 | Amino acid sensor to activate mTORC1 | -1.63 |
| 198 | CCNT1  MET  MYB  NTRK3 | Cyclin T1 – transcription elongation factor  Tyr kinase receptor –proliferation and differentiation of myoblasts  Proliferation transcription factor – inhibits differentiation  Tyr kinase receptor – promotes neuronal differentiation | -1.62 |
| 1229 | *EIF4G2* | *Eukaryotic initiation factor* | -1.48 |
| **Upregulated miRNAs** | | | |
| 34a | AXL  BCL2  CCND1  CCNE2  CD44  CDK4  CDK6  E2F1  E2F3  EPHA5  FOSL1  HNF4A  JAG1  MAGEA2  MAGEA3  MAGEA6  MAGEA12  MAP2K1  MAP3K9  MET  MYB  MYC  NANOG  NOTCH1  NOTCH2  SIRT1  SOX2  STX1A SYT1 | Multifunctional receptor – Akt activation  Inhibits apoptosis  Cyclin D1 – G1 progression  Cyclin E2 – important for G1/S transition  Cell surface glycoprotein involved in cell adhesion  Cyclin dependent kinase – G1 progression  Cyclin dependent kinase – G1 progression, G1/S transition  Cell cycle regulatory transcription factor  Cell cycle regulatory transcription factor  Neuronal receptor – involved in synaptic plasticity  Fos family member – multifunctional  Hepatocyte Nuclear Factor 4 – liver-specific gene  Notch ligand – inhibits neuronal and myoblast differentiation  Reduces p53 transactivation  Propose to enhance ubiquitin ligase activity  Proposed to enhance ubiquitin ligase activity  Unknown function – may promote proliferation  Multifunctional MAPK signaling molecule  Multifunctional MAPK signaling molecule  Tyr kinase receptor –proliferation and differentiation of myoblasts  Proliferation transcription factor – inhibits differentiation  Multifunctional Pro-proliferative transcription factor  Involved in cell proliferation and self-renewal  Represses neuronal and myogenic differentiation  Represses neuronal and myogenic differentiation  Protein deacetylase – multiple functions  Functions as a switch in neural development  Ion channel and Ca+2 regulation of synaptic exocytosis  Ca+2-dependent neurotransmitter release at synapses | +5.29 |
| 10b | BCL2L11  CDKN2A  CDKN1A  HOXD10  NCOR2  NF1  PPARA  TFAP2C | Inhibits neuronal apoptosis  Promotes G1 and G2 cell cycle arrest  Promotes G1 and G2 cell cycle arrest  Promotes musculature nerve fiber formation  Nuclear receptor co-repressor – transcriptional silencing  Negative regulator of Ras– required for muscle development  Regulates fatty acid synthesis and lipoprotein assembly  General developmental transcription factor | +4.14 |
| 146b-5p | MMP16 | Matrix metallopeptidase – breakdown of the ECM | +3.43 |
| 20 | ARID4B  BAMBI  BMPR2  CCND1  CDKN1A  CRIM1  E2F1  ESR1  HIF1A  MET  MYLIP  PPARG  TGFBR2 | Multifunctional co-repressor – chromatin remodeling complex  Negatively regulates TGFbeta signaling  BMP receptor – bone formation  Cyclin D1 – promote G1 progression  Promotes G1 and G2 cell cycle arrest  Involved in CNS development and motor neuron differentiation  Cell cycle regulatory transcription factor  Estrogen receptor  Hypoxia-induced transcription factor/increase oxygen delivery  Tyr kinase receptor –proliferation and differentiation of myoblasts  E3 ubiquitin ligase – degradation of myosin regulatory LC  Nuclear receptor – mediates adipocyte differentiation  TGFbeta receptor – promotes cellular proliferation | +3.33 |
| 146a | BRCA1  CCNA2  EGFR  ERBB4  FAS  PA2G4  SMAD4 | Integral in DNA repair response  Cyclin A2 – promotes G1/S and G2/M transition  Growth factor receptor – promotes cellular proliferation  Multifunctional growth factor receptor – CNS development  Cell surface receptor that promotes apoptosis  Implicated in growth inhibition and induction of differentiation  Transcription factor required for TGFbeta signaling | +3.32 |
| 31 | DACT3  DKK1  DMD  FZD3  HIF1AN  ITGA5  MMP16  RDX  RHOARD | Negative regulator of Wnt/beta catenin signaling  Secreted protein that antagonizes Wnt signaling  Anchors ECM to actin and forms at neuromuscular junctions  Frizzled – receptor for Wnt proteins  Hypoxia-induced transcription factor to increase oxygen delivery  Ca+2 dependent cell adhesion molecule  Matrix metallopeptidase – breakdown of the ECM  Cytoskeletal protein – links actin to plasma membrane  Link receptors to focal adhesion and actin stress fibers | +3.22 |
| 188 | UBE21 | A SUMOylation enzyme – SUMOlates proteins | +2.97 |
| 17-3p | VIM | Cytoskeletal protein important for maintaining cellular integrity | +2.81 |
| 660 | *CDH13*  *JPH1* | *Ca+2-dependent adhesion– negative reg. of neural growth*  *Forms junctional membrane complexes – construction of muscle* | +2.47 |
| 335 | BCL2L2  MAPK1  RB1  RUNX2 | Inhibits apoptosis  Multifunctional MAPK signaling molecule  Central inhibitor of cell cycle progression  Osteoblast differentiation and skeletal morphogenesis | +2.17 |
| 25 | BCL2L11  CDH1  CDKN1C  EZH2  MDM2  TP53 | Inhibits neuronal apoptosis  E-cadherin – Ca+2-dependent adhesion molecule  Promotes cell cycle arrest in G1 phase  Histone methyl transferase –repressed state in neurons  E3 ubiquitin ligase that target p53 for degradation  Multifunction transcription factor – tumor suppressor | +2.00 |
| 26b | ABCA1  ARL4C  CCNE1  CDK6  EPHA2  GATA4 | Cholesterol efflux pump  Part of ABC cholesterol efflux pump  Cyclin E1 – important for G1/S transition  Promotes G1 phase progression  Neuronal receptor – involved in neuronal development  Transcription factor that plays key role in myocyte development | +1.98 |
| 671-5p | *HDAC5*  *MBD2* | *Histone deacetylase – binds and represses MEF2 proteins*  *Methylated DNA binder – transcriptional repressor* | +1.85 |
| 18 | ATM  CDK19  CTGF  DICER1  ESR1  KRAS  Myc  NEDD9  NR3C1  PRMT5  PTEN | Activates check point signaling in response to DNA damage  Part of mediator complex for general transcriptional activation  Mediates divalent cation-dependent cell adhesion  Involved in processing of pri-miRNA to pre-miRNA  Estrogen receptor  G-protein receptor involved in growth  Multifunctional Pro-proliferative transcription factor  Focal adhesion protein – mediates signaling  Glucocorticoid receptor – multiple functions  Arginine methyl transferase – multifunctional  Tumor suppressor – inhibits cell growth and promote apoptosis | +1.81 |
| 374b | *MEF2D*  *MMP14* | *Myogenic factor – promotes myogenesis*  *Matrix metallopeptidase –protease involved in ECM degradation* | +1.65 |
| 10a | EPHA4  HOXA1  MAP3K7  NCOR2 | Involved in neuromuscular circuit development  Important for embryonic patterning  Mediates TGFbeta and BMP signaling  Nuclear receptor co-repressor –silencing through chromatin | +1.63 |
| 668 | *MTMR3* | *A lipid phosphatase* | +1.56 |
| 98 | E2F1  E2F2  HMGA2  MYC  TUSC2 | Cell cycle regulatory transcription factor  Cell cycle regulatory transcription factor  Facilitates G2/M transition in cell cycle  Multifunctional Pro-proliferative transcription factor  Possible involvement in G1 arrest | +1.54 |
| 148b | MCL1 | Anti-apoptotic – maintain viability | +1.54 |
| 615-3p | LCOR | Co-repressor of nuclear receptors | +1.53 |
| 649 | *DPYD* | *Rate limiting enzyme – biosynthesis of uracil and thymidine* | +1.50 |

*microRNAs are listed in descending magnitude of fold-change vs. SUC/SIV. Genes listed in regular font are validated gene targets, as determined from miRTarBase. Genes listed in italics are highly predicted targets, as determined through the TargetScan database, based on their predicted efficacy of targeting (context score ≥ 85%) or their probability of conserved targeting (P_CT_ ≥ 0.8), as previously described [*[*18-20*](#_ENREF_18)*].*
