# Supplementary material for: Chronic binge alcohol administration dysregulates global regulatory gene networks associated with skeletal muscle wasting in simian immunodeficiency virus-infected macaques
Source: BMC Genomics. 2015 Dec 23;16:1097. doi: 10.1186/s12864-015-2329-z (PMC4690320; doi:10.1186/s12864-015-2329-z)
Supplement: Additional file 5: Table S5. — CBA-dependent alterations in promoter methylation at end-stage SIV infection. (DOCX 20 kb) [file 12864_2015_2329_MOESM5_ESM.docx]

**Additional file 5: Table S5: CBA-dependent alterations in promoter methylation at end-stage SIV infection**

| **Downregulated promoter methylation** | | |
| --- | --- | --- |
| *General Cellular Functions* | | **Fold Change** |
| EIF3S2 | Translation initiation complex protein | -1.49 |
| SMARCAL1 | ATP-dependent annealing helicase – re-anneals unwound DNA | -1.72 |
| WDR45L | Important for autophagy | -1.57 |
|  | | |
| *Actin/Microtubule Proteins* | |  |
| CAPZB | Caps F-actin – regulate actin polymerization (muscle contraction) | -1.52 |
| *HSPB1 | Heat shock protein – stress resistance and actin organization | -1.56 |
|  | | |
| *Glycolysis/Energy Metabolism* | |  |
| GPT | Intermediary role between glucose and amino acid metabolism | -2.14 |
| *PDK4 | Ser/Thr kinase – glucose and fatty acid metabolism | -3.96 |
|  | | |
| *Cellular Adhesion* | |  |
| *RND3 | Negative regulator of cytoskeletal organization and adhesion | -1.48 |
|  | | |
| *Cell Growth/Cell Cycle Regulation* | |  |
| *LATS2 | Inhibits proliferation/promotes apoptosis | -7.29 |
|  | | |
| *Enzymes/Enzymatic Activity* | |  |
| *PAPPA | Metalloproteinase – cleaves IGFBP4 and 5 (increases free IGF) | -2.08 |
|  | | |
| *Extracellular Matrix* | |  |
| *UGDH | Biosynthesis of glycosaminoglycans | -3.06 |
|  | | |
| *Neuronal Function* | |  |
| SOX8 | Transcription factor in CNS development | -1.98 |
| Upregulated promoter methylation | | |
| *General Cellular Functions* | | **Fold change** |
| CLTA | Clathirin light chain - major component of vesicles | +1.64 |
| COPE | Transport of proteins from Golgi to ER | +2.61 |
| CRKRS | CDK12 - required for RNA splicing and transcription elongation | +1.62 |
| CSE1L | Mediates export of proteins from nucleus to cytoplasm | +2.54 |
| *EEF2K | Ca+2-dependent kinase for protein synthesis | +4.92 |
| EXOSC4 | Involved in RNA processing | +6.65 |
| HNRPU | RNA binding protein – promotes stabilization of Myc mRNA | +2.22 |
| KARS | Lysyl tRNA synthetase | +1.59 |
| LSM4 | Involved in mRNA splicing | +1.79 |
| MYO18A | Involved with Golgi membrane trafficking | +2.08 |
| *NIPSNAP3B | Possible role in vesicular transport | +2.54 |
| NME7 | Synthesis of nucleoside triphosphates | +2.31 |
| NOL5A | Important for the biogenesis of the 60S ribosomal subunit | +1.61 |
| *OSBP | Intracellular protein - transports sterols from lysosomes to nucleus | +1.70 |
| PRPF8 | Required for mRNA splicing | +1.87 |
| RABEP1 | Membrane trafficking of endosome recycling | +4.62 |
| RPL26 | Ribosomal protein in the 60S subunit | +2.25 |
| RPLP2 | Ribosomal phosphoprotein - translation | +5.18 |
| RPP21 | Promotes generation of mature tRNA | +3.18 |
| SCYL1 | Transcriptional activator - activates DNA Pol beta | +8.59 |
| SERBP1 | Involved in mRNA stability | +2.31 |
| SNAPC4 | Required for transcription of RNA Pol II | +2.50 |
| SUB1 | Coactivator that facilitates TAF binding to RNA Pol II | +4.77 |
| TAF7 | A component of the TFIID general transcription machinery | +1.67 |
| TCEAL8 | Involved with transcriptional regulation | +1.84 |
| TTC15 | ER to Golgi trafficking | +2.17 |
|  | | |
| *Neuronal Function* | |  |
| *BTBD3 | Regulator of dendritic cell orientation | +2.98 |
| CHGA | Found in secretory vesicles of the neuron | +1.83 |
| *GLRB | Glycine receptor - neurotransmitter ion channel | +4.54 |
| *GPC4 | Cell surface glycoprotein involved in CNS development | +2.25 |
| GPR37 | G-protein coupled receptor - receptor for neuro-protection | +12.43 |
| *IRX3 | Transcription factor involved in neuron development | +2.42 |
| NARG1 | N-acetyltransferase important for neuronal growth | +4.47 |
| NLGN1 | Cell surface protein involved in synapse function | +1.82 |
| RUSC1 | Signaling adaptor in neuronal differentiation | +7.21 |
| SOX8 | Transcription factor in CNS development | +12.73 |
| VIPR1 | G-protein coupled receptor for neuronal survival | +9.83 |
|  | | |
| *Enzymes/Enzymatic Activity* | |  |
| B4GALT3 | Involved with the synthesis of complex N-linked oligosaccharides | +1.64 |
| MAOA | Oxidizes monoamines such as dopamine, serotonin, and adrenalin | +3.62 |
| PIN1 | Proline isomerase – involved in mitosis | +1.82 |
| PIP5K1B | Biosynthesis of PIP - secondary messenger | +9.13 |
| PRMT1 | Arg methyltransferase - targets include histones and ESR1 | +1.59 |
| PRMT7 | Arginine methyltransferase - di-methylation of Arg on histones | +1.49 |
| RUVBL1 | Part of histone acetyltransferase complex | +5.79 |
| TDG | Enzyme involved in DNA demethylation | +3.54 |
|  | | |
| *Signaling Pathways* | |  |
| ATXN1 | Represses Notch signaling - involved in neurodegeneration | +2.20 |
| BAMBI | Negatively regulates TGFbeta signaling | +4.66 |
| BRD7 | Chromatin remodeler - activates Wnt and ESR1 signaling | +1.59 |
| FRS3 | Substrate for FGF receptor - FGF signaling | +2.54 |
| IGF2BP2 | Binds IGF2 3'-UTR and regulates translation of IGF2 | +2.37 |
| PHIP | IGF signaling and cytoskeletal organization | +4.41 |
| RAF1 | MAPK signaling – targets Troponin T (muscle) | +2.23 |
|  | | |
| *Ion Channels* | |  |
| ALDH9A1 | Aldehyde dehydrogenase to make GABA (opens ion channels) | +1.76 |
| *LGI1 | Neuronal voltage-gated potassium channel | +1.65 |
| *SCN4B | Modulates sodium gating kinetics | +1.49 |
| SLC12A5 | K^+^-Cl^-^ transporter important in neuronal ion homeostasis | +2.24 |
| SLC39A3 | Zn^+2^ influx carrier | +1.69 |
| SLC4A2 | Anion exchange protein | +1.77 |
|  | | |
| *Cellular Adhesion* | |  |
| AMOT | Plays central roll at tight junction maintenance | +1.60 |
| CLDN1 | Claudin - component of tight junctions | +2.38 |
| FAT | Atypical cadherin - possible adhesion or cell signaling | +3.18 |
| IGSF4 | Cell adhesion molecule in synapse assembly | +1.85 |
| SPON1 | Cell adhesion important for neuronal synapses | +5.61 |
|  | | |
| *Protein Folding/Turnover/Degradation* | |  |
| APOE | Binding, internalization, catabolism of lipoproteins | +3.40 |
| CNIH | Transport and maturation of TGFalpha | +4.42 |
| SNX27 | Promotes recycling of internalized transmembrane proteins | +3.19 |
| PSMD4 | Proteasome component - non-ATPase subunit of 19S lid | +2.75 |
| WFIKKN1 | Protease inhibitor | +2.64 |
|  | | |
| *Ca^+2^-dependent Function* | |  |
| *ATP2B2 | ATP-dependent calcium export | +1.93 |
| CACNA1A | Voltage sensitive Ca^+2^ channel - Ca^+2^ influx | +9.30 |
| CALU | Ca^+2^ binding protein involved in protein folding and sorting | +6.51 |
| *KCNN2 | Ca^+2^-activated potassium channel – regulates neuronal excitability | +2.96 |
|  | | |
| *Cell Growth/Survival/Cell Cycle Regulation* | |  |
| JUND | Jun family member - protects cells from p53 senescence | +2.05 |
| PDCD5 | Pro-apoptotic protein | +4.81 |
| WDR6 | Implicated in cell cycle growth arrest | +1.96 |
| ZNHIT1 | Involved with p53-mediated apoptosis | +1.86 |
|  | | |
| *Miscellaneous* | |  |
| ARHGEF3 | Nucleotide exchange factor for RhoA and RhoB | +8.83 |
| CYBRD1 | Ferroreductase | +4.75 |
| *HBA2 | Hemoglobin alpha – oxygen transport | +1.57 |
|  | | |
| *Glycolysis/Energy Metabolism* | |  |
| ADH7 | Alcohol dehydrogenase - metabolism of alcohol | +1.49 |
| *GK | Glycerol kinase - regulates glycerol uptake and metabolism | +1.69 |
| PGAM1 | Glycolytic enzyme | +4.64 |
|  | | |
| *Myogenic Functions* | |  |
| EHD1 | Membrane fusion involved in myoblast fusion | +2.43 |
| GREM1 | Antagonist of BMP signaling - promote muscle mass gain | +1.91 |
| SOX4 | Transcription factor involved with myoblast fusion | +1.88 |
|  | | |
| *Receptors* | |  |
| *ESR1 | Estrogen receptor - muscle/neuron growth and regeneration | +2.06 |
| KIT | Multifunctional receptor tyrosine kinase - STAT activation | +2.94 |
| *NPR3 | Natriuretic peptide receptor – regulate blood volume/pressure | +1.52 |
|  | | |
| *Transcriptional Regulation* | |  |
| BHLHB5 | Represses NeuroD1 and MyoD1-responsive genes | +2.22 |
| PURA | Transcriptional activator - Myc/possible replication initiation | +2.93 |
|  | | |
| *Actin/Microtubule Proteins* | |  |
| CAPZA2 | Caps F-actin - regulate actin polymerization (muscle contraction) | +2.69 |
| PFDN6 | Chaperone actin and tubulin for polymerization | +2.24 |
|  | | |
| *Extracellular Matrix* | |  |
| KIF13B | Kinesin - involved in reorganization of cytoskeleton | +12.00 |
| CORO1B | Regulates cell motility | +2.96 |
|  | | |
| *Kinases/Phosphatases* | |  |
| PRKACA | Catalytic subunit of cAMP-dependent kinase | +3.29 |
| PTPN11 | Multifunctional protein tyrosine phosphatase | +1.62 |
|  | | |
| *Mitochondrial Function* | |  |
| MTRF1L | Directs termination of mitochondrial protein translation | +1.68 |
| TOMM34 | Import of precursor proteins into mitochondria | +5.95 |
|  | | |
| *Ubiquitination/SUMOylation* | |  |
| CNOT4 | E3 ubiquitin ligase - activates Jak/STAT pathway | +3.24 |
| UBE2D2 | Catalyzes covalent attachment of ubiquitin to proteins | 1.95 |

*Gene name, function and fold change vs. SUC/SIV for genes with alterations in promoter methylation are listed. Genes with decreased promoter methylation expression and increased methylation expression respectively are grouped into specific biological categories.*
